# Genome-wide and transcriptome-wide association studies of mammographic density phenotypes reveal novel loci

**DOI:** 10.1186/s13058-022-01524-0

**Published:** 2022-04-12

**Authors:** Hongjie Chen, Shaoqi Fan, Jennifer Stone, Deborah J. Thompson, Julie Douglas, Shuai Li, Christopher Scott, Manjeet K. Bolla, Qin Wang, Joe Dennis, Kyriaki Michailidou, Christopher Li, Ulrike Peters, John L. Hopper, Melissa C. Southey, Tu Nguyen-Dumont, Tuong L. Nguyen, Peter A. Fasching, Annika Behrens, Gemma Cadby, Rachel A. Murphy, Kristan Aronson, Anthony Howell, Susan Astley, Fergus Couch, Janet Olson, Roger L. Milne, Graham G. Giles, Christopher A. Haiman, Gertraud Maskarinec, Stacey Winham, Esther M. John, Allison Kurian, Heather Eliassen, Irene Andrulis, D. Gareth Evans, William G. Newman, Per Hall, Kamila Czene, Anthony Swerdlow, Michael Jones, Marina Pollan, Pablo Fernandez-Navarro, Daniel S. McConnell, Vessela N. Kristensen, NBCS Investigators, Joseph H. Rothstein, Pei Wang, Laurel A. Habel, Weiva Sieh, Alison M. Dunning, Paul D. P. Pharoah, Douglas F. Easton, Gretchen L. Gierach, Rulla M. Tamimi, Celine M. Vachon, Sara Lindström

**Affiliations:** 1 Department of Epidemiology, School of Public Health, University of Washington, 3980 15th Ave NE, Box 351619, Seattle, WA 98195 USA; 2 Division of Cancer Epidemiology and Genetics, National Cancer Institute, Bethesda, MD USA; 3 School of Population and Global Health, University of Western Australia, Crawley, Australia; 4 Centre for Cancer Genetic Epidemiology, Department of Public Health and Primary Care, University of Cambridge, Cambridge, UK; 5 Department of Human Genetics, University of Michigan Medical School, Ann Arbor, MI USA; 6 Department of Mathematics and Statistics, Skidmore College, Saratoga Springs, NY USA; 7 Centre for Epidemiology and Biostatistics, School of Population and Global Health, The University of Melbourne, Melbourne, VIC Australia; 8 Precision Medicine, School of Clinical Sciences at Monash Health, Monash University, Clayton, VIC Australia; 9 Department of Health Sciences Research, Mayo Clinic, Rochester, MN USA; 10 Biostatistics Unit, The Cyprus Institute of Neurology and Genetics, Nicosia, Cyprus; 11 Cyprus School of Molecular Medicine, Nicosia, Cyprus; 12 Public Health Sciences Division, Fred Hutchinson Cancer Research Center, Seattle, WA USA; 13 Department of Gynecology and Obstetrics, Comprehensive Cancer Center ER-EMN, University Hospital Erlangen, Friedrich-Alexander University Erlangen-Nuremberg, Erlangen, Germany; 14 Cancer Control Research, BC Cancer and School of Population and Public Health, University of British Columbia, Vancouver, Canada; 15 Public Health Sciences, Queen’s University, Kingston, Canada; 16 Division of Cancer Sciences, University of Manchester, Manchester, UK; 17 Division of Informatics, Imaging and Data Sciences, University of Manchester, Manchester, UK; 18 Cancer Epidemiology Division, Cancer Council Victoria, Melbourne, VIC Australia; 19 Center for Genetic Epidemiology, Department of Preventive Medicine, Keck School of Medicine, University of Southern California, Los Angeles, CA USA; 20 Epidemiology Program, University of Hawaii Cancer Center, Honolulu, HI USA; 21 Department of Epidemiology and Population Health, Stanford University School of Medicine, Stanford, CA USA; 22 Department of Medicine (Oncology), Stanford University School of Medicine, Stanford, CA USA; 23 Stanford Cancer Institute, Stanford University School of Medicine, Stanford, CA USA; 24 Department of Epidemiology, Harvard T.H. Chan School of Public Health, Boston, MA USA; 25 Channing Division of Network Medicine, Department of Medicine, Brigham and Women’s Hospital and Harvard Medical School, Boston, MA USA; 26 Fred A. Litwin Center for Cancer Genetics, Lunenfeld-Tanenbaum Research Institute of Mount Sinai Hospital, Toronto, Canada; 27 Department of Molecular Genetics, University of Toronto, Toronto, Canada; 28 Division of Evolution and Genomic Medicine, School of Biological Sciences, Faculty of Biology, Medicine and Health, Manchester Academic Health Science Centre, University of Manchester, Manchester, UK; 29 Genomic Medicine, St Mary’s Hospital, Manchester Centre for Genomic Medicine, Manchester University Hospitals NHS Foundation Trust, Manchester Academic Health Science Centre, Manchester, UK; 30 NIHR Manchester Biomedical Research Centre, Manchester Academic Health Science Centre, Manchester University NHS Foundation Trust, Manchester, UK; 31 Department of Medical Epidemiology and Biostatistics, Karolinska Institutet, Stockholm, Sweden; 32 Division of Genetics and Epidemiology, The Institute of Cancer Research, London, UK; 33 Cancer and Environmental Epidemiology Unit, National Center for Epidemiology, Carlos III Institute of Health, Madrid, Spain; 34 Department of Epidemiology, School of Public Health, University of Michigan, Ann Arbor, MI USA; 35 Department of Medical Genetics, Oslo University Hospital and University of Oslo, Oslo, Norway; 36 Department of Population Health Science and Policy, Icahn School of Medicine at Mount Sinai, New York, NY USA; 37 Department of Genetics and Genomic Sciences, Icahn School of Medicine at Mount Sinai, New York, NY USA; 38 Division of Research, Kaiser Permanente Northern California, Oakland, CA USA; 39 Division of Epidemiology, Population Health Science, Weill Cornell Medicine, New York, NY USA

**Keywords:** Mammographic density, Breast cancer, Genome-wide association study (GWAS), Transcriptome-wide association study (TWAS)

## Abstract

**Background:**

Mammographic density (MD) phenotypes, including percent density (PMD), area of dense tissue (DA), and area of non-dense tissue (NDA), are associated with breast cancer risk. Twin studies suggest that MD phenotypes are highly heritable. However, only a small proportion of their variance is explained by identified genetic variants.

**Methods:**

We conducted a genome-wide association study, as well as a transcriptome-wide association study (TWAS), of age- and BMI-adjusted DA, NDA, and PMD in up to 27,900 European-ancestry women from the MODE/BCAC consortia.

**Results:**

We identified 28 genome-wide significant loci for MD phenotypes, including nine novel signals (5q11.2, 5q14.1, 5q31.1, 5q33.3, 5q35.1, 7p11.2, 8q24.13, 12p11.2, 16q12.2). Further, 45% of all known breast cancer SNPs were associated with at least one MD phenotype at *p* < 0.05. TWAS further identified two novel genes (*SHOX2* and *CRISPLD2*) whose genetically predicted expression was significantly associated with MD phenotypes.

**Conclusions:**

Our findings provided novel insight into the genetic background of MD phenotypes, and further demonstrated their shared genetic basis with breast cancer.

**Supplementary Information:**

The online version contains supplementary material available at 10.1186/s13058-022-01524-0.

## Background

Heterogeneity of breast tissue composition can be observed through radiographic imaging using mammography. Epithelial and connective tissues are radiologically dense with white appearance, while adipose tissue is radiologically lucent with dark appearance on a mammogram [1]. Mammographic density (MD) has been widely established as one of the strongest risk factors for breast cancer [2–4], the most common cancer type among women in the USA [5]. Specifically, quantitative MD measures, including mammographic dense area (DA), non-dense area (NDA), and the percentage of dense area in the whole breast (PMD), have all been independently associated with breast cancer [3, 6–8]. In analyses adjusting for body mass index (BMI) and age, women with higher DA and PMD have an elevated risk of breast cancer, while NDA is associated with a decreased breast cancer risk.

Twin studies indicate that genetic factors explain a large fraction of the variation in MD phenotypes, with heritability estimates for DA, NDA, and PMD, after adjusting for age and individual-specific shared environmental factors, exceeding 60% [9–11]. Previous genome-wide association studies (GWAS) have identified 46 genetic variants that are significantly (*p* < 5 × 10^−8^) associated with at least one MD phenotype, including 27 associated with DA, 17 associated with NDA, and 20 associated with PMD [12–17]. Importantly, many of these variants have also been discovered as the susceptible loci of breast cancer, suggesting the critical role played by MD as an intermediate phenotype for the disease. However, only a small fraction of the variance of MD phenotypes can be explained by these significant variants [14, 17]. To enhance our understanding of the genetic basis of MD, additional GWAS with larger sample sizes is needed.

In the present study, we conducted GWAS and a transcriptome-wide association study (TWAS) in up to 27,900 European ancestry women with the goal of identifying novel loci associated with MD phenotypes.

## Methods

### Study population and data collection

We conducted a GWAS for three MD phenotypes (DA, NDA, and PMD) using data from 21 studies which provided individual-level genotype and phenotype data (Additional file 2: Table S1) as well as nine additional studies which provided GWAS summary statistics (Additional file 2: Table S2), under the Breast Cancer Association Consortium (BCAC) and the Markers of Density Consortium (MODE). The overall study sample comprised of 6666 breast cancer cases and 21,234 controls. All the individuals had PMD data collected, while the DA and NDA measures were only available in a proportion of the study population. The final sample sizes used in the meta-analyses were 24,579 (DA), 24,689 (NDA), and 27,900 (PMD). For breast cancer cases, mammograms collected prior to the cancer diagnosis were used for density assessment. Study-specific approaches to obtain quantitative measures of MD phenotypes are summarized in Additional file 2: Table S1, and study-specific protocols for MD measurement are given in the Additional file 1. Most of studies included in our analysis used *CUMULUS*, a computer-assisted semi-automated thresholding software [18]. Age and BMI at time of mammogram collection were included as covariates in the GWAS. For participants with missing BMI at mammogram (*N* = 1767), self-reported BMI within five years of mammogram collection was used as an approximation.

Individual-level genotype data were generated with either the iCOGs [19] or OncoArray [20] arrays. We applied standard quality control filters as described elsewhere [19]. Genotype data were imputed to 1000 Genomes phase 3 version 5 using *IMPUTE2* [21]. Genotype dosage, ranging between 0 and 2, was generated for imputed variants. Single nucleotide polymorphisms (SNPs) with low imputation quality (INFO < 0.3) or with a minor allele frequency (MAF) < 1% were excluded. Approximately 9.8 million variants were included in the association analysis. Genomic positions of the variants were based on Genome Reference Consortium GRCh37 (hg19).

### Genome-wide association study (GWAS)

We conducted study-specific multivariable adjusted linear regression analysis for each MD phenotype. All MD phenotypes were square-root transformed before analysis as this resulted in distributions that were close to normal. Age and 1/BMI at mammogram, and the first ten ancestry informative principal components, as previously described [14], were included as covariates in each regression model. Analyses were performed using R 3.6.1 (R Foundation). We then combined study-specific GWAS results with previously derived GWAS summary statistics using a sample-size weighted meta-analysis (the ‘SAMPLESIZE’ scheme as implemented in *METAL* [22]). To be included in the meta-analysis, a variant needed to have a valid Z-statistic from at least three individual studies and a minimum sample size of 3000. Regional association plot for each genome-wide significant locus in the meta-analysis was generated using the *LocusZoom* software [23].

### Sensitivity and conditional analysis

The majority of studies included in our analysis were population-based or breast cancer nested case–control studies. To assess if any identified SNP-MD associations was an artifact resulting from oversampling of breast cancer cases in our population, we replicated the association analysis for all genome-wide significant SNPs in controls only (*N* = 21,234), as a sensitivity analysis. As mammographic NDA is strongly associated with BMI, we also assessed the association between significant NDA loci and BMI among 13,915 individuals with NDA, BMI, and genotype data available.

To quantify the number of independent signals in each significant GWAS locus, we performed a conditional analysis using the COndition and JOint analysis tool implemented in the Genome-wide Complex Trait Analysis software *(COJO-GCTA)* [24]. Since *COJO-GCTA* requires beta and standard error estimates, which were not available in our sample-size weighted meta-analysis data, we performed a standard error weighted meta-analysis with the normalized square-rooted MD phenotypes (per study, [sqrt-MD − mean(sqrt-MD)]/stderr(sqrt-MD)) as the outcomes. For each locus, we defined the lead SNP as the first independent signal, and performed the conditional analysis for SNPs located within + / − 500 kb. The top-ranked SNP with conditional *p* value < 10^−5^ was added to the independent signal list, and the conditional analysis was run again for rest of the SNPs. The conditional analysis was halted when no variant reached the threshold of conditional *p* value < 10^−5^. For the loci with multiple independent signals identified in the conditional analysis, all signals are annotated on the regional association plot.

### Breast cancer association analysis

We examined the association between MD phenotype-associated SNPs and breast cancer risk, overall and by estrogen receptor (ER) status, using publicly available breast cancer GWAS summary statistics [19], based on 122,977 cases (including 69,501 ER-positive and 21,468 ER-negative cases) and 105,974 controls of European ancestry from the BCAC. We also assessed if known breast cancer SNPs [25] were associated with MD phenotypes.

### Exploratory bioinformatics analysis

We used linkage disequilibrium (LD) score regression to estimate the SNP heritability (*h*^2^_SNP_) of MD phenotypes [26]. We partitioned the *h*^2^_SNP_ by 74 functional genomic categories [27], and estimated the heritability enrichment for each category. We quantified the genome-wide genetic correlation between each MD phenotype and breast cancer [19, 28]. We also estimated the local genetic correlation between MD phenotypes and overall breast cancer using ρ*HESS* [29, 30], which estimates the local shared heritability between two traits across 1703 independent genomic blocks, based on LD in European ancestry populations [31]. We defined statistically significant local genetic correlations as *p* < 0.05/1703 = 2.94 × 10^−5^.

### Transcriptome-wide association study (TWAS)

To estimate the association between imputed gene expression and MD phenotypes, we conducted a transcriptome-wide association analysis (TWAS). We used genotype and gene expression data in mammary tissue from 396 individuals collected by the GTEx consortium (Release V8) to build gene-specific SNP prediction models of gene expression [32]. Predictive models were built based on variants located + / − 500 kb of each gene, using three different approaches (Top 1, Elastic Net [33], and LASSO [34]). Gene-specific expression levels were then imputed with the model showing the highest predictive R-square based on cross-validation. A total of 7284 genes with nominally significant (*p* < 0.01) heritability were included in the association analysis for each MD phenotype. Construction of predictive models and association analysis using GWAS summary statistics were performed using the R-based pipeline *FUSION* [34]. A significance threshold of *p* < 0.05/(7284*3) = 2.29 × 10^−6^ was utilized to identify statistically significant associations between imputed gene expression levels and MD phenotypes.

### Replication of novel GWAS and TWAS findings

Replication analyses of the novel GWAS loci were performed using data from a previous GWAS meta-analysis of mammographic density phenotypes in an independent population of 24,192 European ancestry women participating in the Kaiser Permanente Northern California (KPNC) Research Program on Genes, Environment and Health (RPGEH) [17]. Briefly, MD phenotypes were measured using Cumulus6 on a single craniocaudal view from 20,311 Hologic and 3881 GE full-field digital mammography (FFDM) exams. MD phenotypes were transformed separately within each cohort to attain standard normal distributions and to facilitate meta-analysis and interpretation of effect sizes in SD units. Genotypes were assayed using the Affymetrix Axiom array with > 650,000 variants, and imputed using the 1000 Genomes Project Phase III reference panel. Allele dosage effects were estimated using linear regression models adjusted for age at mammography, ln(BMI), the first ten principal components of European ancestry, genotyping reagent kit, and image batch separately in the Hologic and GE cohorts, and the estimates were combined using inverse-variance weighted meta-analysis.

Replication analyses of the novel TWAS loci were performed in 24,158 women from the Kaiser RPGEH mammographic density GWAS [17] with genotypes imputed using the Haplotype Reference Consortium reference panel for single-nucleotide variants, and 1000 Genomes Project Phase III reference panel for indels [35]. Expression levels of 7 genes (*MTMR11, SHOX2, CRISPLD2, SMIM25, TMEM184B, EP300,* and *DESI1*) were estimated using the PredictDB GTEx v8 Elastic Net models for mammary tissue [33], which did not include *MRPL23-AS1*. Associations of the predicted gene expression levels with the standardized MD phenotypes were estimated using linear regression models adjusted for age at mammography, ln(BMI), the first ten principal components of European ancestry, genotyping reagent kit, and image batch separately in the Hologic (*n* = 20,282) and GE (*n* = 3876) cohorts, and the estimates were combined using inverse-variance weighted meta-analysis.

## Results

Our study population was on average 56.6 years old and had an average BMI of 26.5 kg/m^2^ at the time of mammogram. The mean DA, NDA, and PMD were 28.5 cm^2^, 120.4 cm^2^, and 23.4%, respectively. Age and BMI at mammogram, as well as the square-root transformed MD measures, all approximately followed a normal distribution (Additional file 2: Figure S1). Genomic inflation factors (*λ*_GC_) were between 1.11 and 1.13 (Additional file 2: Figure S2), with LD-score regression intercepts between 1.05 and 1.06, suggesting that the observed genomic inflation is partly driven by the polygenic effects of many variants [26].

### GWAS of MD phenotypes

**Fig. 1:**
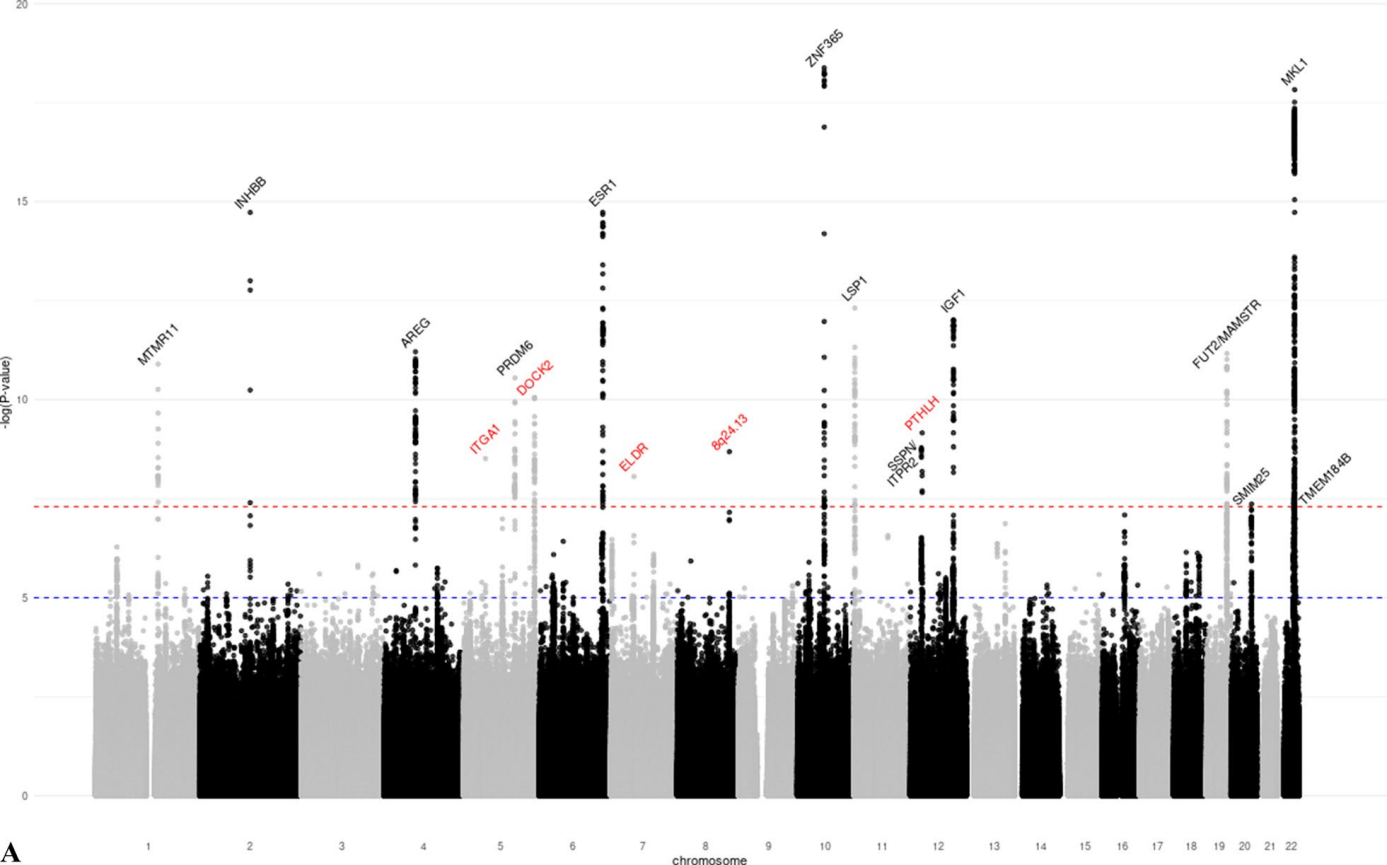

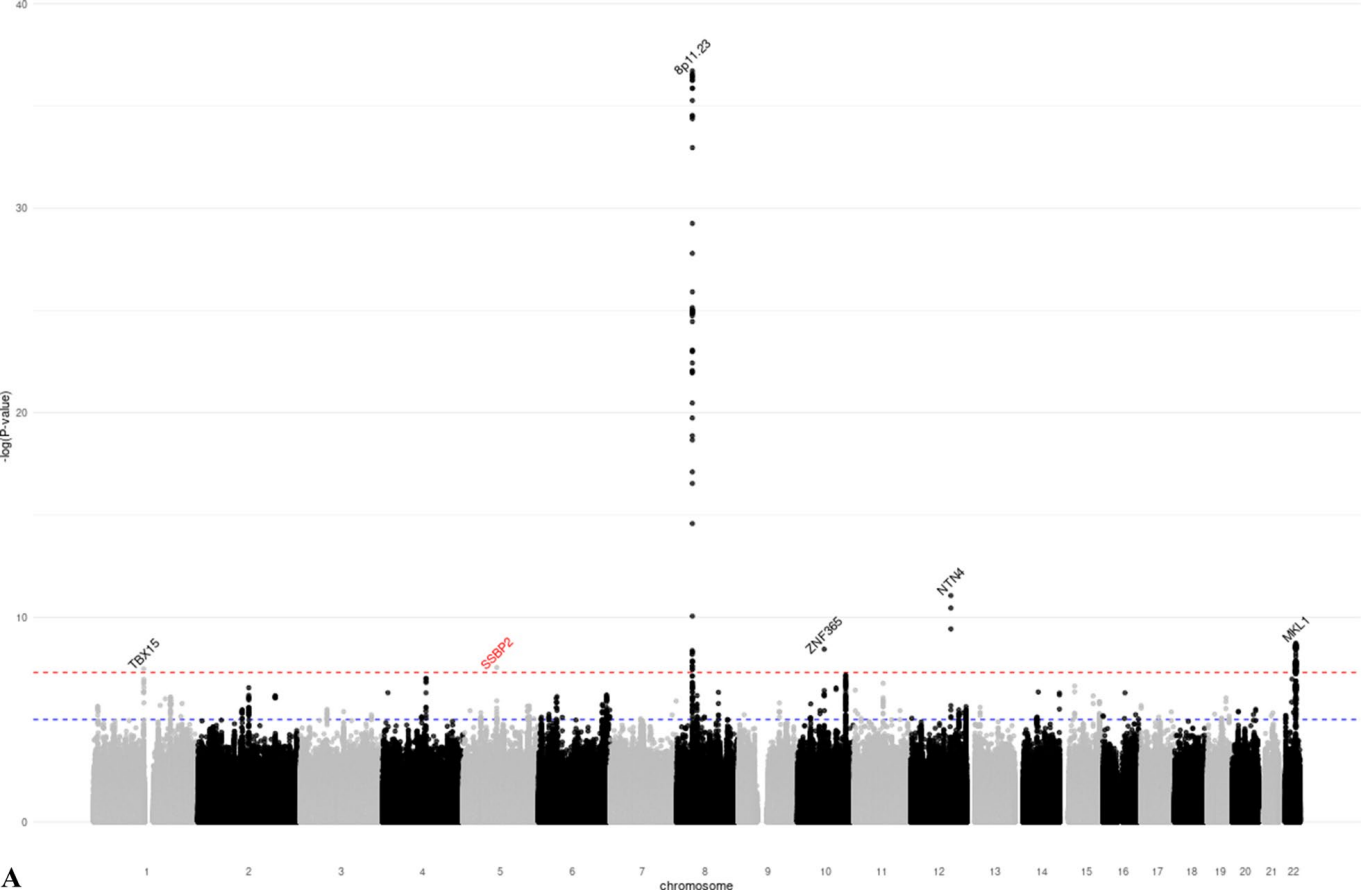

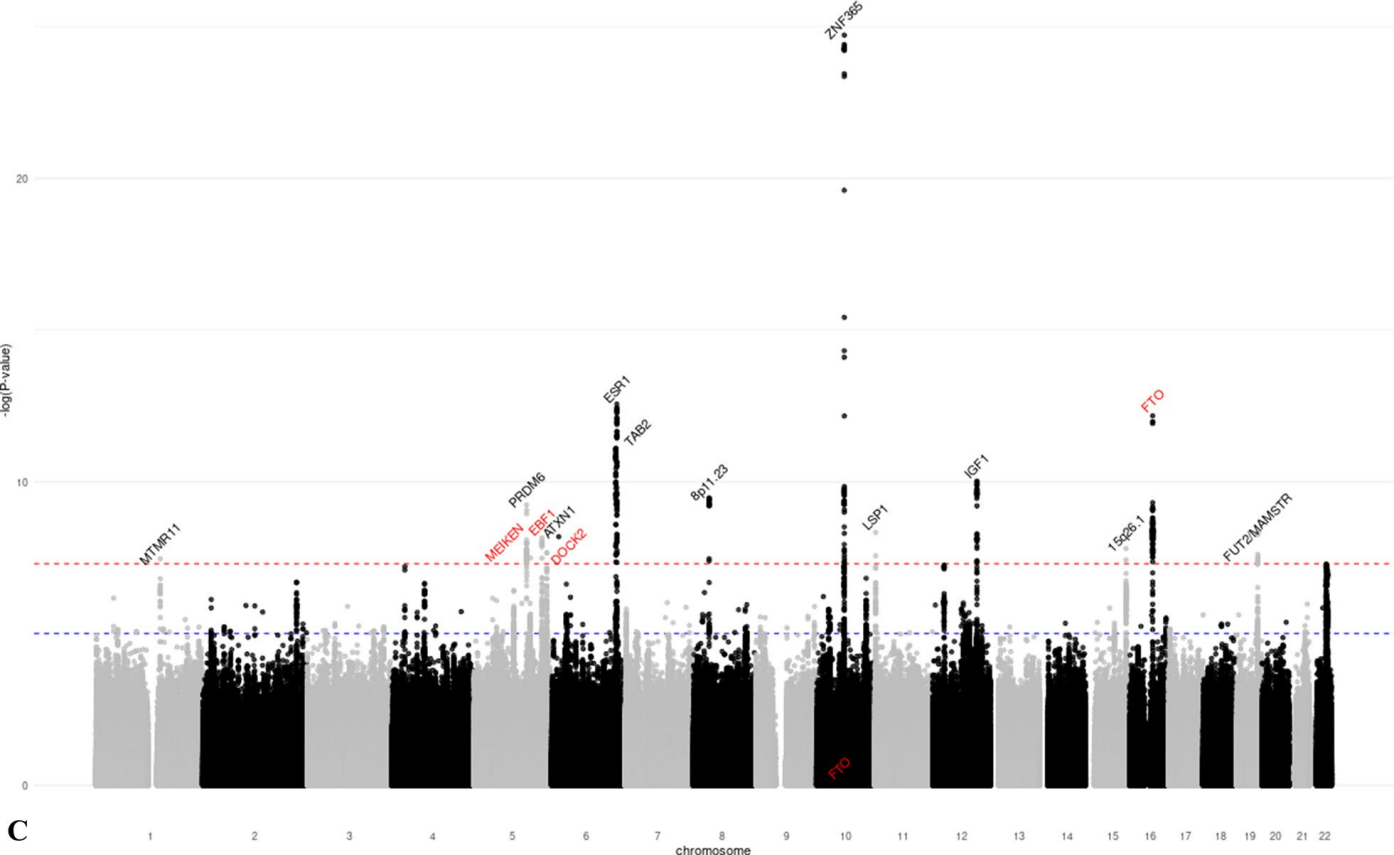
Manhattan plots of the GWAS meta-analysis results of mammographic **a** dense area (DA, *N* = 24,579), **b** non-dense area (NDA, *N* = 24,689), and **c** percent mammographic density (PMD, *N* = 27,900). *p* value thresholds for genome-wide significance (*p* = 5 × 10^−8^, red dash line) and suggestive significance (*p* = 10^−5^, blue dash line) are shown as horizontal lines. The gene closest to each lead variant is annotated. Novel loci are marked red. **a** Manhattan plot of the GWAS meta-analysis results of DA. **b** Manhattan plot of the GWAS meta-analysis results of NDA. **c** Manhattan plot of the GWAS meta-analysis results of PMD

We identified 28 distinct loci associated with at least one MD phenotype at *p* < 5 × 10^−8^ (Table 1, Additional file 1: Figures S3–S8). Of these, 18 were associated with DA, six with NDA, and 15 with PMD (Fig. 1). SNPs in seven regions (1q21.2, 5q23.2, 5p35.1, 6q25.1, 11p15.5, 12q23.2, 19q13.33) were associated with both DA and PMD. SNPs at 8q11.23 were associated with both NDA and PMD; SNPs at 22q13.2 were associated with both DA and NDA; and SNPs at 10q21.2 were associated with all three MD phenotypes. The phenotypic variance explained by the lead SNPs of the genome-wide significant loci was 2.6% for DA, 0.8% for NDA, and 1.6% for PMD. Nine of the significant loci (5q11.2, 5q14.1, 5q31.1, 5q33.3, 5q35.1, 7p11.2, 8q24.13, 12p11.2, 16q12.2) had not previously been associated with MD phenotypes. Conditional analysis showed evidence that four DA-associated loci (5q35.1, 10q21.2, 20q13.13, 22q13.2) and one NDA-associated locus (8p11.23) had two independent signals at conditional *p* value < 10^−5^ (Additional file 2: Table S3).

**Table 1:** Lead SNPs of the genome-wide significant loci identified in the GWAS meta-analysis of mammographic dense area (DA), non-dense area (NDA) and percent mammographic density (PMD)

Region	rs ID	Chromosome	Position^1^	Gene^2^	Alleles^3^	MAF^4^	Z-score^5^	*p* Value	Novel locus^6^
*DA (N* = *18)*									
1q21.2	rs11205303	1	149,906,413	MTMR11	T/C	0.369	− 6.772	1.25E−11	No
2q14.2	rs17625845	2	121,089,731	INHBB	T/C	0.221	7.949	1.87 E−15	No
4q13.3	rs6851733	4	75,518,708	AREG	T/C	0.226	6.874	6.23 E−12	No
5q11.2	rs150249911	5	52,119,132	ITGA1	A/G	0.018	− 5.928	3.06 E−09	Yes
5q23.2	rs335189	5	122,446,856	PRDM6	C/G	0.265	6.655	2.84 E−11	No
5q35.1	rs2042239	5	169,566,329	DOCK2	A/G	0.354	6.485	8.87 E−09	Yes
6q25.1	rs9397436	6	151,952,002	ESR1	A/G	0.073	7.951	1.86 E−15	No
7p11.2	rs10155920	7	55,308,930	ELDR	T/C	0.145	5.755	8.66 E−09	Yes
8q24.13	rs58847541	8	124,610,166	–	A/G	0.153	5.994	2.05 E−09	Yes
10q21.2	rs10995187	10	64,273,026	ZNF365	A/G	0.148	8.933	4.14 E−19	No
11p15.5	rs4980383	11	1,902,097	LSP1	T/C	0.451	− 7.228	4.90 E−13	No
12p12.1	rs11836164	12	26,446,625	SSPN/ITPR2	T/C	0.248	6.028	1.66 E−09	No
12p11.2	rs7297051	12	28,174,817	PTHLH	T/C	0.233	6.166	7.01 E−10	Yes
12q23.2	rs833472	12	103,005,100	IGF1	T/C	0.032	7.134	9.76 E−13	No
19q13.33	rs1231281	19	49,239,200	FUT2/MAMSTR	A/G	0.458	6.860	6.87 E−12	No
20q13.13	rs17789629	20	48,892,374	SMIM25	A/C	0.137	− 5.478	4.30 E−08	No
22q13.1	rs34066050	22	38,612,604	TMEM184B	A/G	0.467	− 5.534	3.14 E−08	No
22q13.2	rs6001939	22	40,892,794	MKL1	T/C	0.102	8.791	1.48 E−18	No
*NDA (N* = *6)*									
1p12	rs78395856	1	119,495,096	TBX15	A/C	0.046	5.525	3.29 E−08	No
5q14.1	rs413472	5	80,930,992	SSBP2	T/C	0.445	5.551	2.84 E−08	Yes
8p11.23	rs16885613	8	36,848,357	–	T/C	0.148	− 12.788	1.92 E−37	No
10q21.2	rs2138555	10	64,220,494	ZNF365	A/G	0.419	5.900	3.63 E−09	No
12q22	rs61938093	12	96,026,737	NTN4	T/C	0.290	− 6.266	3.72 E−10	No
22q13.2	rs73169097	22	41,027,870	MKL1	T/C	0.100	6.012	1.83 E−09	No
*PMD (N* = *15)*									
1q21.2	rs1868992	1	149,908,108	MTMR11	A/G	0.259	5.517	3.44 E−08	No
5q23.2	rs335189	5	122,446,856	PRDM6	C/G	0.265	6.196	5.78 E−10	No
5q31.1	rs76876329	5	131,237,759	MEIKIN	T/C	0.151	5.530	3.21 E−08	Yes
5q33.3	rs11745230	5	158,171,008	EBF1	T/G	0.166	− 5.793	6.90 E−09	Yes
5q35.1	rs2112670	5	169,557,594	DOCK2	A/G	0.345	− 5.598	2.17 E−08	Yes
6p22.3	rs3819405	6	16,399,557	ATXN1	T/C	0.340	5.806	6.42 E−09	No
6q25.1	rs4897107	6	149,601,591	TAB2	T/C	0.162	6.842	7.81 E−12	No
6q25.1	rs9397436	6	151,952,002	ESR1	A/G	0.073	7.303	2.81 E−13	No
8p11.23	rs10087804	8	36,858,140	–	C/G	0.142	6.282	3.35 E−10	No
10q21.2	rs10995187	10	64,273,026	ZNF365	A/G	0.148	10.424	1.92 E−25	No
11p15.5	rs4980383	11	1,902,097	LSP1	T/C	0.451	− 5.860	4.62 E−09	No
12q23.2	rs61941038	12	102,989,316	IGF1	A/T	0.033	− 6.477	9.37 E−11	No
15q26.1	rs4499190	15	94,275,057	–	G/C	0.341	− 5.654	1.57 E−08	No
16q12.2	rs11646715	16	53,824,007	FTO	A/G	0.456	7.183	6.84 E−13	Yes
19q13.33	rs12462111	19	49,171,306	FUT2/MAMSTR	T/C	0.427	5.847	5.02 E−09	No

^1^Genomic positions based on build GRCh37/hg19

^2^Closest gene to the lead variant

^3^Coded as reference allele/effect allele

^4^Minor allele frequency, based on the European Ancestry population in the 1000 Genome project

^5^Z-scores were obtained using sample size weighted meta-analysis of GWAS. The multivariate linear regression model used in GWAS analysis adjusted for age and BMI at mammogram, as well as the first ten principal components representing population structure. Additive inheritance model was used. Square-root transformed mammographic density phenotypes were used as the outcome variable

^6^Whether the locus is novel (Yes) or has previously been reported (No) to be associated with at least one MD phenotype in the literature

For the 10 novel SNPs identified (corresponding to nine loci as the 5q35.1 region was associated with both DA and PMD), we performed look-ups using 24,192 women of European ancestry [17]. Of the ten SNPs, seven replicated at *p* < 0.05 with the same direction of effect (three with DA (5q35.1, 8q24.13, 12p11.2) and four with PMD (5q31.1, 5q33.3, 5q35.1, 16q12.2), Additional file 2: Table S4). One DA SNP (5q11.2) had a concordant direction of association, whereas one DA SNP (7p11.2) and the NDA SNP (5q14.1) had discordant directions of association in the look-up with *p* > 0.05.

Sensitivity analysis based on 21,234 controls showed consistent direction of effect and comparable effect size to the main analysis (Additional file 2: Table S5). None of the NDA-associated SNPs were associated with BMI at *p* < 0.05 (Additional file 2: Table S6), suggesting that observed SNP-NDA associations were not due to residual confounding with BMI.

We captured the lead SNPs from 46 distinct loci that have previously been reported to associate with at least one MD phenotype. We investigated their associations (*N* = 63) with the corresponding MD phenotype based on our study (Additional file 2: Table S7). We were able to replicate 26 out of 28 DA SNPs, 13 out of 16 NDA SNPs, and 15 out of 19 PMD SNPs at *p* < 0.05. Among these, 10 DA SNPs, 2 NDA SNPs, and 5 PMD SNPs were found with genome-wide significance at *p* < 5 × 10^−8^.

### Association of MD significant loci with breast cancer

**Fig. 2:**
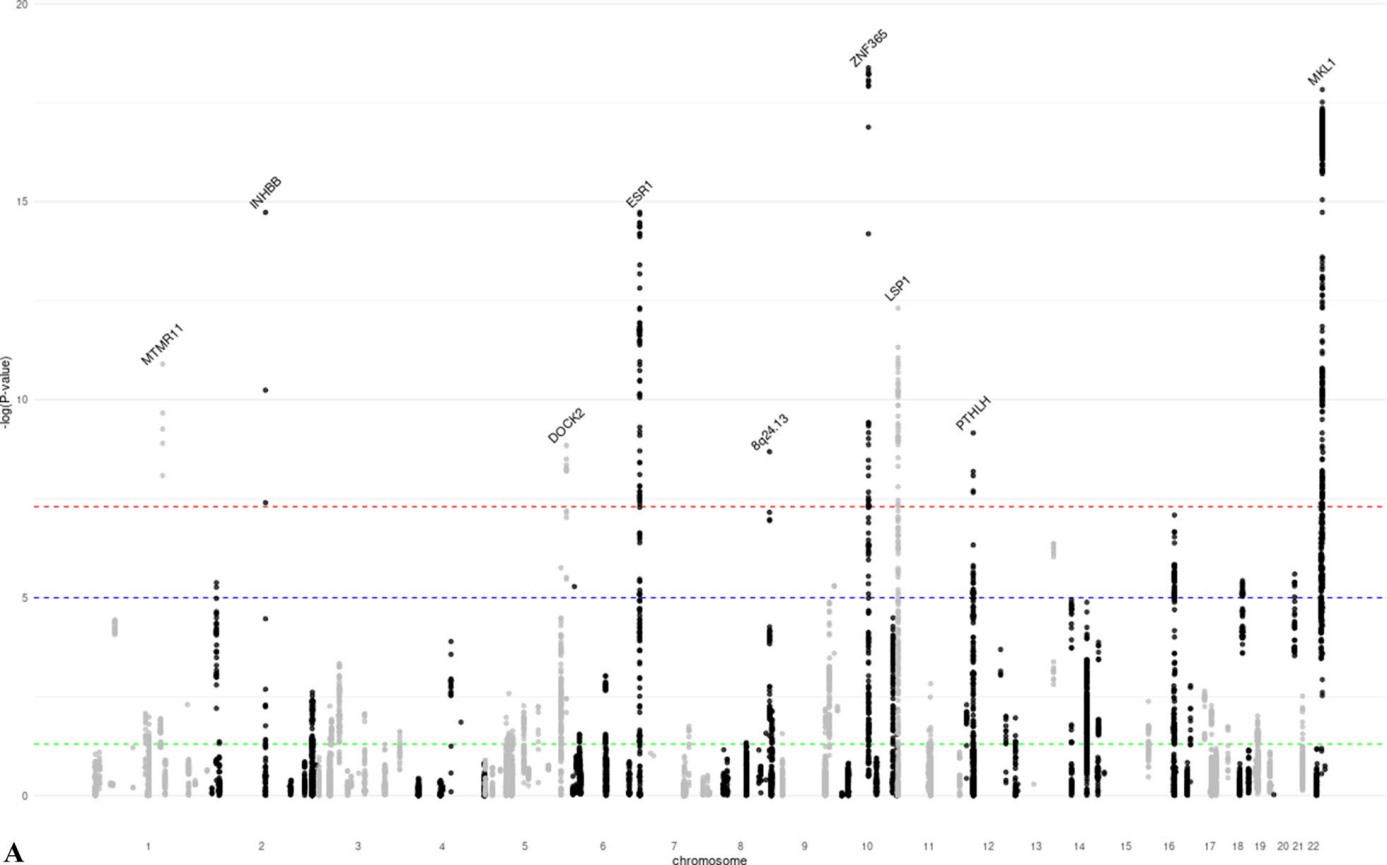

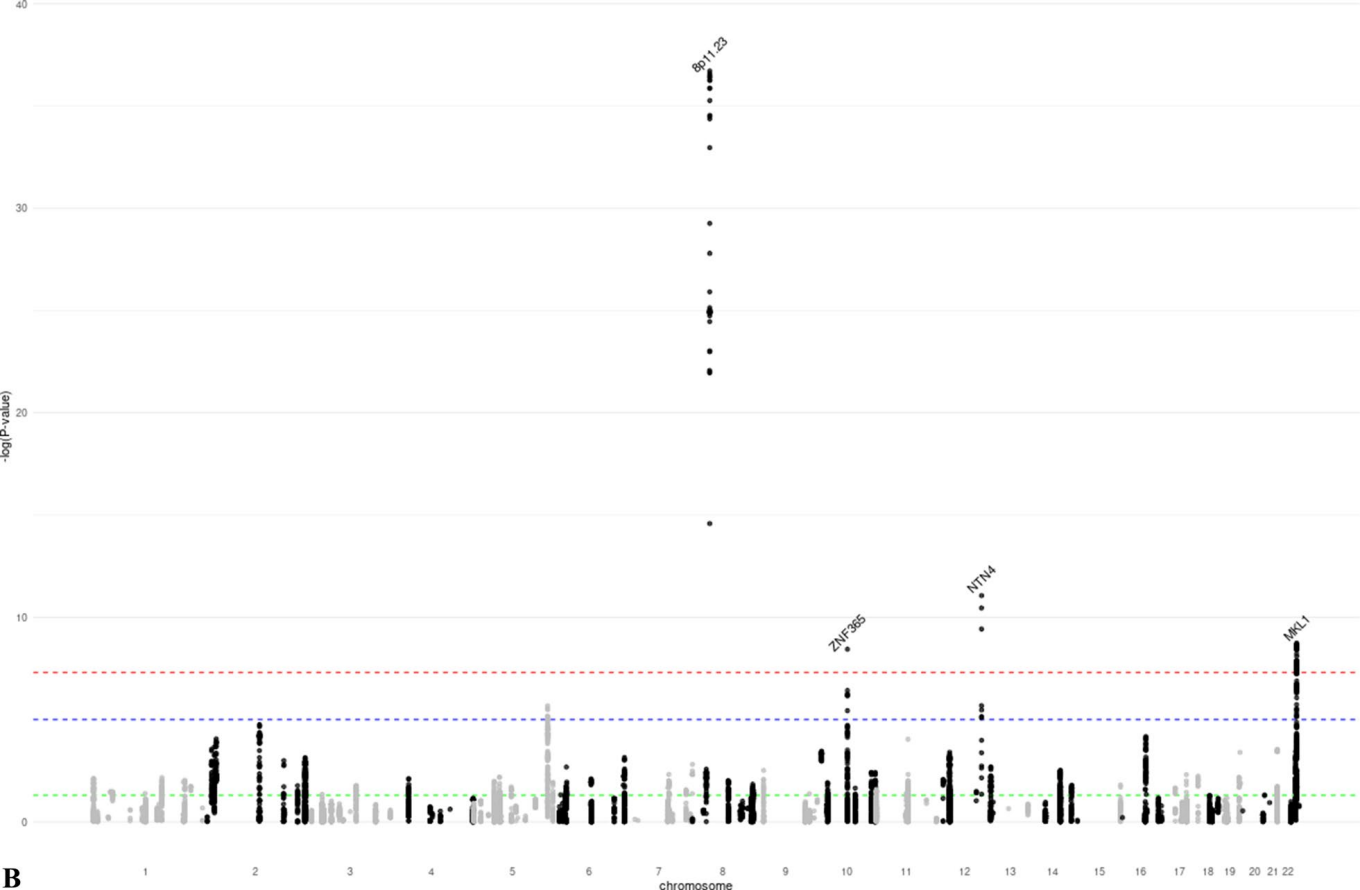

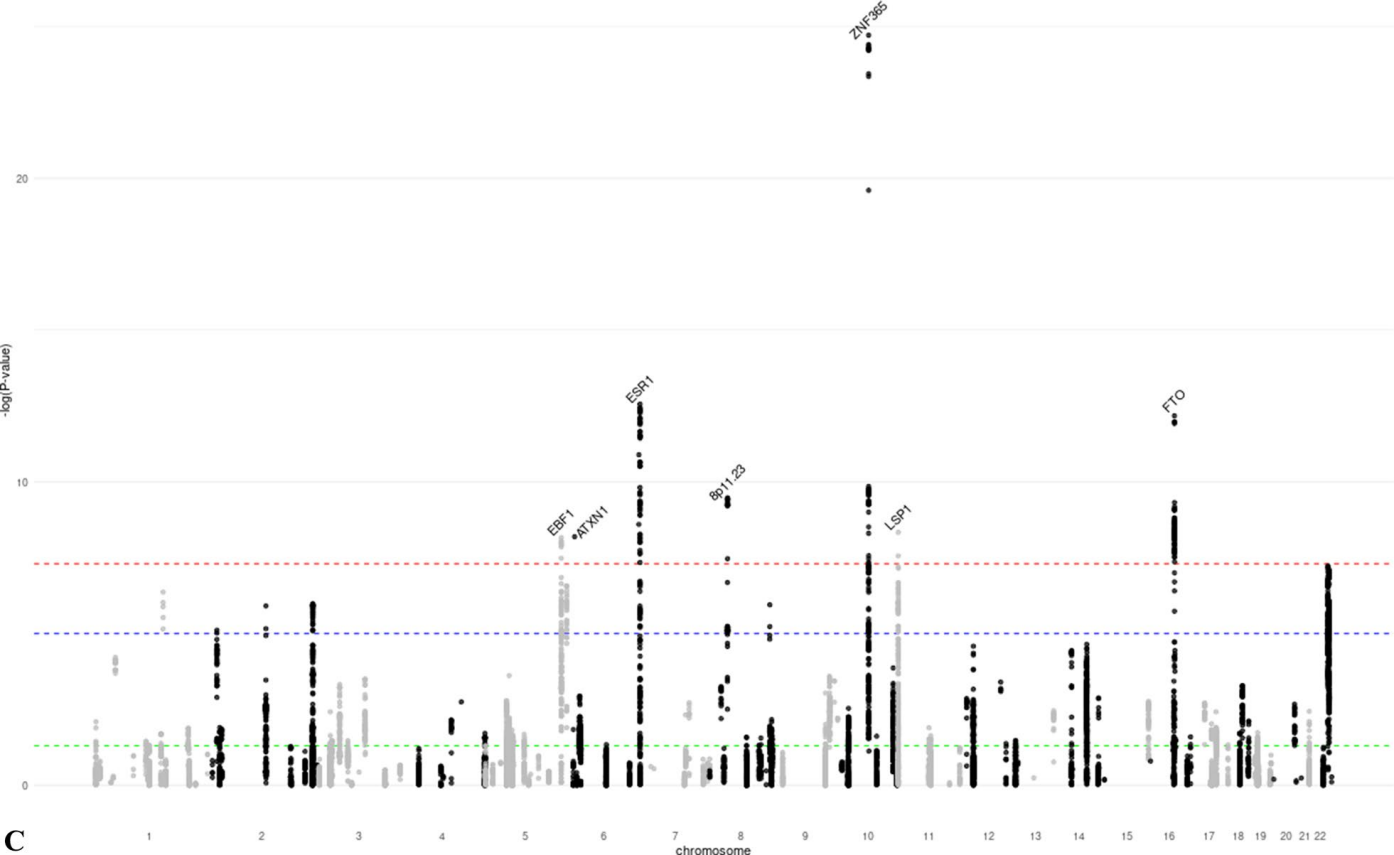
Manhattan-like plots showing the association between genome-wide significant breast cancer SNPs and the three mammographic density phenotypes (DA, NDA, PMD). *p* value thresholds for genome-wide significance (*p* = 5 × 10^−8^, red line), suggestive significance (*p* = 10^−5^, blue line) and nominal significance (*p* = 0.05, green line) are shown as horizontal dash lines. For signals with genome-wide significance for both MD phenotype and breast cancer, the nearest gene is annotated. **a** GWAS results of DA for significant SNPs of breast cancer. **b** GWAS results of NDA for significant SNPs of breast cancer. **c** GWAS results of PMD for significant SNPs of breast cancer

We assessed whether the identified MD phenotype-associated SNPs were also associated with breast cancer risk. Of the 28 lead SNPs, 13 were associated with overall breast cancer risk at genome-wide significance (Table 2). In addition, one SNP (22q13.1) was significantly associated with ER-positive breast cancer (*p* = 5.6 × 10^−8^ for overall breast cancer). For nine of these 14 SNPs, the direction of association was consistent with that expected (i.e., the same direction for DA, PMD and breast cancer risk, and opposite direction for NDA and breast cancer risk), while for five SNPs the direction was the opposite; some of these conflicting results have been observed previously [14]. An additional seven lead SNPs were associated with breast cancer risk at *p* < 0.05. We also assessed the associations between 205 independent genome-wide significant variants for breast cancer [19] and MD phenotypes (Fig. 2, Additional file 2: Table S8) at *p* < 0.05, 63 (31%, 48 with consistent direction as expected) breast cancer variants were associated with DA, 36 (18%, 20 with opposite direction as expected) with NDA, and 62 (30%, 49 with consistent direction as expected) with PMD, respectively. In total, 92 (45%, 67 with expected direction) breast cancer variants were associated with at least one MD phenotype.

**Table 2:** Association between MD significant loci and breast cancer risk (overall, ER-positive, and ER-negative)

Region	rsID	CHR	Position^1^	Allele^2^	MD phenotype		Overall breast cancer^4^			ER-positive breast cancer^4^			ER-negative breast cancer^4^		
					*Z*-score	*p* Value	Beta	SE^3^	*p* Value	Beta	SE	*p* Value	Beta	SE	*p* Value
*DA*															
1q21.2	rs11205303	1	149,906,413	T/C	− 6.772	1.25 E−11	0.050	0.006	1.05 E−14	0.057	0.008	8.25 E−14	0.040	0.012	6.67 E−04
2q14.2	rs17625845	2	121,089,731	T/C	7.949	1.87 E−15	− 0.046	0.008	1.73 E−08	− 0.032	0.010	8.94 E−04	− 0.114	0.015	1.13 E−13
4q13.3	rs6851733	4	75,518,708	T/C	6.874	6.23 E−12	0.009	0.008	2.20 E−01	0.001	0.009	9.58 E−01	0.018	0.014	1.97 E−01
5q11.2	rs150249911	5	52,119,132	A/G	− 5.928	3.06 E−09	0.038	0.027	1.49 E−01	0.048	0.032	1.33 E−01	0.053	0.050	2.87 E−01
5q23.2	rs335189	5	122,446,856	C/G	6.655	2.84 E−11	0.027	0.007	8.38 E−05	0.026	0.008	1.46 E−03	0.012	0.012	3.32 E−01
5q35.1	rs2042239	5	169,566,329	A/G	6.485	8.87 E−09	0.028	0.006	9.80 E−06	0.037	0.008	1.02 E−06	0.003	0.012	7.72 E−01
6q25.1	rs9397436	6	151,952,002	A/G	7.951	1.86 E−15	0.184	0.012	2.00 E−54	0.138	0.014	1.71 E−22	0.272	0.021	4.01 E−40
7p11.2	rs10155920	7	55,308,930	T/C	5.755	8.66 E−09	0.011	0.008	2.01 E−01	0.012	0.010	2.50 E−01	− 0.001	0.015	9.35 E−01
8q24.13	rs58847541	8	124,610,166	A/G	5.994	2.05 E−09	− 0.063	0.009	4.33 E−13	− 0.055	0.010	1.17 E−07	− 0.077	0.016	1.00 E−06
10q21.2	rs10995187	10	64,273,026	A/G	8.933	4.14 E−19	0.129	0.009	2.85 E−49	0.134	0.011	1.74 E−37	0.092	0.016	8.18 E−09
11p15.5	rs4980383	11	1,902,097	T/C	− 7.228	4.90 E−13	− 0.076	0.006	4.36 E−34	− 0.083	0.007	6.20 E−29	− 0.051	0.011	8.31 E−06
12p12.1	rs11836164	12	26,446,625	T/C	6.028	1.66 E−09	− 0.013	0.007	6.81 E−02	− 0.003	0.009	7.32 E−01	− 0.025	0.013	5.78 E−02
12p11.2	rs7297051	12	28,174,817	T/C	6.166	7.01 E−10	0.120	0.007	1.73 E−60	0.110	0.009	3.49 E−36	0.140	0.014	6.45 E−25
12q23.2	rs833472	12	103,005,100	T/C	7.134	9.76 E−13	0.078	0.020	7.59 E−05	0.085	0.024	3.04 E−04	0.049	0.037	1.80 E−01
19q13.33	rs1231281	19	49,239,200	A/G	6.860	6.87 E−12	− 0.008	0.006	2.30 E−01	− 0.010	0.008	2.07 E−01	− 0.002	0.012	8.72 E−01
20q13.13	rs17789629	20	48,892,374	A/C	− 5.478	4.30 E−08	− 0.011	0.008	1.32 E−01	− 0.001	0.009	9.48 E−01	− 0.034	0.014	1.44 E−02
22q13.1	rs34066050	22	38,612,604	A/G	− 5.534	3.14 E−08	− 0.034	0.006	5.58 E−08	− 0.042	0.008	2.35 E−08	− 0.010	0.011	3.78 E−01
22q13.2	rs6001939	22	40,892,794	T/C	8.791	1.48 E−18	− 0.117	0.010	1.31 E−32	− 0.106	0.012	1.20 E−19	− 0.115	0.018	8.77 E−11
*NDA*															
1p12	rs78395856	1	119,495,096	A/C	5.525	3.29 E−08	− 0.037	0.018	3.46 E−02	− 0.044	0.021	3.31 E−02	− 0.037	0.031	2.35 E−01
5q14.1	rs413472	5	80,930,992	T/C	5.551	2.84 E−08	0.004	0.006	5.49 E−01	0.010	0.008	2.11 E−01	− 0.012	0.012	3.18 E−01
8p11.23	rs16885613	8	36,848,357	T/C	− 12.788	1.92 E−37	− 0.076	0.008	1.58 E−20	− 0.070	0.010	5.06 E−13	− 0.099	0.015	2.38 E−11
10q21.2	rs2138555	10	64,220,494	A/G	5.900	3.63 E−09	− 0.044	0.006	1.48 E−12	− 0.047	0.008	3.89 E−10	− 0.030	0.011	8.58 E−03
12q22	rs61938093	12	96,026,737	T/C	− 6.266	3.72 E−10	0.089	0.007	1.34 E−38	0.091	0.008	5.53 E−28	0.069	0.013	3.39 E−08
22q13.2	rs73169097	22	41,027,870	T/C	6.012	1.83 E−09	− 0.120	0.010	1.98 E−34	− 0.110	0.012	5.34 E−21	− 0.117	0.018	4.33 E−11
*PMD*															
1q21.2	rs1868992	1	149,908,108	A/G	5.517	3.44 E−08	− 0.027	0.007	1.64 E−04	− 0.029	0.008	7.09 E−04	− 0.030	0.013	1.98 E−02
5q23.2	rs335189	5	122,446,856	C/G	6.196	5.78 E−10	0.027	0.007	8.38 E−05	0.026	0.008	1.46 E−03	0.012	0.012	3.32 E−01
5q31.1	rs76876329	5	131,237,759	T/C	5.530	3.21 E−08	0.012	0.009	1.87 E−01	0.007	0.011	5.11 E−01	0.014	0.017	3.99 E−01
5q33.3	rs11745230	5	158,171,008	T/G	− 5.793	6.90 E−09	− 0.064	0.008	1.30 E−14	− 0.065	0.010	3.47 E−11	− 0.068	0.015	6.72 E−06
5q35.1	rs2112670	5	169,557,594	A/G	− 5.598	2.17 E−08	− 0.013	0.006	4.71 E−02	− 0.023	0.008	2.83 E−03	0.012	0.012	3.03 E−01
6p22.3	rs3819405	6	16,399,557	T/C	5.806	6.42 E−09	0.040	0.007	1.25 E−08	0.045	0.008	5.38 E−08	0.027	0.013	3.45 E−02
6q25.1	rs4897107	6	149,601,591	T/C	6.842	7.81 E−12	0.036	0.008	5.98 E−06	0.049	0.010	3.55 E−07	0.018	0.015	2.15 E−01
6q25.1	rs9397436	6	151,952,002	A/G	7.303	2.81 E−13	0.184	0.012	2.00 E−54	0.138	0.014	1.71 E−22	0.272	0.021	4.01 E−40
8p11.23	rs10087804	8	36,858,140	C/G	6.282	3.35 E−10	− 0.077	0.008	8.34 E−21	− 0.071	0.010	3.64 E−13	− 0.098	0.015	6.55 E−11
10q21.2	rs10995187	10	64,273,026	A/G	10.424	1.92 E−25	0.129	0.009	2.85 E−49	0.134	0.011	1.74 E−37	0.092	0.016	8.18 E−09
11p15.5	rs4980383	11	1,902,097	T/C	− 5.860	4.62 E−09	− 0.076	0.006	4.36 E−34	− 0.083	0.007	6.20 E−29	− 0.051	0.011	8.31 E−06
12q23.2	rs61941038	12	102,989,316	A/T	− 6.477	9.37 E−11	− 0.075	0.020	1.47 E−04	− 0.085	0.024	3.07 E−04	− 0.032	0.037	3.86 E−01
15q26.1	rs4499190	15	94,275,057	G/C	− 5.654	1.57 E−08	− 0.001	0.007	8.90 E−01	0.001	0.009	8.71 E−01	− 0.008	0.013	5.69 E−01
16q12.2	rs11646715	16	53,824,007	A/G	7.183	6.84 E−13	0.046	0.006	6.89 E−13	0.042	0.008	4.14 E−08	0.055	0.012	1.60 E−06
19q13.33	rs12462111	19	49,171,306	T/C	5.847	5.02 E−09	− 0.017	0.007	1.03 E−02	− 0.015	0.008	5.32 E−02	− 0.011	0.012	3.51 E−01

^1^Genomic positions based on build GRCh37/hg19

^2^Coded as reference allele/effect allele

^3^Standard error

^4^Beta estimates, SEs, and *p* values of overall, ER-positive, and ER-negative breast cancer were based on the GWAS results published by the Breast Cancer Association Consortium (BCAC)

### Exploratory bioinformatic analysis

We estimated the phenotypic variance attributable to common variants, as previously described [26]. SNP heritability (*h*^2^_SNP_) was estimated as 0.32 (se = 0.04) for DA, 0.24 (se = 0.03) for NDA, and 0.27 (se = 0.03) for PMD. By partitioning the *h*^2^_SNP_ by 74 functional annotations [27], we observed that active enhancers marked by histone modification H3K27ac were enriched for all MD phenotypes (2.25-fold for DA, *p* = 7.10 × 10^−11^; 2.11-fold for NDA, *p* = 9.71 × 10^−7^ and 2.22-fold for PMD *p* = 7.71 × 10^−10^, Additional file 2: Table S9).

**Fig. 3:**
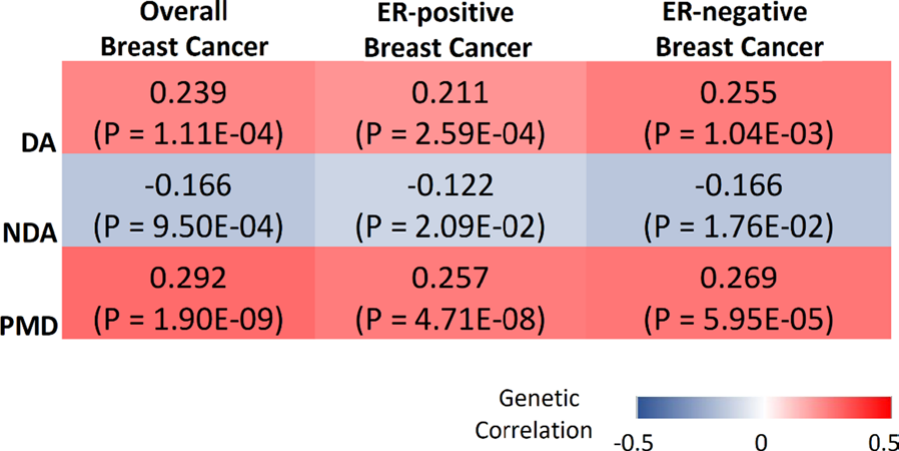
Genetic correlations between three MD phenotypes (DA, NDA, PMD) and breast cancer (overall, ER-positive, and ER-negative), estimated by LD score regression

We further quantified the genetic correlation between MD phenotypes and breast cancer risk (Fig. 3). DA and PMD showed positive genetic correlations with overall breast cancer (DA: *r*_g_ = 0.24, *p* = 1.11 × 10^−4^; PMD: *r*_g_ = 0.29, *p* = 1.90 × 10^−9^), ER-positive (DA: *r*_g_ = 0.21, *p* = 2.59 × 10^−4^; PMD: *r*_g_ = 0.26, *p* = 4.71 × 10^−8^), and ER-negative breast cancer (DA: *r*_g_ = 0.26, *p* = 1.04 × 10^−3^; PMD: *r*_g_ = 0.27, *p* = 5.95 × 10^−5^). In contrast, NDA showed a negative genetic correlation with breast cancer (overall: *r*_g_ =  − 0.17, *p* = 9.50 × 10^−4^; ER-positive: *r*_g_ =  − 0.12, *p* = 0.021; ER-negative: *r*_g_ = -0.17, *p* = 0.018).

We estimated the local genetic correlation between MD phenotypes and overall breast cancer by partitioning the genome into 1,703 independent blocks. In total, we identified nine significant pairwise local genetic correlations between MD phenotypes and overall breast cancer (DA: 6q25.1, 10q21.2, 11p15.5, 12p11.2, 22q13.2; NDA: 8p11.23; PMD: 5q33.3, 6q25.1, 10q21.2) (Additional file 1: Figure S9). All nine regions harbored at least one genome-wide significant locus for a MD phenotype and were directionally consistent with the breast cancer association.

### TWAS of MD phenotypes

Finally, we performed a TWAS investigating associations between the imputed expression of 7284 genes and MD phenotypes (Additional file 1: Figure S10, Additional file 2: Tables S10–S12), and identified significant associations with eight genes (Table 3). Six genes were either located in (*MTMR11*, *SMIM25*, and *TMEM184B*) or within the 1 Mb of the GWAS loci (*EP300, DES11,* and *MRPL23-AS1)*. The imputed expression of two additional genes was associated with MD phenotypes, including *SHOX2* (positively associated with NDA) and *CRISPLD2* (negatively associated with PMD). We replicated our TWAS findings using individual-level data for 24,158 women from an independent GWAS to impute expression for seven of the identified genes with available models in PredictDB [17, 33]. Five of the seven genes, except *EP300* and *DESI1*, were replicated at *p* < 0.05 with a consistent direction of effect (Additional file 2: Table S13).

**Table 3:** Genes with significant association between genetically predicted gene expression and MD phenotypes (DA, NDA, PMD), based on transcriptome-wide association study (TWAS)^1^

MD phenotype	Gene	CHR	Gene start position^2^	Gene end position^2^	Gene expression heritability	TWAS *Z* score	TWAS *p* value
DA	MTMR11	1	149,928,651	149,936,867	0.117	6.556	5.53 E−11
DA	EP300	22	41,091,786	41,180,079	0.059	5.074	3.89 E−07
DA	MRPL23-AS1	11	1,983,237	1,989,920	0.158	− 5.057	4.26 E−07
DA	SMIM25	20	50,267,486	50,279,795	0.198	− 4.893	9.92 E−07
DA	TMEM184B	22	38,219,291	38,273,034	0.112	4.864	1.15 E−06
DA	DESI1	22	41,598,028	41,621,096	0.139	4.779	1.76 E−06
NDA	SHOX2	3	158,096,011	158,106,503	0.155	5.027	4.98 E−07
PMD	MTMR11	1	149,928,651	149,936,867	0.117	5.517	3.45 E−08
PMD	CRISPLD2	16	84,819,984	84,920,768	0.177	− 4.832	1.35 E−06

^1^Statistical significance was defined as *p* < 0.05/(7,284*3) = 2.29 × 10^−6^

^2^Genomic positions based on build GRCh37/hg19

## Discussion

We conducted a GWAS for three MD phenotypes in 27,900 European ancestry women. We identified 28 distinct loci that were associated with at least one MD phenotype at genome-wide significance. Nine of these have not previously been reported to be associated with mammographic density. In addition, 14 of the 28 loci were also associated with breast cancer risk at genome-wide significance. We quantified the genetic correlation between MD phenotypes and breast cancer, further establishing the shared genetic basis between MD phenotypes and breast cancer risk. Finally, we conducted a TWAS and identified two additional novel associations between imputed expression level and MD phenotypes.

Previous GWAS based on data from MODE/BCAC identified 12 MD loci [12–16] and a recent GWAS of MD based on 24,192 women further discovered 31 novel loci [17]. In addition, GWAS investigating volumetric MD revealed one novel locus for percent dense volume (*HABP2* at 10q25.3) and two loci for absolute dense volume (*INHBB* at 2q14.2*, LINC01483* at 17q24.3) [36]. Previous studies support the association for DA lead SNP rs150249911, which is an intronic variant of the *ITGA1* gene at 5q11.2. *ITGA1*-coded integrin α1 protein upregulated following the expression of estrogen receptor β, a marker of breast cancer [37]. SNP rs413472 (5q14.1) is located in the *SSBP2* gene which has previously been implicated in breast cancer (*p* = 4.00 × 10^−5^) in Indonesian women [38]. DA lead SNP rs10155920 is in a long non-coding RNA located downstream of the *EGFR* gene at chromosome 7p11.2. *EGFR* is one of the most well-studied signaling pathways that contributes to the invasion, dissemination, and metastasis of breast tumors [39]. Breast cancer fine-mapping analysis has identified DA lead SNP rs7297051 as one of the four independent association signals of breast cancer at chromosome 12p11 [40], which is approximately 50 kb upstream of the *PTHLH* gene. *PTHLH* encodes parathyroid hormone-related protein (PTHrP), which aids in normal mammary gland development [41]. PTHrP has also been related to the prognosis of breast cancer, as its occasional secretion by tumor cells may promote osteoclastic activity and contribute to osteolytic bone metastases [42]. PMD lead SNP rs11646715 is in the *FTO* gene at chromosome 16q12.2 which encodes an mRNA demethylase and is well-known for its association with fat mass and obesity. Research has indicated that the *FTO* gene may play a role in cellular sensing of macronutrients and may be involved in the regulation of cell growth, which can at least partly explain its relationship with both obesity and breast cancer [43]. As we adjusted for BMI in our GWAS model, and further, rs11646715 was not associated with BMI in our data, the mechanisms underlying the associations between genetic variation in this region and MD likely differ from its effect on adiposity. Future studies are essential to elucidate potential biological mechanisms that link these genes to MD and ultimately breast cancer susceptibility. The novel DA locus at 8q24 has previously been associated with multiple types of cancer, including breast cancer [44]. To rule out that our finding was an artifact due to oversampling of breast cancer cases, we assessed the association with DA using controls only and observed a significant association (*p* = 5.64 × 10^−4^). Interestingly, rs58847541 is associated with breast cancer but in the opposite direction to the effect on DA.

Thirteen of the 28 GWAS loci were also associated with overall breast cancer risk with genome-wide significance, and had little difference in effect by cancer subtype. Among these, we observed multiple unexpected inconsistency in the direction of associations between MD-associated loci and breast cancer risk, including DA and PMD loci 1q21.2 and 2q14.2, DA loci 8q24.13 and 22q13.2, and NDA and PMD locus 8q11.23. The underlying biological mechanisms driving these discrepancies are unclear, but one potential explanation is that these loci may be involved in multiple pathways across life stages, which differentially affect breast development and the risk of breast cancer. Furthermore, the MD phenotypes we studied were radiologic reflection of the underlying breast tissue composition, which made it difficult to distinguish the epithelium from stroma tissue of the breast. We also investigated the association between 205 independent breast cancer SNPs and MD phenotypes and found that 45% of the variants were associated with at least one MD phenotype at *p* < 0.05. The local genetic correlation analysis in our study highlighted specific loci at which MD phenotypes and breast cancer showed evidence of shared heritability (DA: *ESR1, ZNF365, LSP1*, and *MKL1*; NDA: *8p11.23*; PMD: *ESR1* and *ZNF365*). These observations reinforce the strong shared genetic basis between mammographic density and breast cancer.

The SNP heritability (*h*^2^_SNP_), which can be interpreted as the proportion of phenotypic variance explained by the additive effects of all genotyped variants, was estimated to be 0.32 for DA, 0.24 for NDA, and 0.27 for PMD. Our estimates were slightly lower than those previously reported [17, 36], perhaps due to differences in the study populations or methodology [45]. Twin studies have estimated the heritability of the three MD phenotypes to all exceed 60% [9, 10]. The difference between the SNP-heritability estimates in our analysis and the estimates from twin studies may reflect the effect of rare variants not being genotyped and not in LD with any genotyped variants, or may be due to non-additive genetic effects, interactions between genetic variants and environmental factors, or uncontrolled shared environmental factors in the twin studies.

Our TWAS identified eight genes for which imputed expression levels were significantly associated with MD phenotypes. Six of these were either located in or close to the identified GWAS loci, suggesting the observed genotype–phenotype association may be mediated through gene expression. Two additional genes *SHOX2* and *CRISPLD2* were associated with NDA and PMD, respectively, and replicated in an independent study. Future studies are thus needed to elucidate the biological bases of these findings.

Our study has several strengths. It is the largest GWAS of mammographic density to date, enabling us to discover nine novel MD loci. We performed sensitivity analyses using controls, which reaffirmed that all significant associations were not spurious artifacts due to oversampling of cases. However, a few studies included in our study used the thresholding approach other than *CUMULUS*, which may cause inconsistency in the measurement of MD phenotypes and thus lead to biased results. Also, although previous studies have demonstrated that the MD measurement collected by *CUMULUS* was highly reproducible [8, 46], it is important to acknowledge that it was a reader-dependent approach and thus might inevitably be subjective to measurement error. Another weakness with our study is the lack of diversity, as our study sample only included women of European ancestry. Considering that the risk of breast cancer attributable to mammographic density may differ among racial/ethnic groups [47], future efforts should be made to collect mammogram and genotype data from racially diverse populations.

## Conclusion

In this study, we conducted a GWAS and TWAS of MD phenotypes using 27,900 women of European ancestry. Our study improved our understanding about the genetic background of MD phenotypes, and reinforced the evidence of their shared genetic basis with breast cancer risk.

## Supplementary Information

**Additional file 1.** Supplementary Information, Funding, Acknowledgement, and Supplementary Figures.

**Additional file 2.** Supplementary Tables.

## Data Availability

The datasets used and/or analyzed during the current study are available from the corresponding author on reasonable request.

## Abbreviations

BCAC: Breast Cancer Association Consortium
BMI: Body mass index
DA: Mammographic dense area
ER: Estrogen receptor
GWAS: Genome-wide association study
LD: Linkage disequilibrium
MAF: Minor allele frequency
MD: Mammographic density
NDA: Mammographic non-dense area
PMD: Percent mammographic density
SNP: Single nucleotide polymorphism
TWAS: Transcriptome-wide association study
