# Supplementary material for: Genome-wide and transcriptome-wide association studies of mammographic density phenotypes reveal novel loci
Source: Breast Cancer Res. 2022 Apr 12;24:27. doi: 10.1186/s13058-022-01524-0 (PMC9006574; doi:10.1186/s13058-022-01524-0)

**SUPLLEMENTARY INFORMATION**

**Introduction of participating studies and phenotype data collection.**

Information about BCAC can be found at <https://bcac.ccge.medschl.cam.ac.uk/bcac-groups/study-groups/>. The harmonization of genotype, phenotypes (mammographic density measurements) and covariates (age and BMI at mammogram collection) was performed at the BCAC data coordinating center incorporated in the University of Cambridge, in a larger effort that involved back and forth correspondence with studies individually. In the present study, we performed extra quality control process for the data provided by the BCAC data coordinating center and removed individuals with missing or unreasonable value for MD measures, age, or BMI.

***Studies with individual-level genotype and phenotype collected***

**ABCFS**

The Australian Breast Cancer Family Study (ABCFS) is a population-based, case–control-family study of breast cancer with an emphasis on early-onset disease, that was carried out in Melbourne and Sydney, Australia. Case patients were adult women living in the metropolitan areas of Melbourne and Sydney who were diagnosed with a histologically confirmed first primary cancer of the breast. Control subjects were adult women living in the metropolitan areas of Melbourne and Sydney and selected from the electoral roll by use of proportional random sampling based on the expected age distribution of the case patients (McCredie et al, 1998; Dite et al, 2003). For the sample included in the present study, genotyping was conducted using the iCOGS or OncoArray chip. Mammograms were digitized at a pixel size of 260 μm and a precision of 12 bits using an Array digitizer at the Australian Mammographic Density Research Facility at the University of Melbourne. The craniocaudal view was used for measuring mammographic density via the Cumulus software.

**BBCC**

The Bavarian Breast Cancer Cases and Controls Study (BBCC) is a case control study that aims at the identification of genetic and non-genetic risk factors for breast cancer (Heusinger et al., 2011, Rauh et al., 2012, Schrauder et al., 2008). Cases are hospital-based and controls are either hospital-based, part of a breast cancer screening program or recruited by a newspaper advertisement. Mammograms were obtained either at the time-point of the primary diagnosis of breast cancer (cases) or at time of study inclusion (controls). Participants had to be 18 years or older. Controls were not allowed to have any malignant disease and cases had to have a histologically proven invasive breast cancer. All participants completed an epidemiological questionnaire and provided a blood sample for germline DNA extraction. Genotyping was done with the iCOGS array. Cases were followed for further follow up. The measurement of mammographic density used the Madena software.

**BCEES**

The Breast Cancer Environment and Employment Study (BCEES) is a large, population-based case–control study of women aged between 18 and 80 years (Fritschi et al., 2013) recruited between May 2009 and January 2011. We obtained digitised mammographic images from craniocaudal film mammograms for 1903 women (668 cases and 1235 controls) who attended BreastScreen Western Australia. For breast cancer cases, prediagnosis mammograms were used. For controls, the earliest mammogram taken between 2009 and 2011 was used to match the years the cases were diagnosed. The Cumulus software (Sunnybrook Health Sciences Centre, Toronto, Canada) was used to estimate absolute dense area, percentage dense area and non-dense area. DNA was extracted from saliva samples and of the 1903 women with mammographic data available, 1033 women were genotyped using the Illumina OncoArray chip as part of the Breast Cancer Association Consortium (BCAC). The use of these data is approved by the University of Western Australia Human Research Ethics Committee (RA/4/1/7399) and Department of Health Western Australia Human Research Ethics Committee (RGS0000002843).

**BREOGAN**

The BREast Oncology GAlicia Network (BREOGAN) is a population-based study conducted since 1997 in two main cities in Galicia, Spain (Vigo and Santiago de Compostela), which cover approximately 700,000 inhabitants. The study currently includes 2,078 incident breast cancer cases diagnosed from 1997-2014 in two Galician hospitals with blood, tumour tissue and risk factor questionnaire, as well as clinical information, including pathology, survival and treatment variables. Controls were frequency-matched to cases according to 5-year age group, inclusion in the universal Galician Public Health Service (SERGAS) registry database, and place of residence. They are 2,282 healthy, unrelated female individuals from the same base population as cases randomly selected from SERGAS´ primary healthcare centers in the health areas of Santiago and Vigo. Over 2,000 cases and about 1,000 controls were genotyped with the Illumina OncoArray platform at the University of Cambridge, United Kingdom. For 623 of these women, we also had mammographic density measurements (381 breast cancer cases and 242 controls). We collected mammograms as close as possible to the date of blood collection. Mammograms were scanned and sent to Dr. Celine Vachon at the Mayo Clinic in Minnesota, where a single reader assessed mammographic density using Cumulus. These studies were approved by the Ethical Committee of Clinical Research in Galicia, Spain.

**CBCS**

The data examined originate from a population-based case-control study, the British Columbia (BC) study subpopulation in the Canadian Breast Cancer Study (CBCS). CBCS includes incident female breast cancer cases 40 to 80 years of age diagnosed between 2005 and 2009. Cases were recruited from the BC Cancer Registry. Controls were enrolled from the Screening Mammography Program from the same geographic area, and frequency matched to cases in 5-year age groups. Participation was 54% among cases and 57% amid controls. This study was restricted to postmenopausal participants: 606 cases and 595 controls. The final sample, determined by the availability of screening film mammograms, was comprised of 477 cases and 588 controls. A questionnaire was used to collect information about personal characteristics and medical history.DNA was extracted from blood or saliva samples. Genotyping was performed with a 768 SNP Golden Gate (Illumina) assay by the McGill University and Genome Quebec Innovation Centre, Montreal Canada. Quality control procedures for all SNPS and samples were previously described (Grundy et al., 2013).

Mammographic density measurement has been described (Velásquez García et al., 2018). The most recent normal mammogram before recruitment into CBCS was selected. For cases, the contralateral breast was selected; for controls, the side was chosen at random. Mammograms were digitized using the same device (iCAD TotalLook Mammo Advantage); the craniocaudal view was used in all instances. Total breast area and DA were determined using the interactive thresholding method with Cumulus software (Imaging Research Program, Sunnybrook Health Sciences Centre, University of Toronto, Toronto, Canada), by a blinded single reader (HAVG).

**FHRISK**

The FHRisk study was initiated in 2010 (Evans et al 2016). Over the following 2 years 2,000 women were recruited from a Family History Clinic (FHC) in Manchester with oversampling of previous breast cancers. Each woman had breast density measured by five methods (Astley et al., 2018) including visual assessment by two independent expert readers (radiologists, advanced practitioner radiographers and breast physicians) who marked the estimated percent density for each woman on visual analogue scales. The average density across radiographic views and both readers was taken. All women gave informed consent and completed a questionnaire to provide information on personal and lifestyle factors associated with risk of breast cancer. A blood DNA sample was obtained over sampling in those with breast cancer. An 18 Single Nucleotide Polymorphism Polygenic Risk Score was generated on 2,000 (Evans et al., 2017), but only 1400 have had the Illumina OncoArray.

**KCONFAB**

kConFab was initiated in 1997, and with the help of the Family Cancer Centres in Australia and New Zealand began enrolling families with a strong history of breast and breast/ovarian cancer. Genetic, epidemiological, medical and psychosocial data collected from these families by kConFab are stored in a de-identified fashion in a central relational database. Biospecimens collected from family members are used to characterise germ-line mutations in predisposing genes such as BRCA1, BRCA2, ATM, PALB2, p53, PTEN (Osborne et al., 2000). kConFab has accumulated data on more than 1,900 multigenerational, multi-case kindreds. Since this time, blood samples were collected from 9480 women, 5370 have a verified breast cancer. For 1,590 of these women. Mammograms from 433 women have been retrieved to calculate mammographic density data. These kConFab research study is approved by the Human Ethics Committee, Peter MacCallum Cancer Centre, Melbourne, Australia. The measurement of mammographic density used the Cumulus software.

**MCBCS**

The Mayo Clinic Breast Cancer study (MCBCS) is an Institutional Review Board-approved, ongoing clinic-based case-control study initiated in February 2001 at Mayo Clinic, Rochester, MN, USA. The study design has been presented previously (Easton et al., 2007, Cox et al., 2007). Clinic attendance formed the sampling frame for Mayo Clinic cases and controls. Consecutive cases were women aged 18 years or older with histologically confirmed primary invasive breast carcinoma who were recruited within 6 months of their date of diagnosis. Women with a history of cancer (excluding nonmelanoma skin cancer) were ineligible. Cases lived in the six-state region that defines Mayo Clinic's primary service population (Minnesota, Iowa, Wisconsin, Illinois, North Dakota and South Dakota). Control individuals without prior history of cancer (other than nonmelanoma skin cancer) were frequency matched on age (5-year age category), race and six-state region of residence to cases. Controls were recruited from the outpatient practice of the Divisions of General Internal Medicine and Primary Care Internal Medicine at Mayo Clinic, where they were seen for routine medical examinations. Written informed consent was obtained from all participants. Case participation was 69% and control participation was 71%. Available mammograms close to enrollment date were obtained on 824 cases and 868 matched controls, and breast density was estimated on the CC view (non-cancerous breast for cases and left side, for controls) using Cumulus. The present investigation consisted of genotyping performed by BCAC on the iCOGS and OncoArray arrays.

**MCCS**

The Melbourne Collaborative Cohort Study (MCCS), established in 1990–1994, includes 24,469 women living in Melbourne aged between 27 and 76 years at recruitment when information about lifestyle and demographic characteristics were obtained through administered structured questionnaires and weights and heights were measured. Participants were followed up from 1995–2002 by a postal questionnaire in which weights were self-reported and from 2003–2007 with face-to-face interviews when weights were measured. The Cancer Council Victoria's Human Research Ethics Committee approved the study protocol.

In 2009, we conducted a linkage between female MCCS participants and BreastScreen Victoria (BSV), the government-funded mammographic screening program. We identified 20,444 (84%) participants who had attended BSV at least once. These women were eligible for a nested case-control mammographic density study. We randomly sampled 884 women from the nested case–control study (182 cases, 702 controls). After excluding mammograms taken after the end of follow-up at December 31, 2007 (288 mammograms from 7 women) and those taken within 2 years before diagnosis of breast cancer (173 mammograms from 31 women), there were 3,954 mammograms from 846 women. Mammograms were digitized by the Australian Mammographic Density Research Facility at the University of Melbourne with an Array 2905 high-density film digitizer (Array Corporation Europe, Roden, the Netherlands). The digitised images were masked, and total breast area and DA were measured by using a semiautomated computer-assisted thresholding technique called Cumulus (Imaging Research Program, Sunnybrook Health Sciences Centre, University of Toronto, Toronto, Canada). NDA was calculated by subtracting DA from the total breast area. PMD was calculated by dividing DA by the total breast area. Images were measured by 2 independent readers blind to the disease status of participants. Mammograms were read in sets of approximately 100 images. A random sample of 10% of the images was repeated within each set to assess within-reader reliability. The sample of mammograms repeated in the first set was also repeated in every fifth set to assess within-reader reliability between sets.

**MEC**

The Multiethnic Cohort (MEC) Study is an ongoing prospective study of more than 215,000 participants aged 45-75 years from five ethnic groups (Japanese American, white, Latino, African American, and Native Hawaiian) recruited in Hawaii and Los Angeles, California (Kolonel et al., 2000). All participants completed a self-administered questionnaire at cohort entry (1993-1996). In 2001-2006, a biorepository subgroup consisting of participants who were within the catchment area for the University of Hawaii and University of Southern California were recruited to provide blood and urine samples (Park et al., 2009). A nested case-control study collected mammographic images for 607 cases and 667 controls and assessed breast density using a computer-assisted method (Cumulus) during 2001-2004 (Maskarinec et al., 2005). In a subset of 101 women with white ethnicity, mammographic density measures and genotyping information was available (Woolcott et al., 2009). The Institutional Review Boards at the University of Hawaii and the University of Southern California approved all study protocols.

**MMHS**

The MMHS is a prospective breast screening cohort that enrolled 19,924 patients having a screening mammogram from October 2003 through September 2006 at the Mayo Clinic in Rochester, MN. Women were invited to take part if they were at least 35 years old, residents of Minnesota, Iowa, or Wisconsin and had no personal history of breast cancer. Eligible women were mailed an invitation packet consisting of a consent form, a baseline questionnaire, request form to link to state tumor registries and access to any residual blood from clinical use. Of 38,883 eligible women, 19,924 provided written informed consented (51.2% adjusted response rate) and 12,811 had a blood samples were available. Genotyping was conducted by BCAC or Mayo Clinic using either the Illumina iCOGS or OncoArray platforms on a sample of premenopausal women, a nested case-control study (Vachon et al., 2015) and case-cohort (Vachon et al., 2019), for a combined total of 3210 women with GWAS and mammogram data. Breast density measures were estimated on both CC views from the enrollment mammogram (i.e. prediagnostic mammogram for cases) using Cumulus.

**NBCS**

This NBCS sub-study involves women attending the Norwegian national mammography screening program from 2002 to 2007 and biopsies were taken from breasts with varying mammographic density and newly diagnosed breast cancers (ethical approval S-02036). Blood was drawn from 121 healthy individuals (age 22-75) and 65 breast cancer patients (age 38-84) at the time of inclusion and information about mammographic density, pathological parameters of the tumors, hormone use and parity are available. Leukocyte DNA from 182 of the women was submitted to genotyping with the iCOGS array. The sample collection is described in publications, first in 2010 (Potapenko et al., 2010). The measurement of mammographic density used the Cumulus software.

**NC-BCFR**

The Northern California Breast Cancer Family Registry (NC-BCFR) was initiated in 1995 and enrolled ~4000 women with incident breast cancer and family members (John et al., 2019). Mammograms were collected for 579 women (sets of at least two sisters) for an ancillary genome-wide linkage study of mammographic density (Greenwood et al., 2011). Of these, 58 women of European ancestry were included in the present study. The cranio-caudal view in each mammogram was digitized and images were measured using a computer-assisted thresholding method. Cumulus software was used to set thresholds that defined the edge of the breast and outlined the areas of dense tissue. The pixels within these thresholds defined the total area of the breast and the area of dense tissue, from which percent density was calculated. The non-dense area of the mammogram was also calculated from these measurements. Genotype data were generated with the OncoArray SNP array. The study was approved by the IRB at the Cancer Prevention Institute of California and Stanford University.

**NHS&NHS2**

The Nurses’ Health Study (NHS) was initiated in 1976, when 121,700 US registered nurses aged 30 to 55 returned an initial questionnaire (Colditz et al., 2005). Blood samples were collected from 32,826 women and cheek cell samples were collected from and additional 29,684 women (Hankinson et al., 1998). The Nurses’ Health Study 2 (NHS2) was initiated in 1989 when 116, 430 US registered nurses aged 25 to 42 returned an initial questionnaire. Blood samples were collected from 29,612 women and cheek cell samples were collected from and additional 29,859 women (Tworoger et al., 2006). The genotype data for this study were generated as part of multiple initiatives (N=2,856; Lindström et al., 2017) as well as the OncoArray initiative (N=1,434). These studies were approved by the Committee on the Use of Human Subjects in Research at Brigham and Women’s Hospital. We collected mammograms as close as possible to the date of blood collection (1989 to 1990). To assess mammographic density, the craniocaudal (CC) views of both breasts were digitized at 261 μm/pixel with a Lumysis 85 laser film scanner, which covers a range of 0 to 4.0 optical density. We used the average percentage density of both breasts for this analysis. The measurement of mammographic density used the Cumulus software. This collection has been described in detail in a previous publication (Pettersson et al., 2011).

**OFBCR**

Breast cancer cases diagnosed between 1 Jan 1996-31 Dec 1998 were identified from the Ontario Cancer Registry which registers >97% of all cases residing in the province at the time of diagnosis. All women with invasive breast cancer aged 20–54 years who met the OFBCR definition for high genetic risk (family history of specific cancers particularly breast and ovarian, early onset disease, Ashkenazi ethnicity or a diagnosis of multiple breast cancer) were asked to participate by completing risk factor questionnaires and providing a blood sample. A 25% random sample of individuals in this age category who did not meet the OFBCR definition, 35% of those aged 55–69 at high risk and 8.75% aged 55–69 at low risk were also asked to participate. Individuals diagnosed in 2001 and 2002 were also included if they met high -risk criteria. Patients were genotyped with two different arrays: iCOGS and OncoArray as described previously (Amos et al., 2017).

We had mammographic density data obtained as described in Linton et al., 2013 and Byng et al., 1994. Original mammographic films were used and one craniocaudal mammogram view from all participants was selected, using the breast contralateral to the cancer. All mammograms were digitized using a Lumisys model 85 digitizer at a pixel size of 260 μm and 12 bits precision, and the digitized images were measured as described elsewhere. Using the Cumulus 3 program an observer first marked the outer and inner edges of the breast, from which total breast area was then calculated. Using a thresholding tool, the observer outlined the dense area. The percentage of total area that is dense, or PMD, and the non-dense area were calculated.

**PBCS**

The Polish Women’s Breast Cancer Study (PBCS) is a population-based case-control study conducted in two major cities in Poland (Warsaw and Łódź) during 2000-2003 (Garcia-Closas et al., 2006). PBCS cases were women aged 20-74 years with newly-diagnosed, pathologically-confirmed in situ or invasive breast carcinoma identified through a rapid identification system organized at five participating hospitals and via cancer registries. The current analysis was limited to breast cancer cases with available genotyping data and pre-treatment mammograms of the unaffected breast. Craniocaudal views were sent to the Ontario Cancer Institute in Toronto, Canada where they were digitized at a pixel size of 260 μm and a precision of 12 bits using a Lumisys 85 laser film scanner (Sun et al., 2013). The measurement of mammographic density used the Cumulus software. All participants provided written informed consent under a protocol approved by the U.S. National Cancer Institute and local (Polish) institutional review boards.

**PROCAS**

The PROCAS study was initiated in 2009 (Evans et al., 2016). Over the following 6 years 57,900 women were recruited at routine breast screening appointments in the UK NHS breast screening programme. Each woman had breast density measured by five methods (Astley et al., 2018) including visual assessment by two independent expert readers (radiologists, advanced practitioner radiographers and breast physicians) who marked the estimated percent density for each woman on visual analogue scales. The average density across radiographic views and both readers was taken. All women gave informed consent and completed a questionnaire to provide information on personal and lifestyle factors associated with risk of breast cancer. In a subset of 10,000 a saliva DNA sample was obtained over sampling in those with breast cancer. An 18 Single Nucleotide Polymorphism Polygenic Risk Score was generated on 10,000 (van Veen et al., 2018), but only 2,500 have had the Illumina OncoArray (Brentnall et al., 2020).

**SASBAC**

The Singapore and Sweden Breast Cancer Study (SASBAC) is a population-based case-control study. Incident cases from October 1993 to March 1995 identified via the 6 regional cancer registries in Sweden, to which reporting is mandatory. Controls were randomly selected from the total population registry in 5-year age groups to match the expected age-frequency distribution among cases. Patients and controls were recruited from Oct 1993 through April 1995 (Wedren et al., 2004). Breast cancer cases were genotyped using the Illumina HumanHap240 and Illumina HumanHap300 arrays. The controls were genotyped using the Illumina HumanHap550 array. The process of collecting mammographic density data in this study has been described previously (Tamimi et al., 2010). Film mammograms of the mediolateral oblique view were digitized using an Array 2905HD Laser Film Digitizer (Array Corporation, Tokyo, Japan), which covers a range of 0 to 4.7 optical density.1163 cases and 1378 controls were genotyped using iCOGs array, with 869 and 783 women respectively having MD data. The measurement of mammographic density used the Cumulus software.

**SIBS/EPIC**

The Sisters in Breast Screening Study (SIBS) was designed to study the genetic basis of quantitative phenotypes related to breast cancer, in particular breast density and sex-hormone levels [Varghese et al., 2012; Kataoka et al., 2009]. Women who attended the local Cambridge and Huntingdon Breast Screening Services to have mammographic examinations under the National Health Service (NHS) Breast Screening Program were identified and invited to participate in the study if they had 1 or more female blood relatives (sisters, half-sisters, first cousins, aunts, or nieces) who had also undergone mammographic screening. Women with relatives who could have screening within 2 years were also included. Families were also ascertained through newspaper and radio advertisements. Mammograms were undertaken as part of the UK NHS Breast Screening Program in which women, ages 50 to 64 years, are screened every 3 years by a 2-view (mediolateral and craniocaudal) mammogram. All available mammograms for each participant were retrieved from the local screening service and digitized using the Array 2905 Laser Film Digitizer and the program DICOM ScanPro Plus Version 1.3E (Array Corp), with 50-μm pixel resolution and 12-bit digitization, and an absorbance of 4.7. Density readings were conducted using a computer-assisted program, CUMULUS. The dense area, the nondense area, and hence the percentage density (dense area expressed as a percentage of the total breast area) were estimated for each film as continuous variables. Mediolateral views of both breasts were used as they were more widely available and breast density measured by the mediolateral and craniocaudal views are known to be highly correlated. All films were assessed by a single trained reader (J. Brown). Mammograms were analyzed in a random order. All readings were made independently for the right and the left breast. Ten precent of the films were rescored by the same reader for a different study and the intrareader repeatability was 0.84. Genome-wide genotyping was performed using the iCOGS array, with quality control as previously described [Michailidou et al., 2013].

EPIC-Norfolk is a population-based cohort study [Day et al., 1999]. The study recruited over 30,000 men and women aged 40-79 years at baseline between 1993 and 1998 from 35 participating general practices in Norfolk, East Anglia, UK. Mammograms for women in the screening age taken as part of the NHS Breast Screening Program were identified by linkage to the Norfolk and Norwich l Breast Screening service. Mammograms were retrieved, and scanning and scoring with Cumulus were carried out using the same protocol as for SIBS. Genotyping was performed using the iCOGS array.

**UKBGS**

In the Generations Study, serial mammograms were obtained from breast cancer cases and control subjects from breast cancer screening centres, which under the UK National Breast Cancer Screening Programme (UKBGS) invite women for routine 3-yearly screening from ages 50 to 70 (recently extended to 47 to 73) years. Analogue films were digitised at 84 microns per pixel with a VIDAR Diagnostic Pro Plus Scanner (optical density range 0-3.85). Digital images were transferred electronically. Mammograms of the mediolateral oblique (MLO) were selected for density reading. We selected mammograms closest to entry to the study, preferentially analogue images, but used digital images, where possible for both the case and control, if analogue images were not available. All selected mammograms were prediagnostic, except that, for a small minority of cases, in particular those diagnosed at the first screening visit, only the diagnostic mammograms were available. For these cases we selected the contralateral mammogram to avoid a potential bias that might have been otherwise introduced by only including cases diagnosed from a second or later screening visit. Cumulus software was used for determination of mammographic density1. All images were assessed by one observer, who was blinded to case-control status, and was trained by an experienced breast radiologist. The within-person intraclass correlation coefficient for percentage density was 0.94. Density readings were averaged between readings of the left and right breast, unless the mammogram was diagnostic, when only the contralateral reading was used.

***Studies with GWAS summary statistics collected***

**AMDTSS**

The Australian Mammographic Density Twins and Sisters Study (AMDTSS) was both a follow-up and expansion of the Australian Twins Study of Mammographic Density. Female twin pairs aged 40–70 years who participated in the original twin study between 1995 and 1999 were asked to participate further through the Australian Twin Registry. The study was also expanded to invite and include their non-twin sisters. Women were recruited from Western Australia, New South Wales, and Victoria for the original twin study, with women from Queensland and South Australia also included when the study was expanded between 2004 and 2008. Details can be found in Odefrey et al., 2010. All subjects provided informed consent for their samples to be used in genetic research and were selected based on extreme high and low values of absolute dense area by selecting from the upper and lower 10% of the residuals from regression analysis on body mass index and age. Ethics approval was obtained from the University of Melbourne, Melbourne. Only 1 woman from identical twin pairs was included. A total of 343 women were genotyped with the Illumina 610 Quad SNP chip at the Australian Genome Research Facility. Only original films were used, and all were digitized at a pixel size of 260 μm and a precision of 12 bits using an Array digitizer at the Australian Mammographic Density Research Facility at the University of Melbourne. One craniocaudal view of the right breast was used for each woman. The measurement of mammographic density used the Cumulus software.

**DDM-Spain**

Determinants of Mammographic Density in Spain (DDM-Spain) is a cross-sectional multicenter study that included 3584 women, aged 45-68 years, who attended breast cancer screening in seven specific screening centers within the Spanish Breast Cancer Screening Program network between October 2007 and July 2008. The design has been described elsewhere (Cabanes et al., 2011, Pollan et al., 2012). After signing the informed consent, women were interviewed at the screening center by purpose-trained interviewers who also performed an anthropometric examination of the participants following standardized procedures and using the same tools. Participants also donate a saliva sample that was stored using the Oragene DNA Collection Kit. Mammographic density was assessed in the craniocaudal view of the left breast by a single radiologist with high intra-observer consistency22 using a semi-quantitative scale with 6 categories (0%; <10%; 10%-25%; 25%-50%; 50%-75% and >75%). For massive genotyping 239 pairs of women with extreme density phenotypes (low/high) were selected, matching these women by screening center, age (+/- 2 years), menopausal status and BMI (+/- 2 kg m-2). DNA samples were gentyped with the Illumina Human610-Quad BeadChip platform (Illumina, SanDiego, CA). For the present study, the selection was restricted to those screening centers that used analogic devices (four out of seven), giving a total of 270 women (135 pairs) with high (>50%) versus low (<10%) mammographic density, according to the semi-quantitative scale originally used. Their mammograms were scanned and sent to the London School of Public Health, where a single reader (Isabel dos-Santos-Silva) assessed mammographic density using Cumulus.

**OOA**

This is family-based study of women from the Old Order Amish (OOA) population of Lancaster County, Pennsylvania. Between May of 2005 and December of 2009, we recruited 1,521 women, with the primary goal of identifying genetic factors that influence mammographic density. All women were between the ages of 40 and 88 years (mean of 54 years). Additional study design details, including eligibility criteria, are described elsewhere (Douglas et al., 2008, dbGaP accession pha003604.1). All women gave written, informed consent, and institutional review boards at the Universities of Michigan and Maryland approved all aspects of the protocol. We screened 1,458 women via two-view film screen mammography, and for women who had had a mammogram in the previous 12 months (n=23), we requested medical record release of their most recent mammogram. We digitized at least one craniocaudal (CC) view from each woman with a LUMISYS 85 laser scanner at a pixel size of 50 μm x 50 μm and 4,096 gray levels. We measured total breast area and absolute dense area from a digitized CC view of the right or left breast using interactive thresholding and our computer-assisted program Mammographic Density ESTimator (MDEST). The Center for Inherited Disease Research genotyped 2,443,179 SNPs using the Illumina HumanOmni2.5-4v1_B Array. GWA results are based on 1,472 women, including 723 pre- and 749 post-menopausal women. Summary-level data and analysis details and methods are available through dbGaP (accession number pha003604.1). We imputed genotypes using (1) CEU data from HapMap Phase II (release 22), (2) the subset of genotyped SNPs that passed our quality control filters and were in HapMap, and (3) a hidden Markov model-based algorithm as implemented in MaCH 1.0.1738,39. We excluded SNPs with minor allele frequencies less than 1% in the OOA sample or estimated MaCH RSQ below 0.3. We conducted genome-wide association analyses of dense and non-dense areas of the breast and the ratio of dense to total breast area using the efficient mixed-model association expected (EMMAX) program. We tested for trait-SNP associations under an additive genetic model with imputed dosages. Prior to analysis, all three traits were square root transformed and adjusted for age, body mass index, and menopausal status.

**MCOCS**

The Mayo Clinic Ovarian Case Control Study (MCOCS) is a case-control study of ovarian cancer conducted at the Mayo Clinic. The details of the design of this study have been previously described (Goode et al., 2010, Sellers et al., 2005). Briefly, ovarian cancer cases seen at the Mayo Clinic from 2000-2007 and residing in a six-state surrounding region were recruited. Controls were identified from women with at least one intact ovary who were undergoing outpatient general medical examinations and were frequency-matched to cases on age and state of residence. All controls that had a screening mammogram performed at the Mayo Clinic within five years prior to enrollment and GWAS information were eligible for the current study. The craniocaudal mammogram of the left breast was digitized on the Array 2905HD Laser Film Digitizer (Array Corporation, Tokyo, Japan), which covers a range of 0 to 4.7 optical density. The measurement of mammographic density used the Cumulus software. The protocol was approved by the Mayo Clinic Institutional Review Board.

**Mayo-VTE**

The Mayo Venous Thromboembolism Study **(**Mayo VTE) is a case-control study of venous thromboembolism (VTE) conducted at the Mayo Clinic (Rochester, MN)). The details of the design of this study have been previously described (Heit et al., 2011). Briefly, cases were consecutive Mayo Clinic outpatients ages 18 years or older with objectively-diagnosed deep vein thrombosis or pulmonary embolism who resided in the upper Midwest United States and who were referred to the Mayo Clinic Special Coagulation Laboratory or Thrombophilia Center over the study period, 1994–2009. Clinic-based controls were prospectively selected from persons undergoing outpatient general medical examinations in 2004–2009 who had no previous diagnosis of VTE or superficial vein thrombosis and were frequency matched on the age group (20–29, 30–39, 40–49, 50–59, 60–69, 70–79 years), sex, state of residence and myocardial infarction (MI)/stroke status distribution of the cases. Potential controls with active cancer, antiphospholipid antibody syndrome, rheumatologic or other autoimmune disorder, or prior bone marrow or liver transplant, were excluded. The protocol was approved by the Mayo Clinic Institutional Review Board. Mammograms of the left breast performed within five years prior to enrollment date were obtained and digitized on the Array 2905HD Laser Film Digitizer (Array Corporation, Tokyo, Japan), which covers a range of 0 to 4.7 optical density. Cases were not used in the analyses but 317 of the genotyped controls had complete mammographic density and covariate information. The measurement of mammographic density used the Cumulus software.

**MBCFS**

The families included in Minnesota Breast Cancer Family Study have been described earlier (Sellers et al., 1999, Vachon et al., 2007). In total, 426 multigenerational families were ascertained through a breast cancer proband diagnosed from 1944 to 1952 at the University of Minnesota. Probands were consecutive cases, unselected for family history. First- and second-degree blood relatives of the proband and spouses were interviewed between 1990 and 1996; 93% of those contacted provided a telephone interview that included detailed risk factor information. Almost all (99%) women in the 426 families were Caucasian and from Minnesota. Simulation studies were done to identify the families most informative for linkage analyses. A subset of 90 of the 426 families was selected, and 1,146 family members were invited to provide a blood or buccal sample as a source of DNA; 901 (79%) consented. After the exclusion of 12 individuals due to Mendelian (familial) inconsistencies across markers, the final sample included 89 families, with 889 Caucasian individuals (133 men, 756 women). As part of the parent study, women provided the location of the most recent mammogram and permission to obtain and digitize their mammograms. Mammograms were requested from clinics across the United States, and all were recent mammograms done over the 1990 to 2001 period when national standards were in place for mammography. Among the 737 age-eligible women, we retrieved the mammograms of 658 (89%). Of women with mammograms, 618 (82%) had both craniocaudal and mediolateral oblique views available. Five percent of women had a breast cancer diagnosis during the follow-up period (2000–2002); for these women, mammograms before the diagnosis were used. For this study, a total of 597 women with remaining DNA were genotyped with the Illumina HumanHap 660W Quad array. The protocol was approved by the Mayo Clinic Institutional Review Board. Original mammograms were obtained on 658 women and digitized on a Lumiscan 75 scanner with 12-bit grayscale depth. The pixel size was 0.130x0.130 mm^2^ for both the 18x24- and 24x30-cm^2^ films. For this study, mammographic density from the mediolateral oblique and craniocaudal views were averaged and used as the phenotype. The measurement of mammographic density used the Cumulus software.

**NHS**

The Nurses' Health Study (NHS) was initiated in 1976, when 121,700 US registered nurses aged 30 to 55 returned an initial questionnaire (Colditz et al., 2005). During 1989 and 1990, blood samples were collected from 32,826 women (Hankinson et al., 1998). As part of the Cancer Genetic Markers of Susceptibility Project (CGEMS) breast cancer GWAS, 1,145 breast cancer cases and 1,142 controls were genotyped with the Illumina HumanHap500. For 1,590 of these women - of which 806 were breast cancer cases and 784 were controls - we also had mammographic density measurements. Additional 778 women with mammographic density data were genotyped with the Illumina OmniExpress Array at the Broad Institute. These studies were approved by the Committee on the Use of Human Subjects in Research at Brigham and Women's Hospital. We collected mammograms as close as possible to the date of blood collection (1989 to 1990). To assess mammographic density, the craniocaudal (CC) views of both breasts were digitized at 261 μm/pixel with a Lumysis 85 laser film scanner, which covers a range of 0 to 4.0 optical density. We used the average percentage density of both breasts for this analysis. The measurement of mammographic density used the Cumulus software. This collection has been described in detail in a previous publication (Pettersson et al., 2011).

**SASBAC**

The Singapore and Sweden Breast Cancer Study (SASBAC) is a population-based case-control study of postmenopausal breast cancer in women born in Sweden aged 50 to 74 years at the time of enrollment, which was between 1 October 1993 and 31 March 1995. Controls were randomly selected from the Swedish Total Population Register and were frequency matched to the expected age distribution of the cases. Details on data collection and subjects have been described previously (Wedren et al., 2004). The final study group with both mammographic density and genotype data included 571 breast cancer cases and 742 controls. Approval of the study was given by the ethical review board at the Karolinska Institutet (Stockholm, Sweden) and six region-specific ethical review boards where the subjects were based. Informed consent was obtained from each participant. Cases and controls were genotyped separately and were therefore treated as two separate populations in this analysis. Breast cancer cases were genotyped using the Illumina HumanHap240 and Illumina HumanHap300 arrays. The controls were genotyped using the Illumina HumanHap550 array. The process of collecting mammographic density data in this study has been described previously (Tamimi et al., 2010). Film mammograms of the mediolateral oblique view were digitized using an Array 2905HD Laser Film Digitizer (Array Corporation, Tokyo, Japan), which covers a range of 0 to 4.7 optical density. For controls, the breast side was randomized. For cases, the side contralateral to the tumor was used. The density resolution was set at 12-bit spatial resolution. The measurement of mammographic density used the Cumulus software.

**SIBS**

We used data from the Sisters in Breast Screening study (SIBS), an ongoing study designed to map genes associated with breast density (Kataoka et al.,2009). Families were identified through the National Health Service breast screening program in the United Kingdom. Eligibility was restricted to families in which two or more female blood relatives (sisters, half-sisters, first cousins, or aunt-niece) had had mammographic screening. Families whose member could have screening within 2 years of the recruitment were also included. The study was approved by the local research ethical committee. Study recruitment commenced in October 2002. The current analysis was limited to families whose data including mammographic density measurements were completed by July 2007. The measurement of mammographic density used the Cumulus software.

**Details of software used in this study**

**Cumulus**

Cumulus is a semi-automated way to quantitatively measure the mammographic density, based on the interactive segmentation of digitized film mammograms by a radiologist or trained image interpreter[1]. The observers viewed the digitalized mammograms first and selected grey-level thresholds from which the breast and regions of dense tissue in the breast were identified. The number of pixels had a light appearance were counted and used to calculate the area of mammographic dense tissue. The number of pixels had a dark appearance, which were calculated by the subtracting the number of pixels in light area from the whole breast, were used to calculate the area of mammographic non-dense tissue. The percent mammographic density can then be calculated as the percent of pixels had a light appearance. Cumulus is an approach that can minimize the measurement error, as both the inter-rater [2] and intra-rater [3] reproducibility has been demonstrated.

**METAL**

METAL is a tool for meta-analysis genome-wide association scans. METAL can combine either 1) test statistics and standard errors or 2) p-values across studies (taking sample size and direction of effect into account). METAL analysis is a convenient alternative to a direct analysis of merged data from multiple studies. It is especially appropriate when data from the individual studies cannot be analyzed together because of differences in ethnicity, phenotype distribution, gender or constraints in sharing of individual level data imposed. Meta-analysis results in little or no loss of efficiency compared to analysis of a combined dataset including data from all individual studies. More information can be found at https://genome.sph.umich.edu/wiki/METAL_Documentation, or Miller et al., Bioinformatics, 2010 [4].

**LocusZoom**

LocusZoom is a tool that can visually displays regional information such as the strength and extent of the association signal relative to genomic position, local linkage disequilibrium (LD) and recombination patterns and the positions of genes in the region. The software utilizes LD information from HapMap Phase II (CEU, YRI and JPT+CHB) or 1000 Genomes (CEU) and gene information from the UCSC browser, and will accept SNP identifiers in dbSNP or 1000 Genomes format. Single plots are generated in ∼20 s. Source code and associated databases are available for download and local installation, and full documentation is available online. More information can be found at http://locuszoom.org/, or Pruim et al., Bioinformatics, 2010 [5].

**COJO-GCTA**

GCTA (Genome-wide Complex Trait Analysis) is a software package initially developed to estimate the proportion of phenotypic variance explained by all genome-wide SNPs for a complex trait but has been greatly extended for many other analyses of data from genome-wide association studies (GWASs). COJO: conditional & joint association analysis using GWAS summary statistics is currently implemented in the GCTA package. More information can be found at https://yanglab.westlake.edu.cn/software/gcta/#COJO, or Yang et al., Nature Genetics, 2012 [6].

**LDSC**

LDSC (LD score regression) is a command line tool for estimating heritability and genetic correlation from GWAS summary statistics. Both polygenicity (many small genetic effects) and confounding biases, such as cryptic relatedness and population stratification, can yield an inflated distribution of test statistics in genome-wide association studies. LD Score regression can quantify the contribution of each by examining the relationship between test statistics and linkage disequilibrium. The LD Score regression intercept can be used to estimate a more powerful and accurate correction factor than genomic control. More information can be found at https://github.com/bulik/ldsc; or Bulik-Sullivan et al., Nature Genetics, 2015 [7].

**ρHESS**

HESS (Heritability Estimation from Summary Statistics) is a software package for estimating and visualizing local SNP-heritability and genetic covariance (correlation) from GWAS summary association data. The method estimates the total trait variance explained by the typed variants at a single locus in the genome (local SNP heritability) from genome-wide association study (GWAS) summary data while accounting for linkage disequilibrium among variants. The method was built upon recent works that have treated causal effect sizes as fixed effects and model the genotypes at the locus as random correlated variables. The estimator can be viewed as a weighted summation of the squares of the projection of GWAS effect sizes onto the eigenvectors of the LD matrix at the considered locus, where the weights are inversely proportional to the corresponding eigenvalues. Through extensive simulations, HESS was shown to be unbiased when in-sample LD was available, regardless of disease architecture (i.e., the number of causals and distribution of effect sizes). More information can be found at https://huwenboshi.github.io/hess/#hess or Shi et al., American Journal of Human Genetics, 2016 [8].

**FUSION**

FUSION is a suite of tools for performing a transcriptome-wide (or any other ome-wide) association study by predicting functional/molecular phenotypes into GWAS using only summary statistics. The goal is to identify associations between a GWAS phenotype and a functional phenotype that was only measured in reference data. FUSION can leverage a relatively small set of reference individuals for whom both gene expression and genetic variation (single nucleotide polymorphisms, SNPs) have been measured to impute the cis-genetic component of expression into a much larger set of phenotyped individuals from their SNP genotype data. The imputed expression can be viewed as a linear model of genotypes with weights based on the correlation between SNPs and gene expression in the training data while accounting for linkage disequilibrium among SNPs. FUSION then correlates the imputed gene expression to the trait to perform a transcriptome-wide association study and identify significant expression-trait associations. More information can be found at http://gusevlab.org/projects/fusion/ or Gusev et al., Nature Genetics, 2016 [9].

**References:**

1. Byng JW, Boyd NF, Fishell E, Jong RA, Yaffe MJ: **The quantitative analysis of mammographic densities**. *Phys Med Biol* 1994, **39**(10):1629-1638.

2. Rice MS, Rosner BA, Tamimi RM: **Percent mammographic density prediction: development of a model in the nurses' health studies**. *Cancer Causes Control* 2017, **28**(7):677-684.

3. Pettersson A, Hankinson SE, Willett WC, Lagiou P, Trichopoulos D, Tamimi RM: **Nondense mammographic area and risk of breast cancer**. *Breast Cancer Res* 2011, **13**(5):R100.

4. Willer CJ, Li Y, Abecasis GR: **METAL: fast and efficient meta-analysis of genomewide association scans**. *Bioinformatics* 2010, **26**(17):2190-2191.

5. Pruim RJ, Welch RP, Sanna S, Teslovich TM, Chines PS, Gliedt TP, Boehnke M, Abecasis GR, Willer CJ: **LocusZoom: regional visualization of genome-wide association scan results**. *Bioinformatics* 2010, **26**(18):2336-2337.

6. Yang J, Ferreira T, Morris AP, Medland SE, Genetic Investigation of ATC, Replication DIG, Meta-analysis C, Madden PA, Heath AC, Martin NG *et al*: **Conditional and joint multiple-SNP analysis of GWAS summary statistics identifies additional variants influencing complex traits**. *Nat Genet* 2012, **44**(4):369-375, S361-363.

7. Bulik-Sullivan BK, Loh PR, Finucane HK, Ripke S, Yang J, Schizophrenia Working Group of the Psychiatric Genomics C, Patterson N, Daly MJ, Price AL, Neale BM: **LD Score regression distinguishes confounding from polygenicity in genome-wide association studies**. *Nat Genet* 2015, **47**(3):291-295.

8. Shi H, Kichaev G, Pasaniuc B: **Contrasting the Genetic Architecture of 30 Complex Traits from Summary Association Data**. *Am J Hum Genet* 2016, **99**(1):139-153.

9. Gusev A, Ko A, Shi H, Bhatia G, Chung W, Penninx BW, Jansen R, de Geus EJ, Boomsma DI, Wright FA *et al*: **Integrative approaches for large-scale transcriptome-wide association studies**. *Nat Genet* 2016, **48**(3):245-252.

**FUNDING**

This work was supported by CA244670 and CA194393.

BCAC is funded by the European Union's Horizon 2020 Research and Innovation Programme (grant numbers 634935 and 633784 for BRIDGES and B-CAST respectively), and the PERSPECTIVE I&I project, funded by the Government of Canada through Genome Canada and the Canadian Institutes of Health Research, the Ministère de l’Économie et de l'Innovation du Québec through Genome Québec, the Quebec Breast Cancer Foundation. The EU Horizon 2020 Research and Innovation Programme funding source had no role in study design, data collection, data analysis, data interpretation or writing of the report. Additional funding for BCAC is provided via the Confluence project which is funded with intramural funds from the National Cancer Institute Intramural Research Program, National Institutes of Health.

Genotyping of the OncoArray was funded by the NIH Grant U19 CA148065, and Cancer UK Grant C1287/A16563 and the PERSPECTIVE project supported by the Government of Canada through Genome Canada and the Canadian Institutes of Health Research (grant GPH-129344) and, the Ministère de l’Économie, Science et Innovation du Québec through Genome Québec and the PSRSIIRI-701 grant, and the Quebec Breast Cancer Foundation. Funding for iCOGS came from: the European Community's Seventh Framework Programme under grant agreement n° 223175 (HEALTH-F2-2009-223175) (COGS), Cancer Research UK (C1287/A10118, C1287/A10710, C12292/A11174, C1281/A12014, C5047/A8384, C5047/A15007, C5047/A10692, C8197/A16565), the National Institutes of Health (CA128978) and Post-Cancer GWAS initiative (1U19 CA148537, 1U19 CA148065 and 1U19 CA148112 - the GAME-ON initiative), the Department of Defence (W81XWH-10-1-0341), the Canadian Institutes of Health Research (CIHR) for the CIHR Team in Familial Risks of Breast Cancer, and Komen Foundation for the Cure, the Breast Cancer Research Foundation, and the Ovarian Cancer Research Fund.

The Australian Breast Cancer Family Study (**ABCFS**) was supported by grant UM1 CA164920 from the National Cancer Institute (USA). The content of this manuscript does not necessarily reflect the views or policies of the National Cancer Institute or any of the collaborating centers in the Breast Cancer Family Registry (BCFR), nor does mention of trade names, commercial products, or organizations imply endorsement by the USA Government or the BCFR. The ABCFS was also supported by the National Health and Medical Research Council of Australia, the New South Wales Cancer Council, the Victorian Health Promotion Foundation (Australia) and the Victorian Breast Cancer Research Consortium. J.L.H. is a National Health and Medical Research Council (NHMRC) Senior Principal Research Fellow. M.C.S. is a NHMRC Senior Research Fellow.

The Australian Mammographic Density Twins and Sisters Study (**AMDTSS**) was supported by the National Breast Cancer Foundation, Cancer Australia, the National Health and Medical Research Council of Australia, and facilitated by Twins Research Australia.

The work of the **BBCC** was partly funded by ELAN-Fond of the University Hospital of Erlangen.

The **BCEES** was funded by the National Health and Medical Research Council, Australia and the Cancer Council Western Australia and acknowledges funding from the National Breast Cancer Foundation (JS).

The BREast Oncology GAlician Network (**BREOGAN**) is funded by Acción Estratégica de Salud del Instituto de Salud Carlos III FIS PI12/02125/Cofinanciado FEDER; Acción Estratégica de Salud del Instituto de Salud Carlos III FIS Intrasalud (PI13/01136); Programa Grupos Emergentes, Cancer Genetics Unit, Instituto de Investigacion Biomedica Galicia Sur. Xerencia de Xestion Integrada de Vigo-SERGAS, Instituto de Salud Carlos III, Spain; Grant 10CSA012E, Consellería de Industria Programa Sectorial de Investigación Aplicada, PEME I + D e I + D Suma del Plan Gallego de Investigación, Desarrollo e Innovación Tecnológica de la Consellería de Industria de la Xunta de Galicia, Spain; Grant EC11-192. Fomento de la Investigación Clínica Independiente, Ministerio de Sanidad, Servicios Sociales e Igualdad, Spain; and Grant FEDER-Innterconecta. Ministerio de Economia y Competitividad, Xunta de Galicia, Spain.

**CBCS** is funded by the Canadian Cancer Society (grant # 313404) and the Canadian Institutes of Health Research.

**FHRISK** and **PROCAS** are funded from NIHR grant PGfAR 0707-10031. DGE, AH and WGN are supported by the NIHR Manchester Biomedical Research Centre (IS-BRC-1215-20007).

**kConFab** is supported by a grant from the National Breast Cancer Foundation, and previously by the National Health and Medical Research Council (NHMRC), the Queensland Cancer Fund, the Cancer Councils of New South Wales, Victoria, Tasmania and South Australia, and the Cancer Foundation of Western Australia. Financial support for the AOCS was provided by the United States Army Medical Research and Materiel Command [DAMD17-01-1-0729], Cancer Council Victoria, Queensland Cancer Fund, Cancer Council New South Wales, Cancer Council South Australia, The Cancer Foundation of Western Australia, Cancer Council Tasmania and the National Health and Medical Research Council of Australia (NHMRC; 400413, 400281, 199600). G.C.T. and P.W. are supported by the NHMRC. RB was a Cancer Institute NSW Clinical Research Fellow. LAMBDA

The **MCBCS** was supported by the NIH grants R35CA253187, R01CA192393, R01CA116167, R01CA176785 a NIH Specialized Program of Research Excellence (SPORE) in Breast Cancer [P50CA116201], and the Breast Cancer Research Foundation.

Melbourne Collaborative Cohort Study (MCCS) cohort recruitment was funded by VicHealth and Cancer Council Victoria. The MCCS was further augmented by Australian National Health and Medical Research Council grants 209057, 396414 and 1074383 and by infrastructure provided by Cancer Council Victoria. Cases and their vital status were ascertained through the Victorian Cancer Registry and the Australian Institute of Health and Welfare, including the National Death Index and the Australian Cancer Database.

The **MEC** was supported by NIH grants CA63464, CA54281, CA098758, CA132839 and CA164973.

The **MMHS** study was supported by NIH grants CA97396, CA128931, CA116201, CA140286 and CA177150.

The **NBCS** has received funding from the K.G. Jebsen Centre for Breast Cancer Research; the Research Council of Norway grant 193387/V50 (to A-L Børresen-Dale and V.N. Kristensen) and grant 193387/H10 (to A-L Børresen-Dale and V.N. Kristensen), South Eastern Norway Health Authority (grant 39346 to A-L Børresen-Dale) and the Norwegian Cancer Society (to A-L Børresen-Dale and V.N. Kristensen).

The Northern California Breast Cancer Family Registry (**NC-BCFR**) and Ontario Familial Breast Cancer Registry (**OFBCR**) were supported by grant U01CA164920 from the USA National Cancer Institute of the National Institutes of Health. The content of this manuscript does not necessarily reflect the views or policies of the National Cancer Institute or any of the collaborating centers in the Breast Cancer Family Registry (BCFR), nor does mention of trade names, commercial products, or organizations imply endorsement by the USA Government or the BCFR.

The **NHS** was supported by NIH grants P01 CA87969, UM1 CA186107, and U19 CA148065.

The **NHS2** was supported by NIH grants UM1 CA176726 and U19 CA148065.

The **PBCS** was funded by the Intramural Research Program of the National Cancer Institute, Department of Health and Human Services, USA.

The **SASBAC** study was supported by funding from the Agency for Science, Technology and Research of Singapore (A*STAR), the US National Institute of Health (NIH) and the Susan G. Komen Breast Cancer Foundation.

**UKBGS** is funded by Breast Cancer Now and the Institute of Cancer Research (ICR), London. ICR acknowledges NHS funding to the NIHR Biomedical Research Centre.

The replication of GWAS and TWAS results is supported by NIH grants R01CA166827, R01CA168893, and R01CA237541.

**ACKNOWLEDGEMENT**

We thank all the individuals who took part in these studies and all the researchers, clinicians, technicians and administrative staff who have enabled this work to be carried out.

**ABCFS** thank Maggie Angelakos, Judi Maskiell, Gillian Dite.

**BBCS** thanks Eileen Williams, Elaine Ryder-Mills, Kara Sargus.

**BCEES** thanks Allyson Thomson, Christobel Saunders, Terry Slevin, BreastScreen Western Australia, Elizabeth Wylie, Rachel Lloyd.

The **BREOGAN** study would not have been possible without the contributions of the following: Manuela Gago-Dominguez, Jose Esteban Castelao, Angel Carracedo, Victor Muñoz Garzón, Alejandro Novo Domínguez, Maria Elena Martinez, Sara Miranda Ponte, Carmen Redondo Marey, Maite Peña Fernández, Manuel Enguix Castelo, Maria Torres, Manuel Calaza (BREOGAN), José Antúnez, Máximo Fraga and the staff of the Department of Pathology and Biobank of the University Hospital Complex of Santiago-CHUS, Instituto de Investigación Sanitaria de Santiago, IDIS, Xerencia de Xestion Integrada de Santiago-SERGAS; Joaquín González-Carreró and the staff of the Department of Pathology and Biobank of University Hospital Complex of Vigo, Instituto de Investigacion Biomedica Galicia Sur, SERGAS, Vigo, Spain.

**CBCS** thanks study participants, co-investigators, collaborators and staff of the Canadian Breast Cancer Study, and project coordinators Agnes Lai and Celine Morissette.

**FHRISK** and **PROCAS** thank NIHR for funding. **GC-HBOC** thanks Stefanie Engert, Heide Hellebrand, Sandra Kröber and LIFE - Leipzig Research Centre for Civilization Diseases (Markus Loeffler, Joachim Thiery, Matthias Nüchter, Ronny Baber).

**kConFab** wish to thank Heather Thorne, Eveline Niedermayr, all the kConFab research nurses and staff, the heads and staff of the Family Cancer Clinics, and the Clinical Follow Up Study (which has received funding from the NHMRC, the National Breast Cancer Foundation, Cancer Australia, and the National Institute of Health (USA)) for their contributions to this resource, and the many families who contribute to kConFab.

The **MCCS** was made possible by the contribution of many people, including the original investigators, the teams that recruited the participants and continue working on follow-up, and the many thousands of Melbourne residents who continue to participate in the study.

We thank the coordinators, the research staff and especially the **MMHS** participants for their continued collaboration on research studies in breast cancer.

The following are **NBCS** Collaborators: Kristine K. Sahlberg (PhD), Anne-Lise Børresen-Dale (Prof. Em.), Lars Ottestad (MD), Rolf Kåresen (Prof. Em.) Dr. Ellen Schlichting (MD), Marit Muri Holmen (MD), Toril Sauer (MD), Vilde Haakensen (MD), Olav Engebråten (MD), Bjørn Naume (MD), Alexander Fosså (MD), Cecile E. Kiserud (MD), Kristin V. Reinertsen (MD), Åslaug Helland (MD), Margit Riis (MD), Jürgen Geisler (MD), OSBREAC and Grethe I. Grenaker Alnæs (MSc).

For **NHS** and **NHS2** the study protocol was approved by the institutional review boards of the Brigham and Women’s Hospital and Harvard T.H. Chan School of Public Health, and those of participating registries as required. We would like to thank the participants and staff of the NHS and NHS2 for their valuable contributions as well as the following state cancer registries for their help: AL, AZ, AR, CA, CO, CT, DE, FL, GA, ID, IL, IN, IA, KY, LA, ME, MD, MA, MI, NE, NH, NJ, NY, NC, ND, OH, OK, OR, PA, RI, SC, TN, TX, VA, WA, WY. The authors assume full responsibility for analyses and interpretation of these data.

The **OFBCR** thanks Teresa Selander, Nayana Weerasooriya and Steve Gallinger

**PBCS** thanks Louise Brinton, Mark Sherman, Neonila Szeszenia-Dabrowska, Beata Peplonska, Witold Zatonski, Pei Chao, Michael Stagner.

**SASBAC** thank the Swedish Medical Research Counsel.

We thank the **SIBS** and **EPIC** teams.

**UKBGS** thanks Breast Cancer Now and the Institute of Cancer Research for support and funding of the Generations Study, and the study participants, study staff, and the doctors, nurses and other health care providers and health information sources who have contributed to the study. We acknowledge NHS funding to the Royal Marsden/ICR NIHR Biomedical Research Centre.

**The Genotype-Tissue Expression (GTEx) Project** was supported by the Common Fund of the Office of the Director of the National Institutes of Health (commonfund.nih.gov/GTEx). Additional funds were provided by the NCI, NHGRI, NHLBI, NIDA, NIMH, and NINDS. Donors were enrolled at Biospecimen Source Sites funded by NCI\Leidos Biomedical Research, Inc. subcontracts to the National Disease Research Interchange (10XS170), Roswell Park Cancer Institute (10XS171), and Science Care, Inc. (X10S172). The Laboratory, Data Analysis, and Coordinating Center (LDACC) was funded through a contract (HHSN268201000029C) to the The Broad Institute, Inc. Biorepository operations were funded through a Leidos Biomedical Research, Inc. subcontract to Van Andel Research Institute (10ST1035). Additional data repository and project management were provided by Leidos Biomedical Research, Inc. (HHSN261200800001E). The Brain Bank was supported supplements to University of Miami grant DA006227. Statistical Methods development grants were made to the University of Geneva (MH090941 & MH101814), the University of Chicago (MH090951, MH090937, MH101825, & MH101820), the University of North Carolina - Chapel Hill (MH090936), North Carolina State University (MH101819), Harvard University (MH090948), Stanford University (MH101782), Washington University (MH101810), and to the University of Pennsylvania (MH101822). The datasets used for the analyses described in this manuscript were obtained from dbGaP at http://www.ncbi.nlm.nih.gov/gap through dbGaP accession number phs000424.v8.p2

**SUPLLEMENTARY FIGURES**

**Supplementary Figure 1.** Distribution of age (left, N = 17,162) and BMI (right, N = 17,162) at mammogram collection (top panel), and square-root transformed mammographic density measures (DA (left), NDA (middle) and PMD (right), bottom panel). Only individuals with genotype and phenotype data collected were used to generate the distribution of DA, NDA, and PMD, which had a sample size of 13,805, 13,915, and 17,126, respectively.


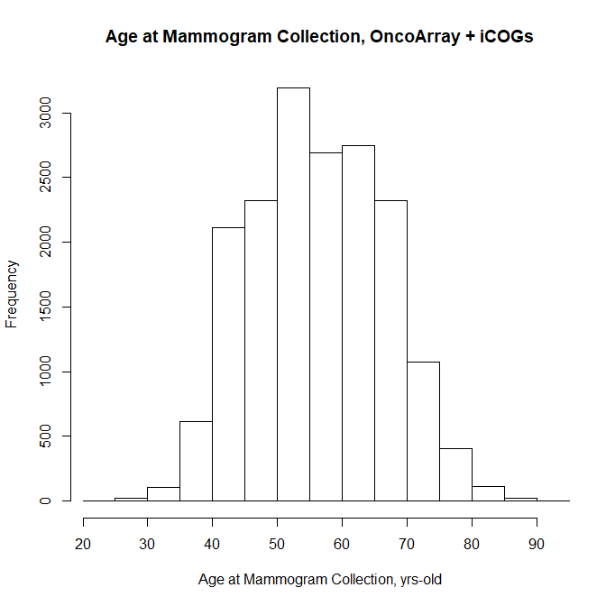

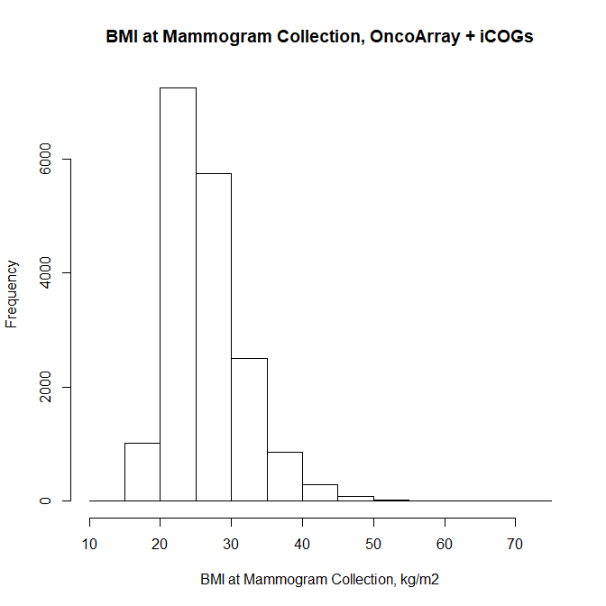


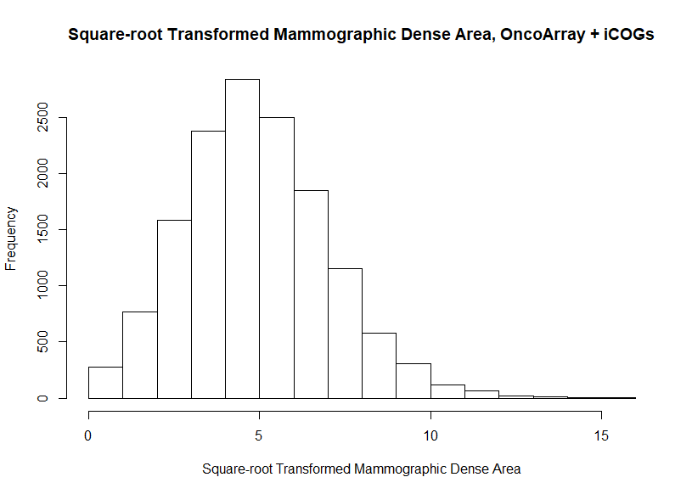

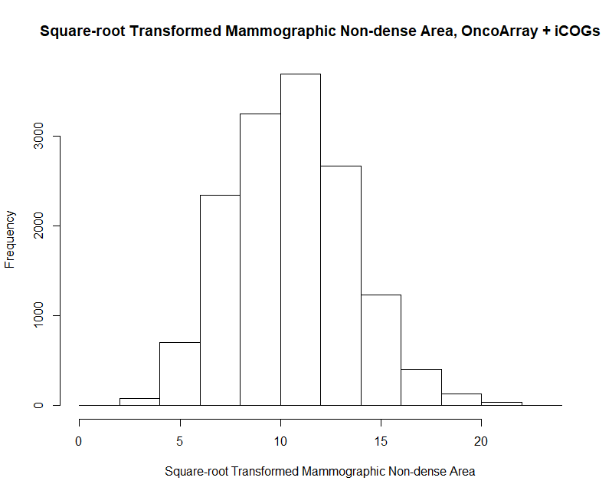

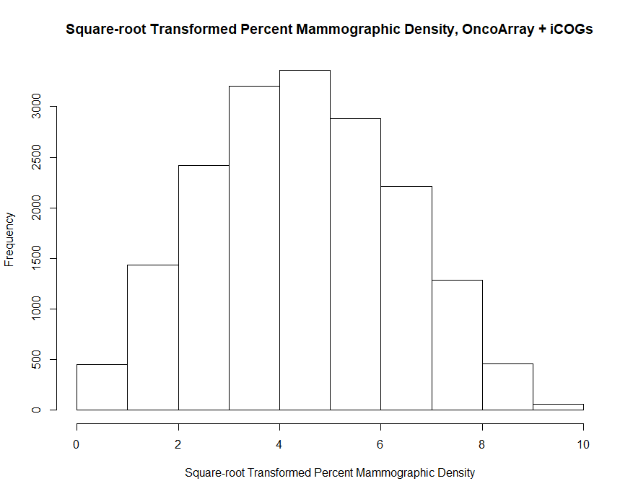


**Supplementary Figure 2.** Quantile-Quantile plots of the GWAS meta-analysis results for mammographic dense area, DA; non-dense area, NDA; and percent mammographic density, PMD. The genomic inflation factors (λ_GC_) were estimated as 1.11, 1.11 and 1.13 for the meta-analysis results of DA, NDA and PMD, respectively.

**
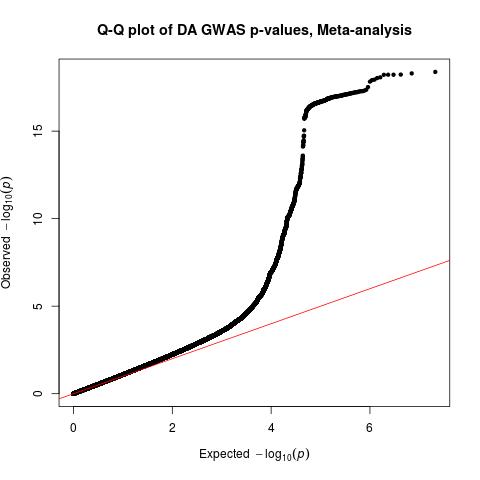

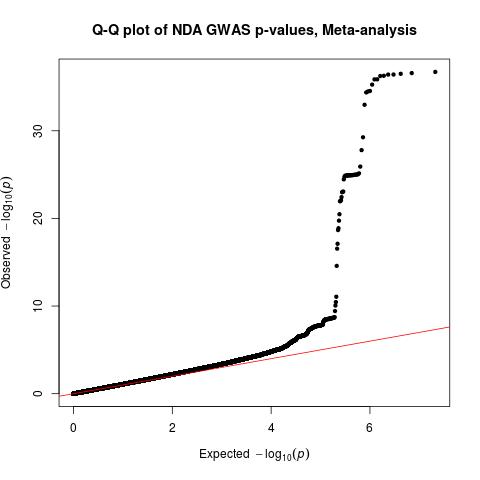

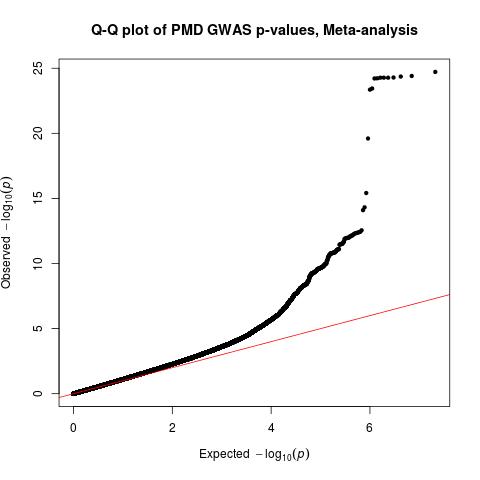
**

**Supplementary Figure 3.** Regional association plots for 18 genome-wide significant loci of DA. The European ancestry population (EUR) in the 1000 Genomes project was used as the reference panel for linkage disequilibrium (LD) estimates (shown as r^2^). Color of the dots indicated the extent of LD. Recombination rates were estimated using EUR samples in 1000 Genomes. Physical positions were based Genomic build hg19.


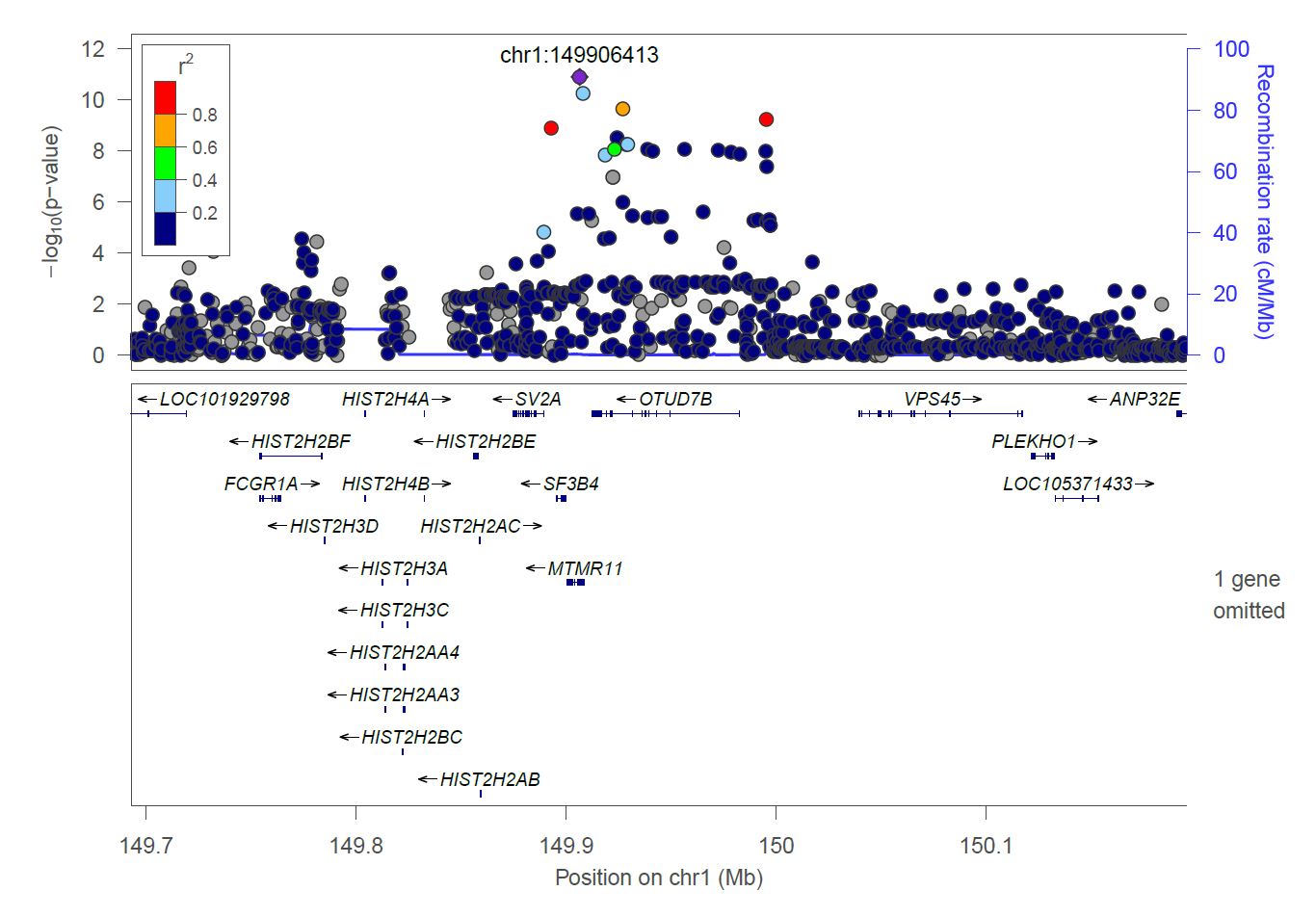


**1q21.2**

**
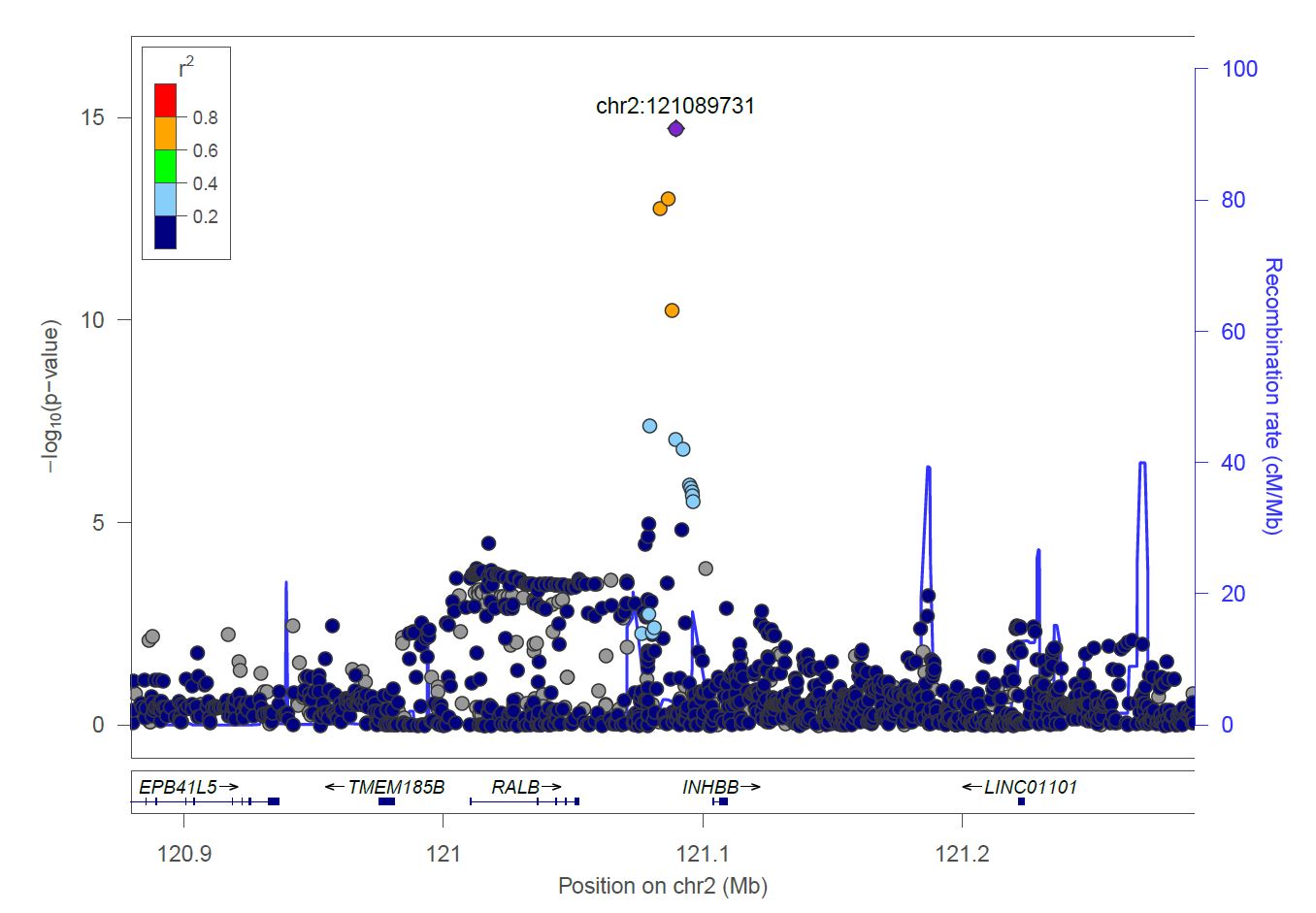
2q14.2**

**
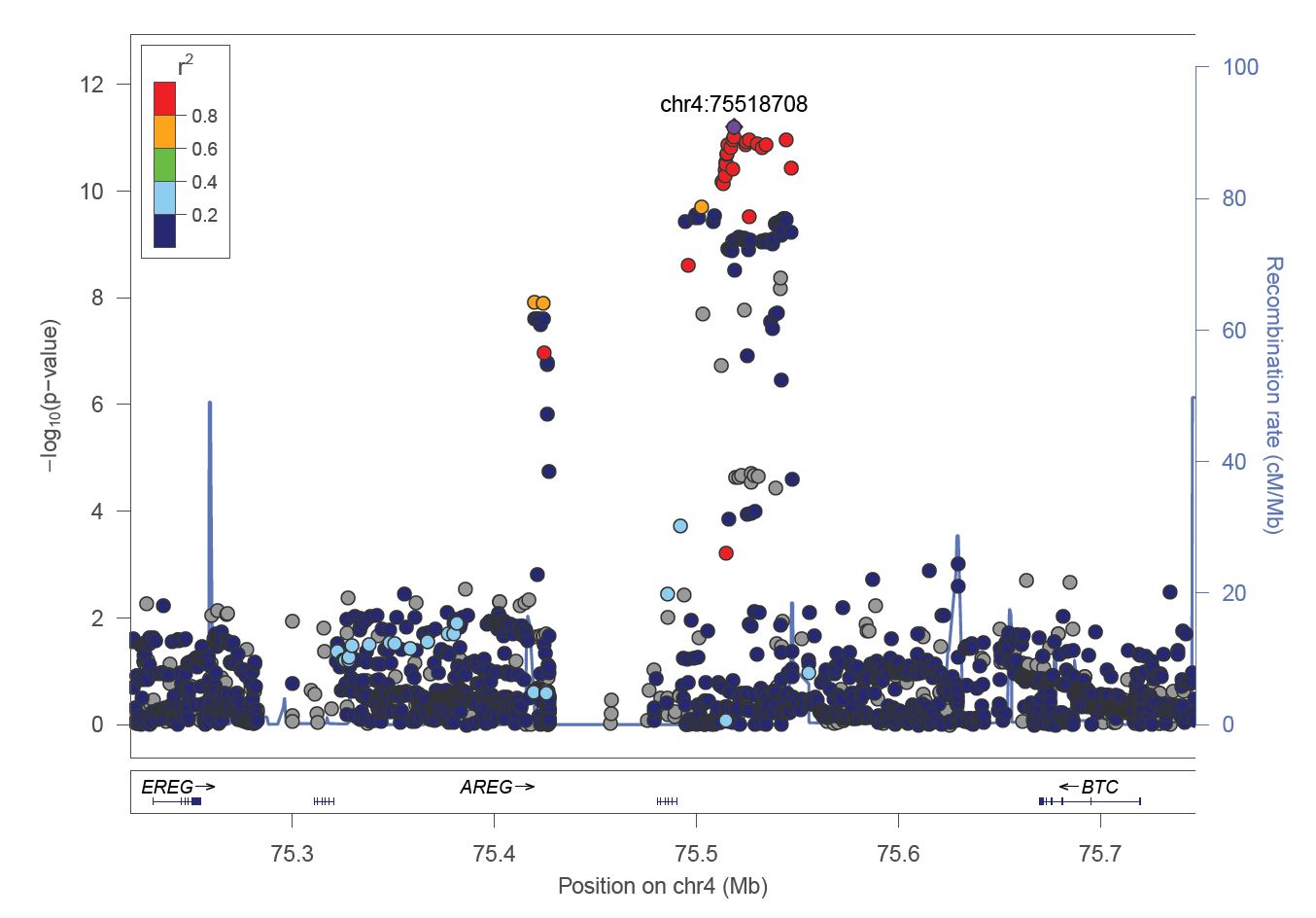
**

**4q13.3**

**
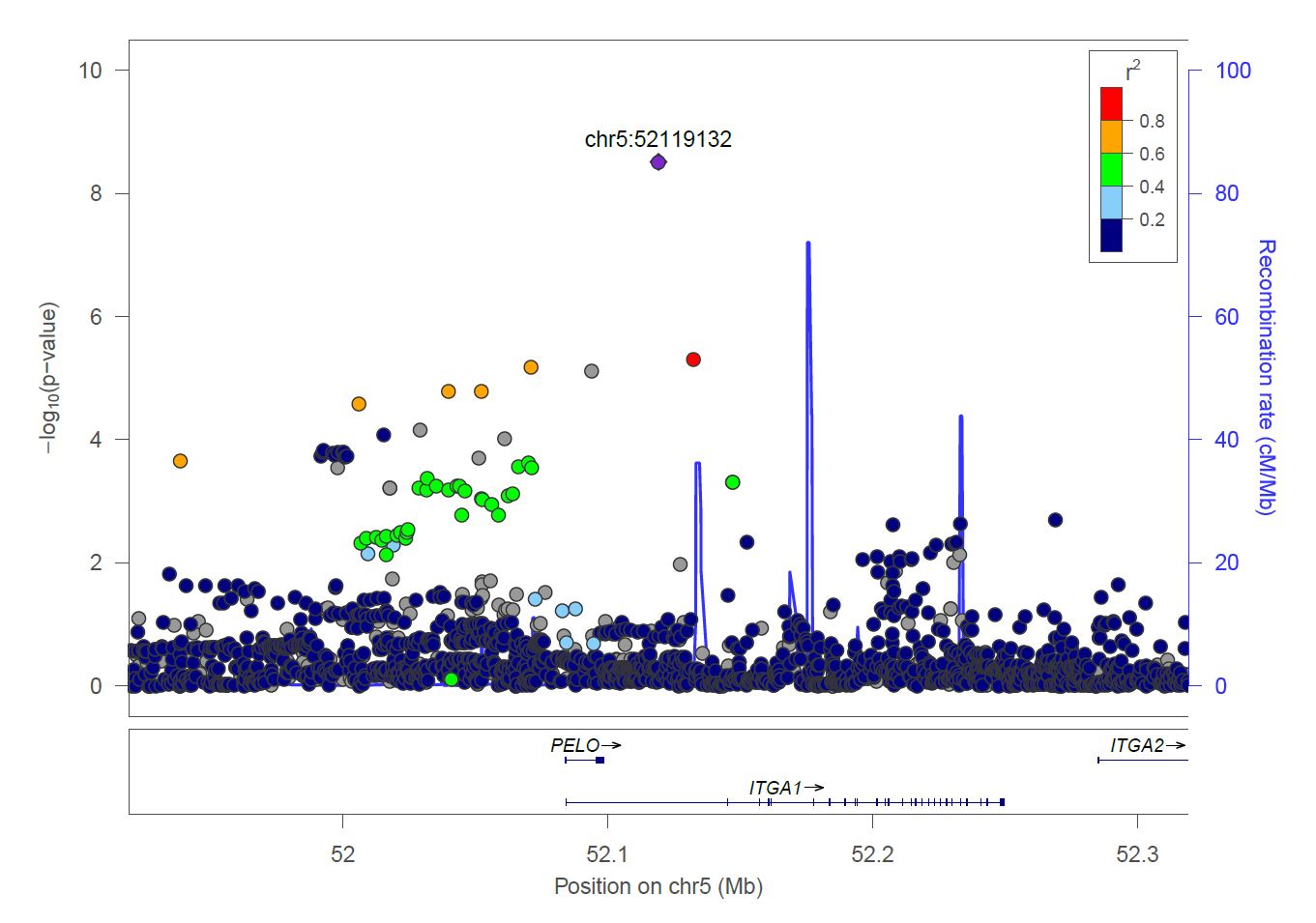
**

**5q11.2**

**
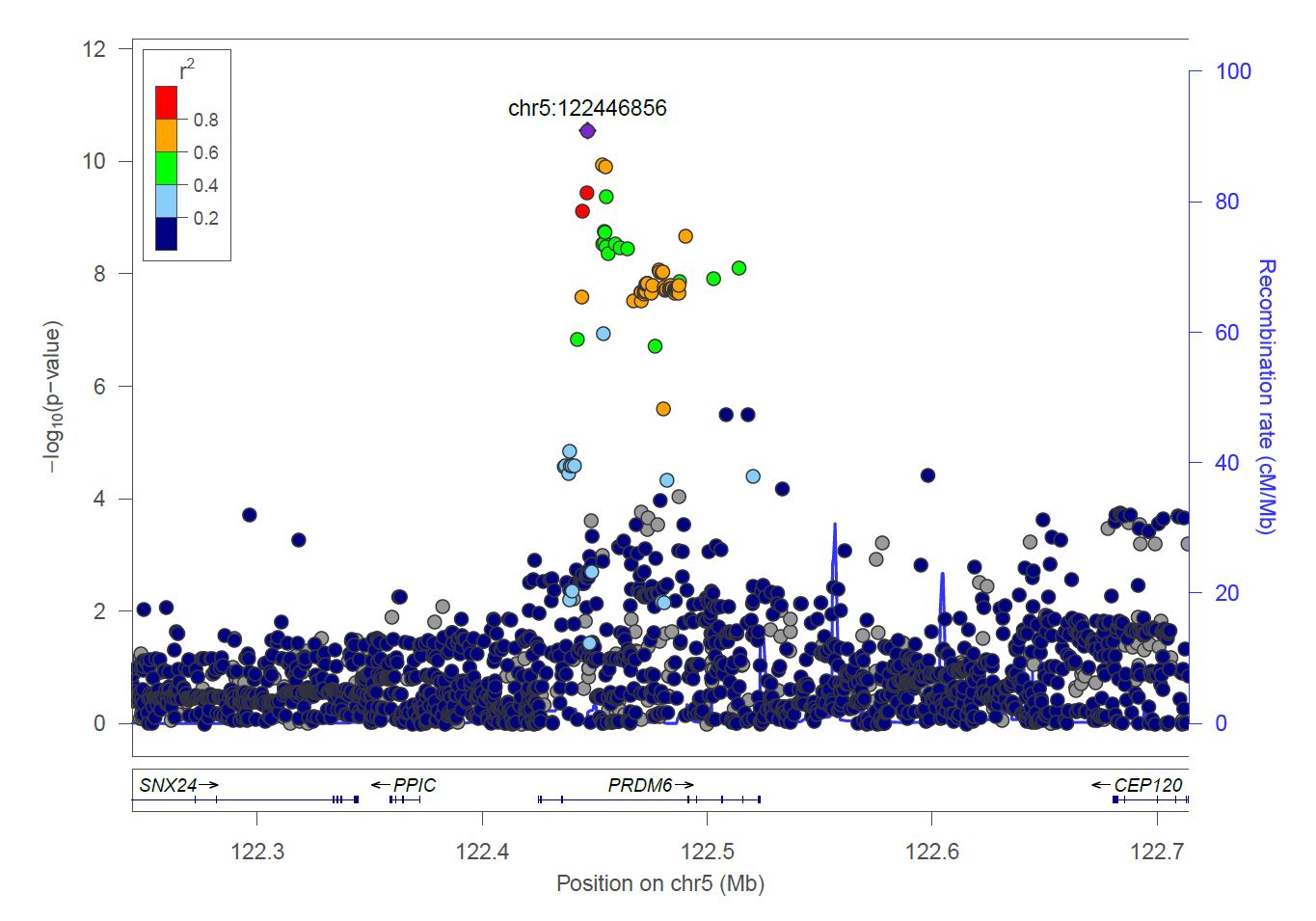
**

**5q23.2**

**
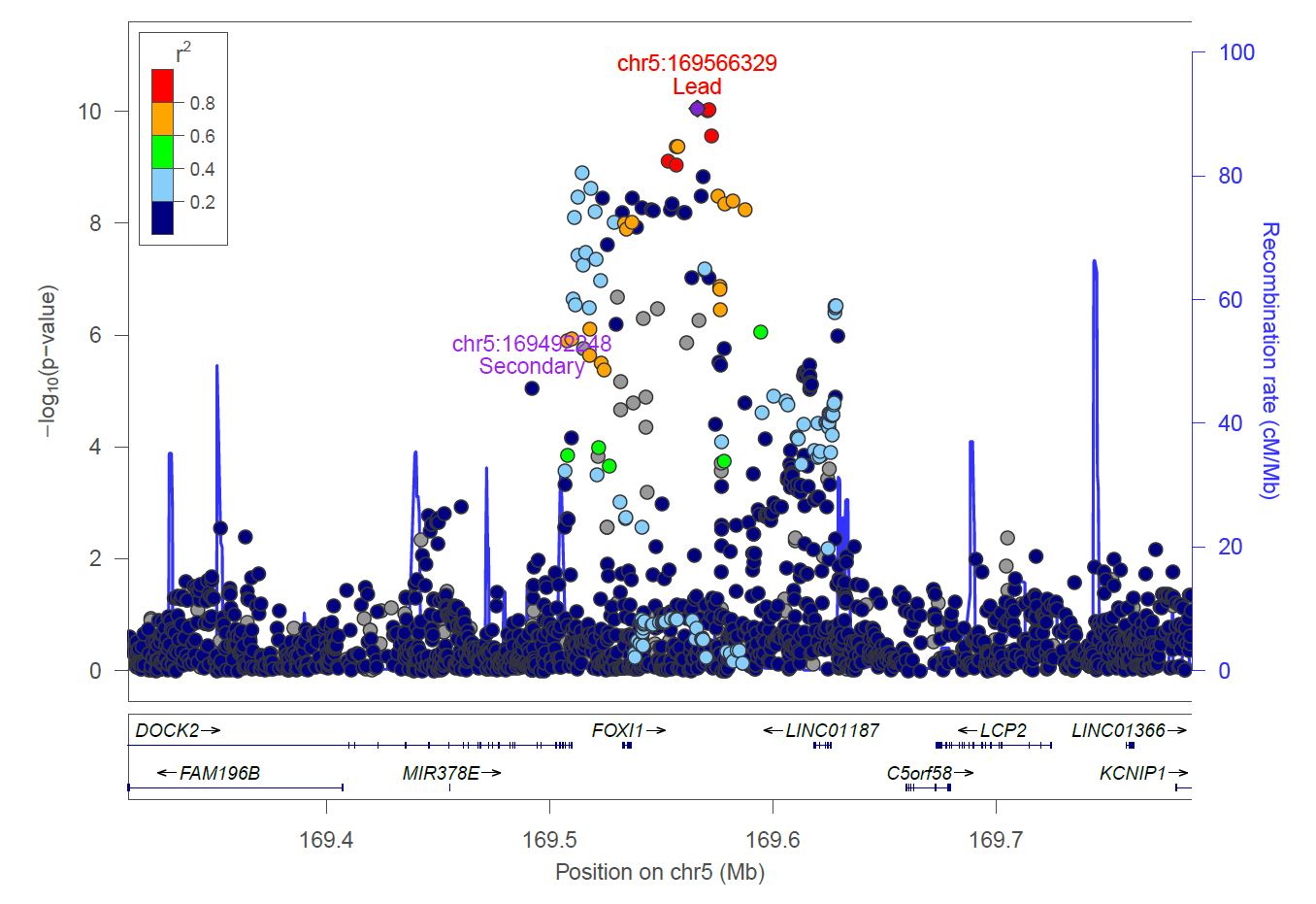
**

**5q35.1**

**
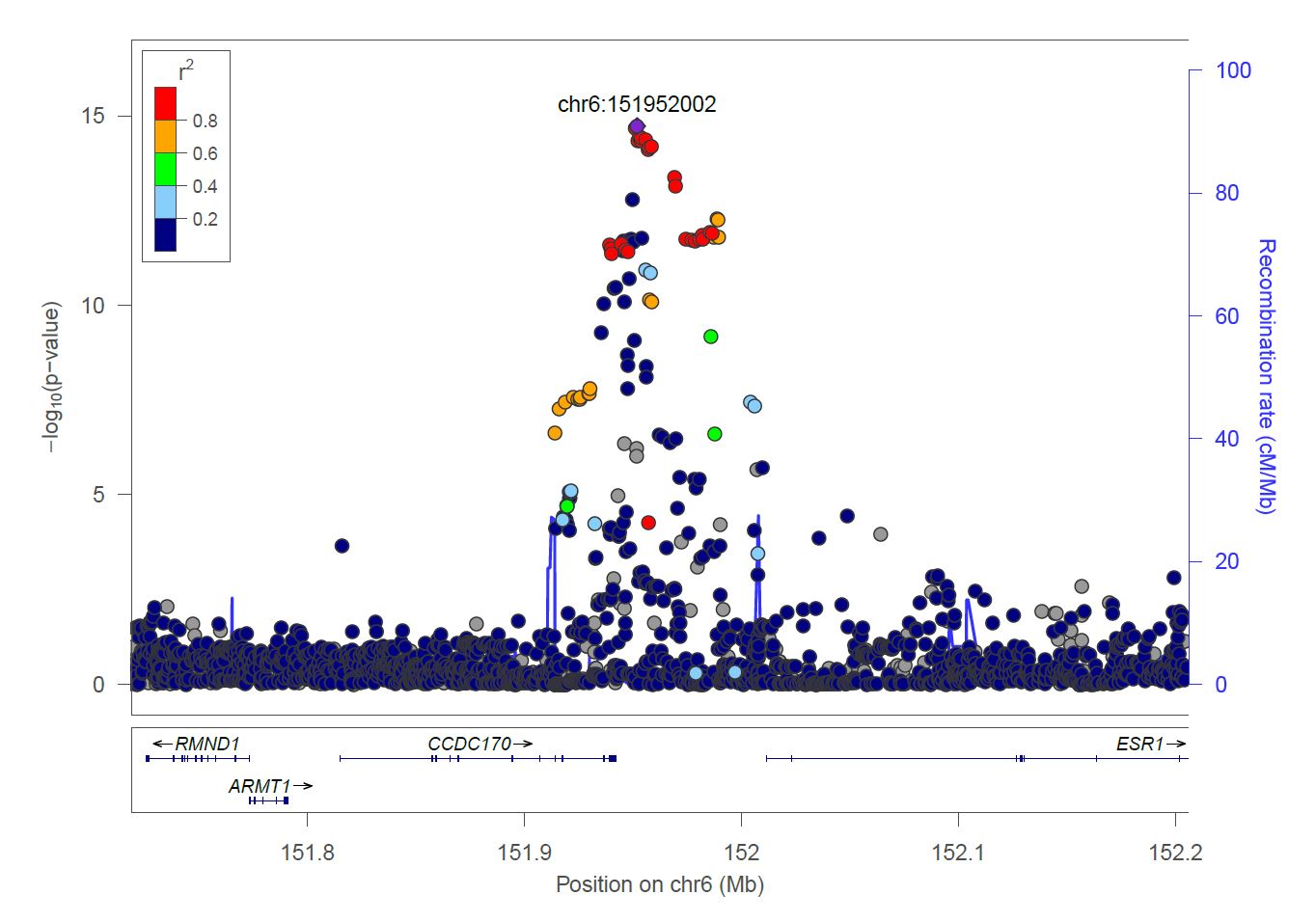
**

**6q25.1**

**
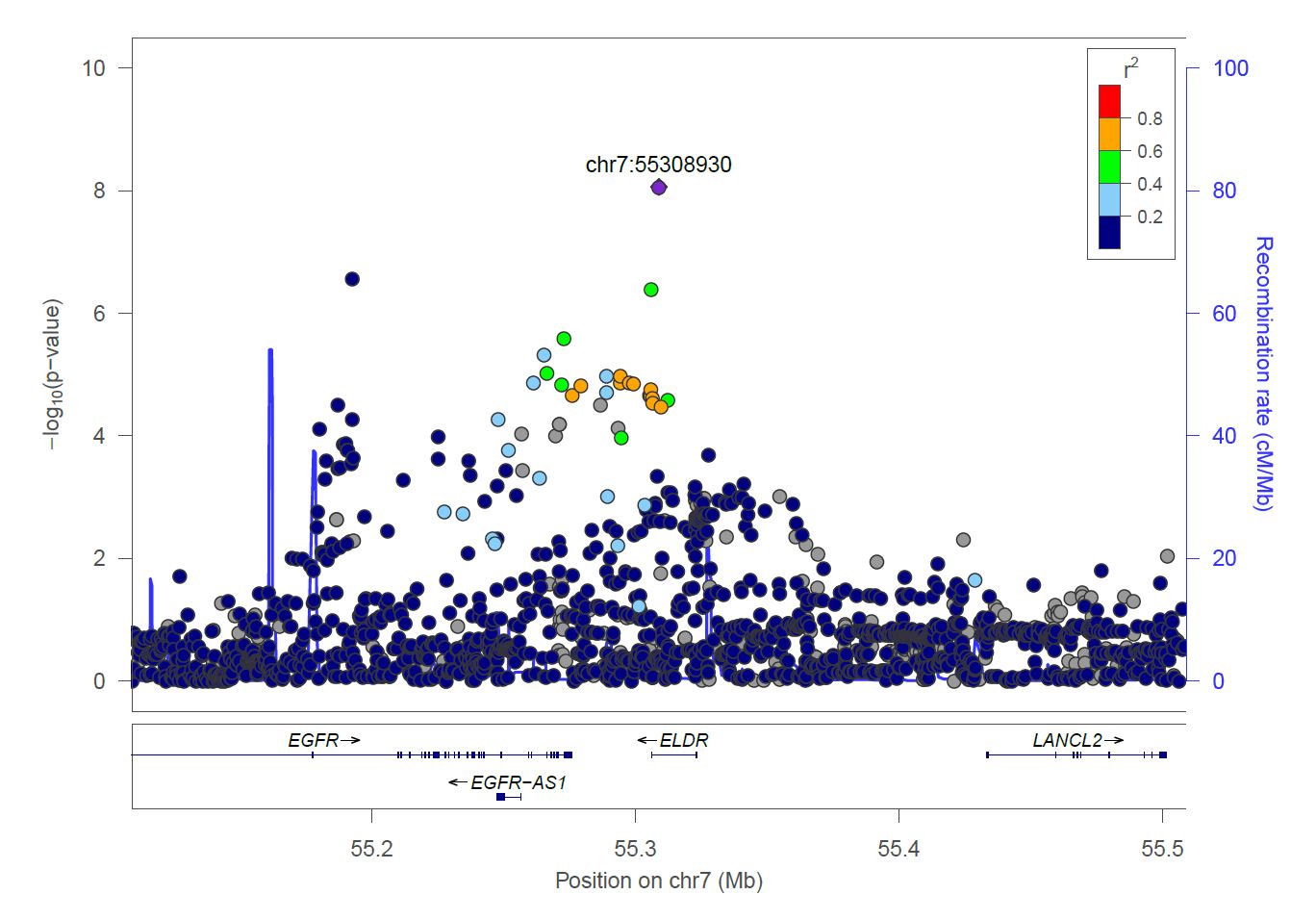
**

**7p11.2**

**
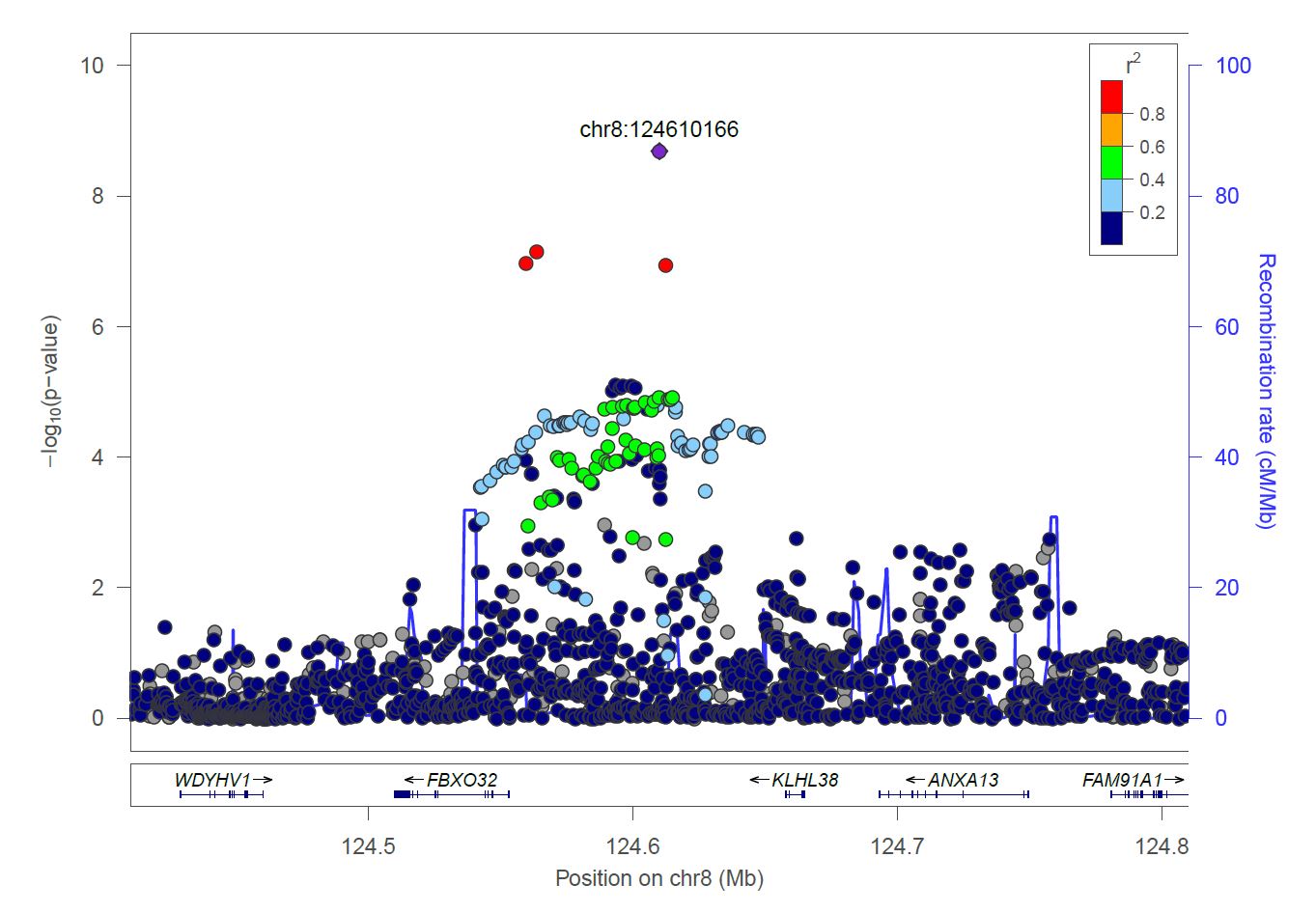
**

**8q24.13**

**
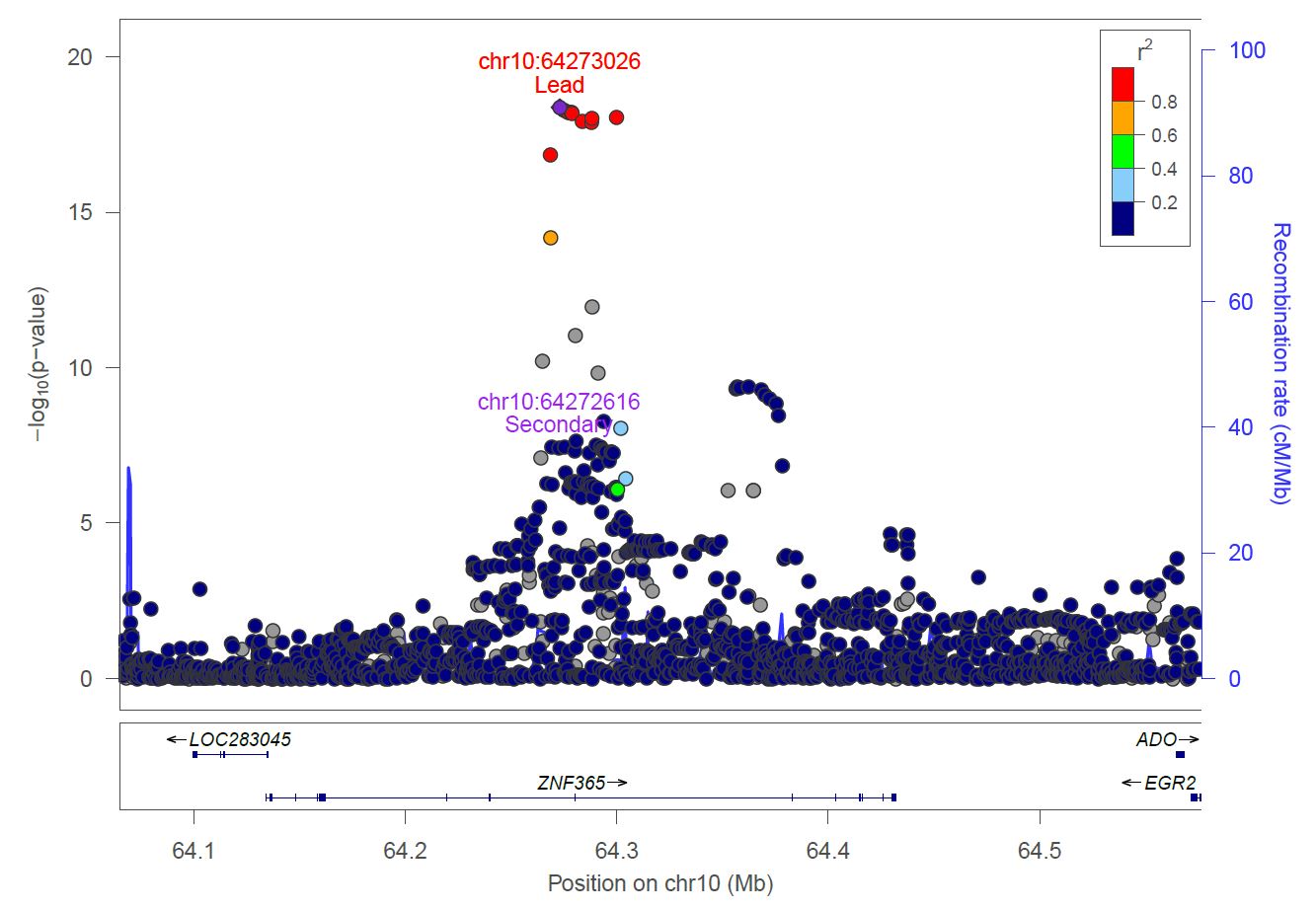
**

**10q21.2**

**
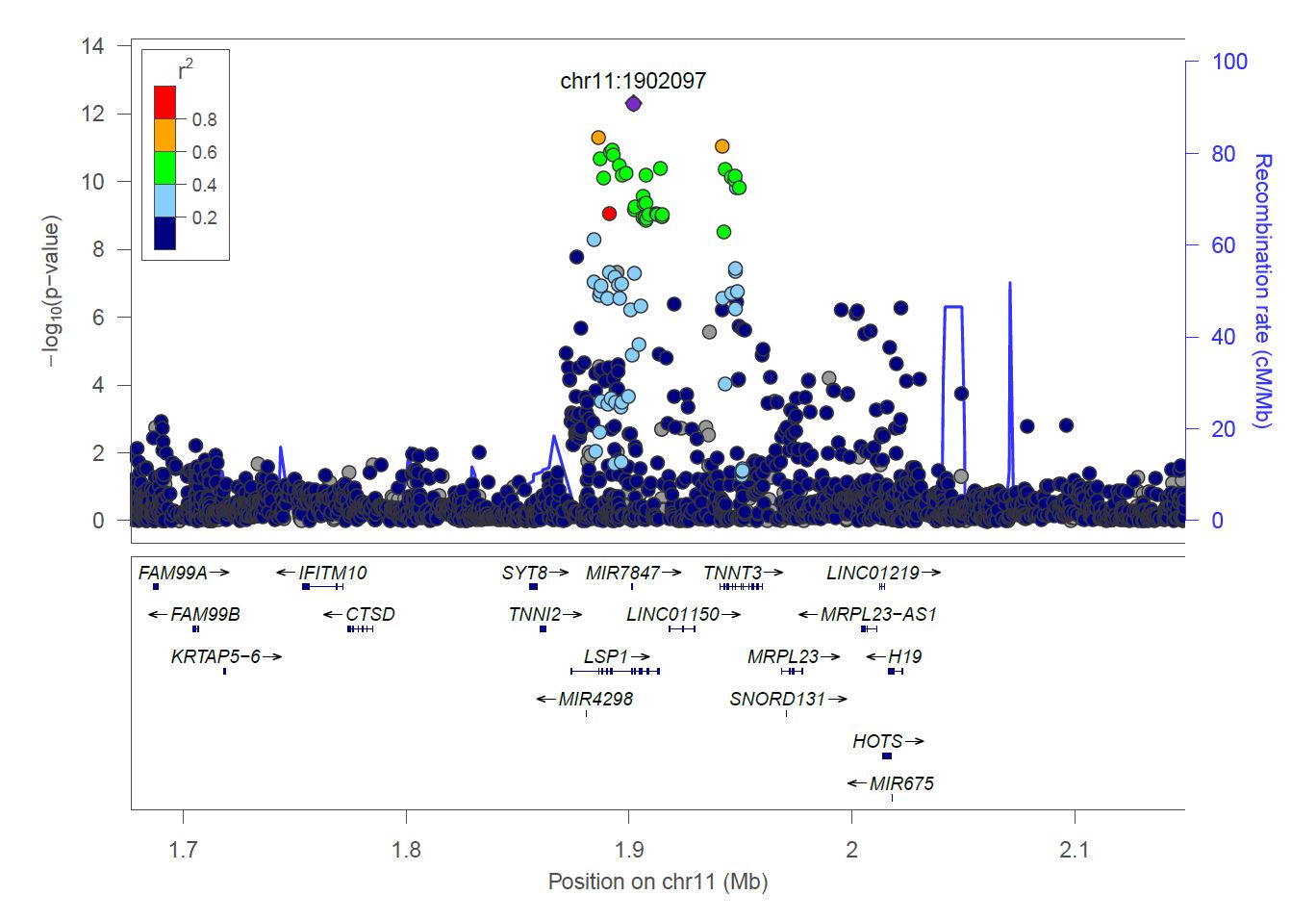
**

**11p15.5**

**
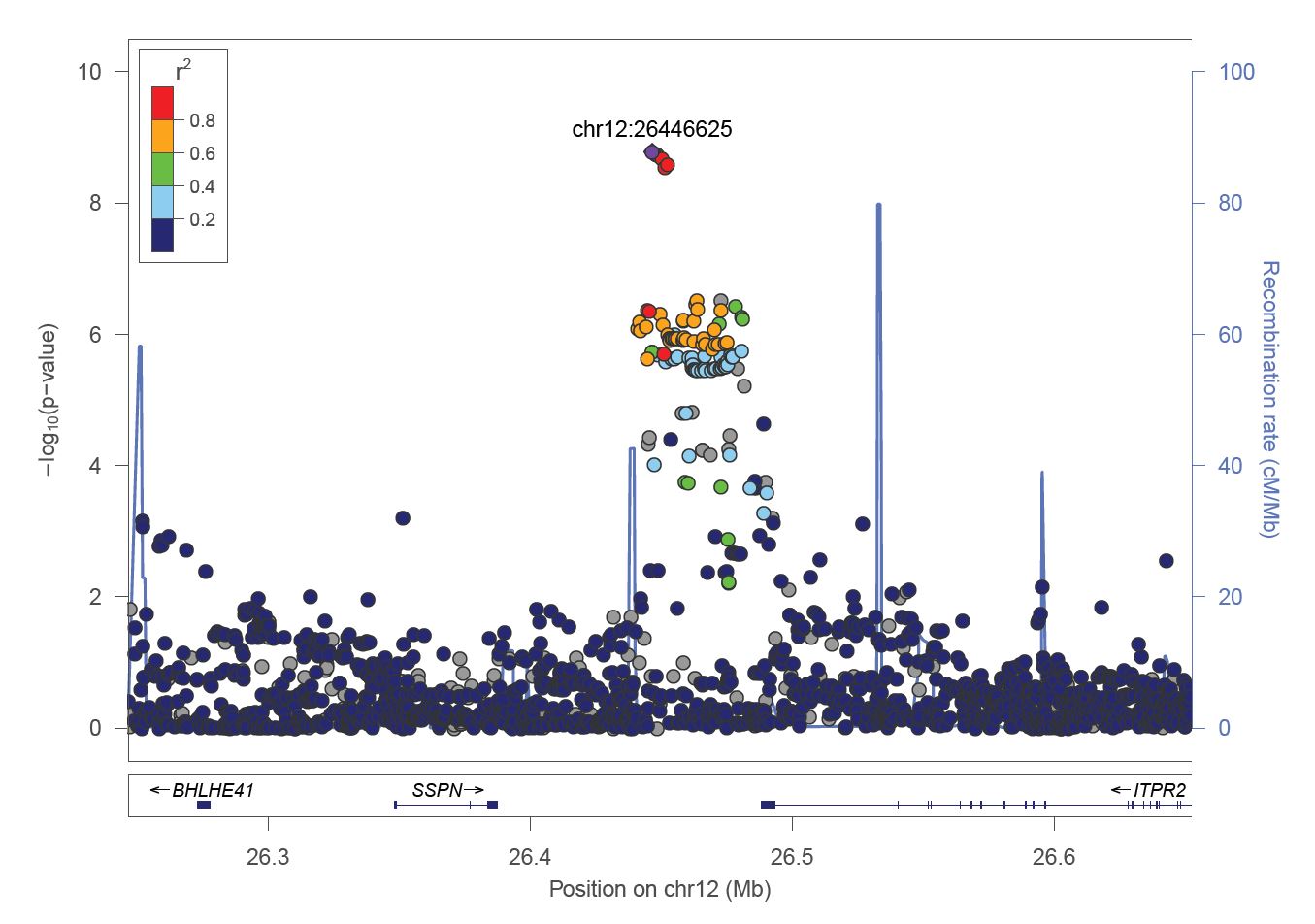
**

**12p12.1**

**
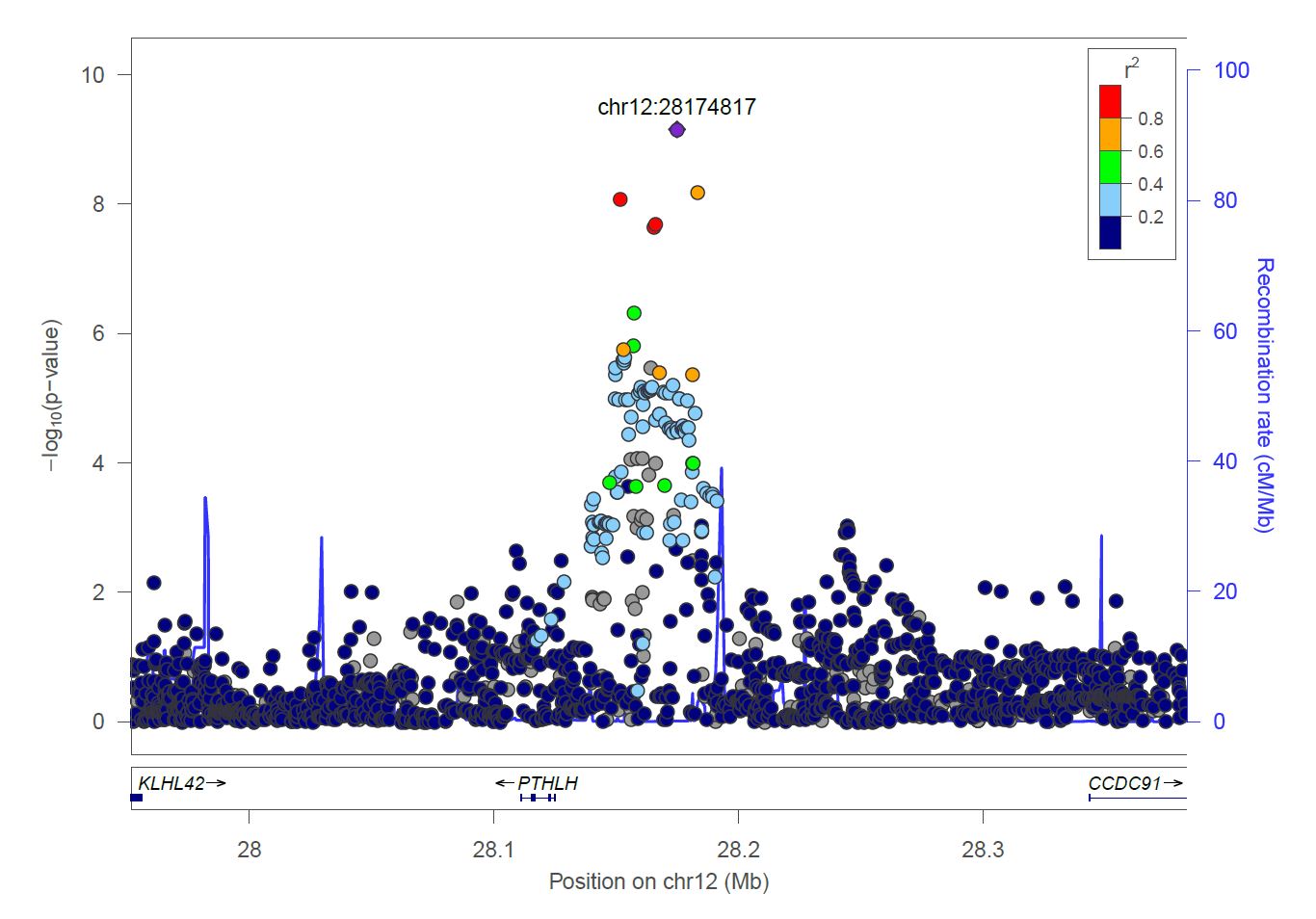
**

**12p11.2**

**
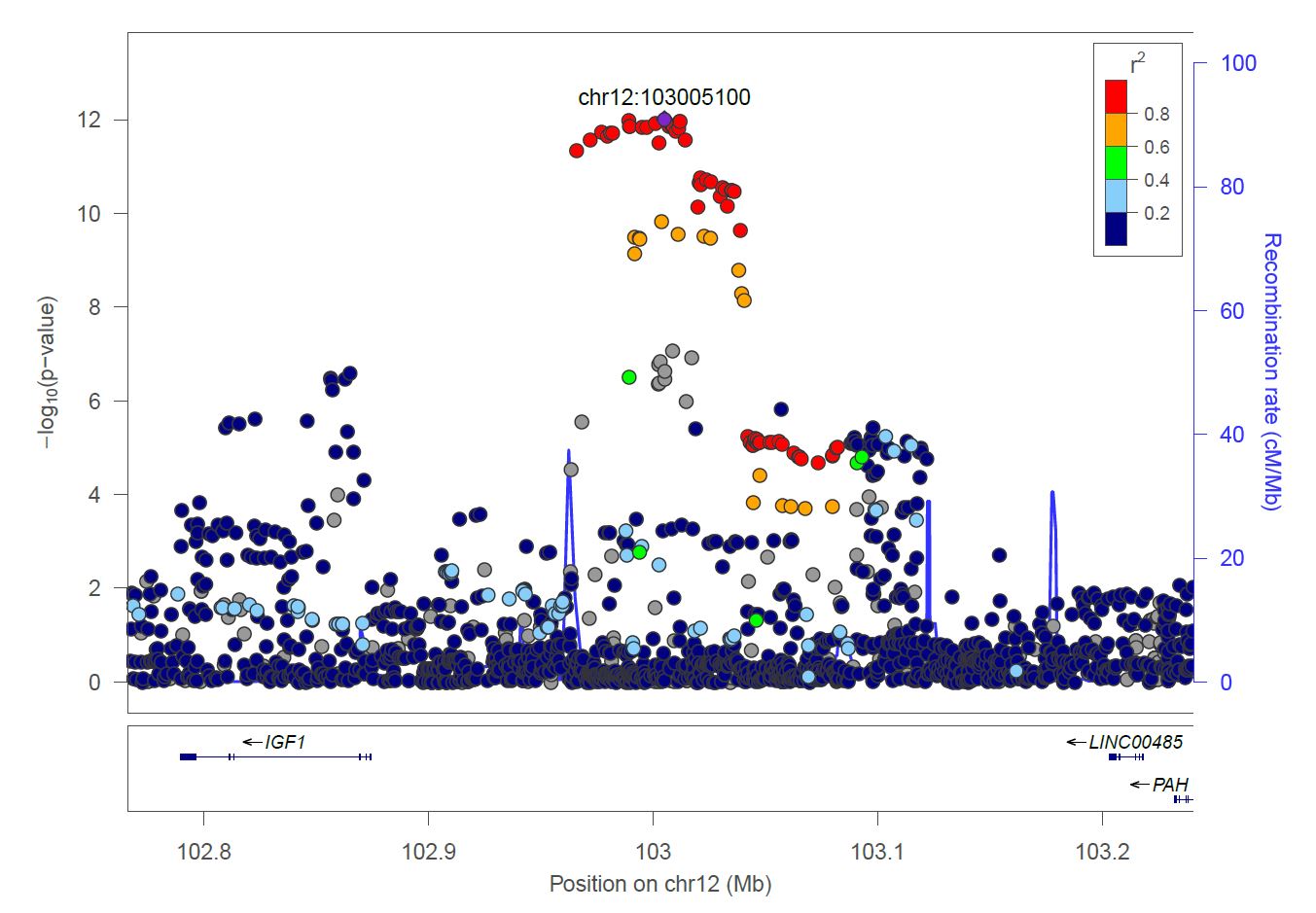
**

**12q23.2**

**
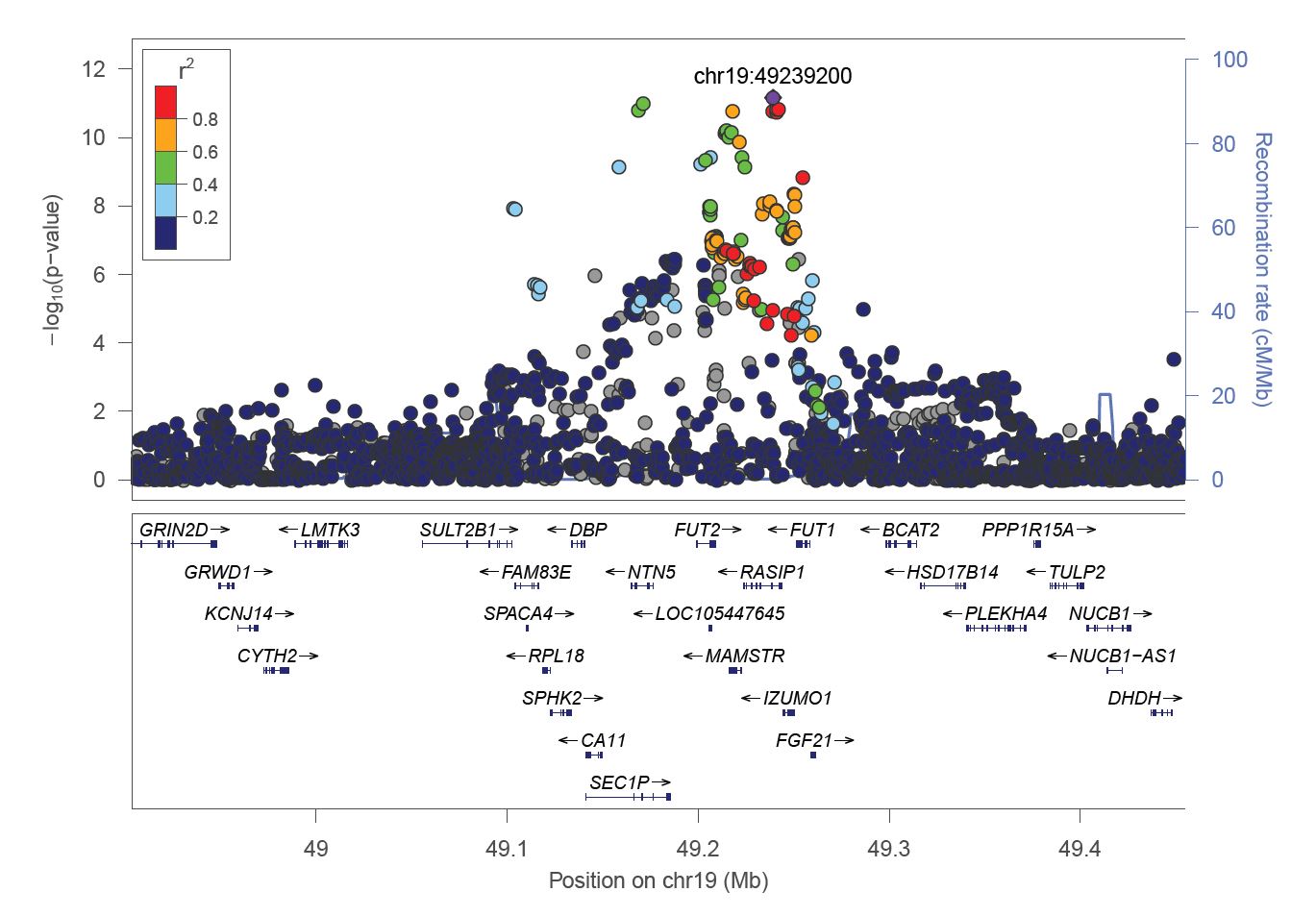
**

**19q13.33**

**
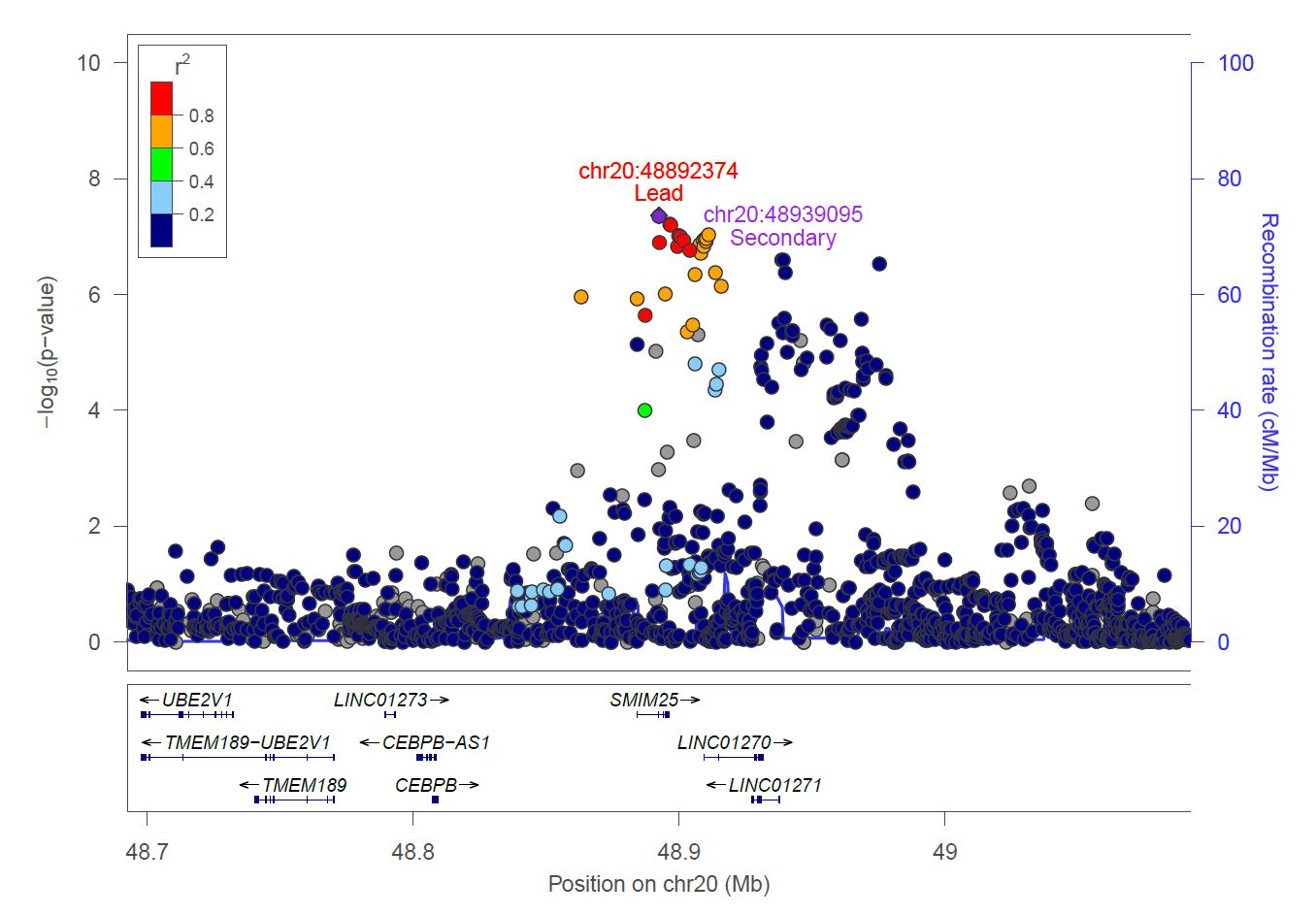
**

**20q13.13**

**
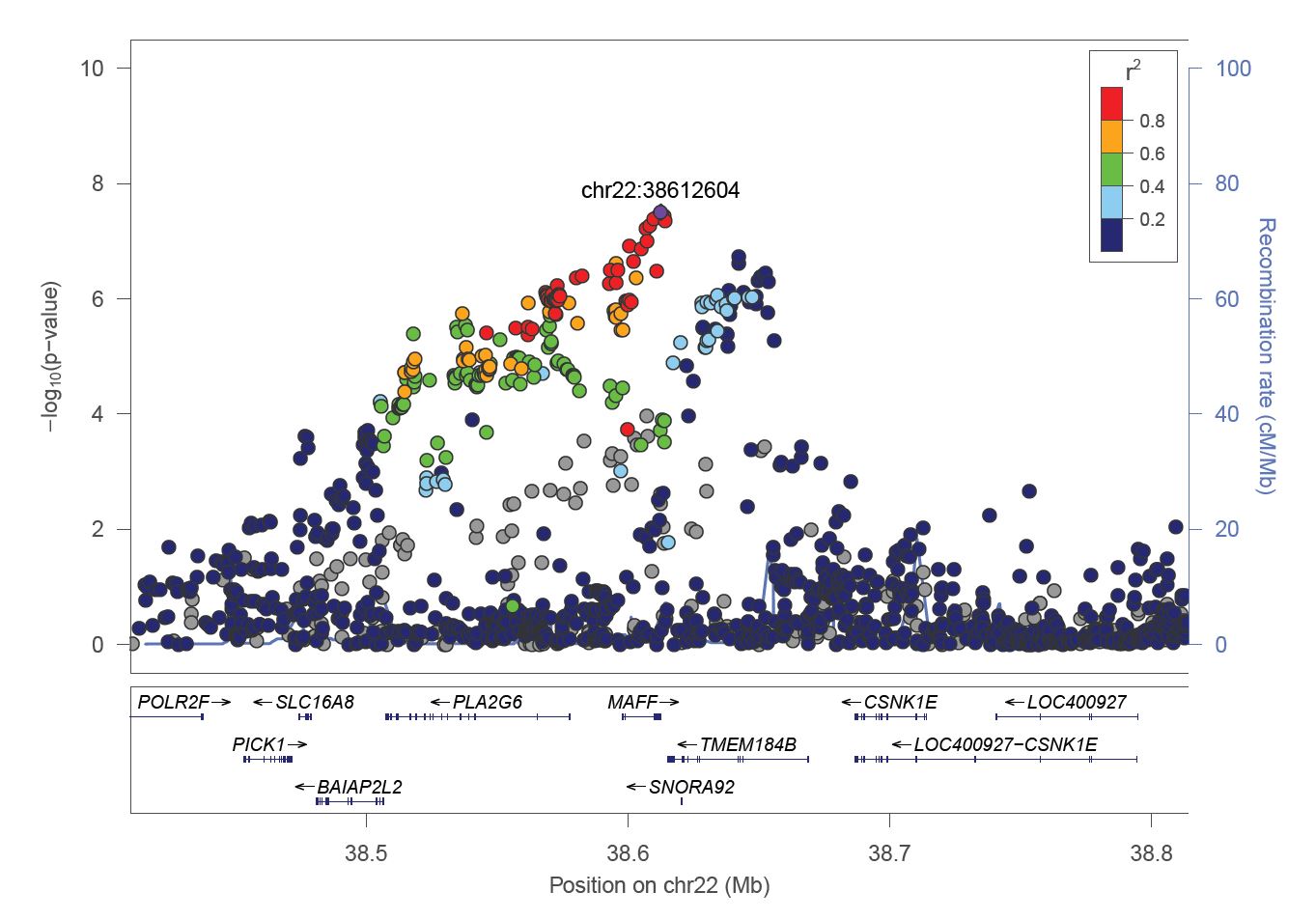
**

**22q13.1**

**
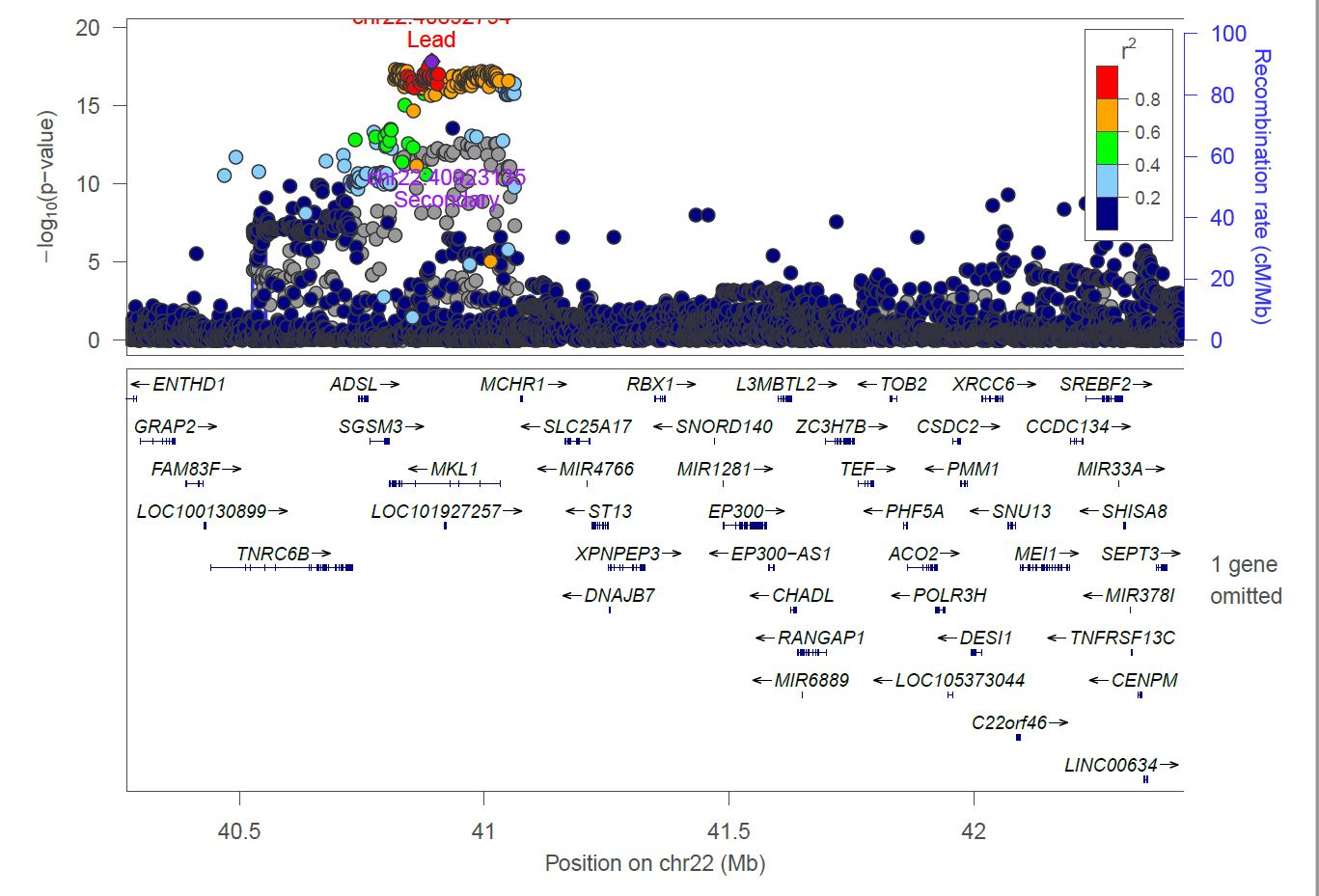
**

**22q13.2**

**Supplementary Figure 4.** Regional association plots for six genome-wide significant loci of NDA. The European ancestry population (EUR) in the 1000 Genomes project was used as the reference panel for linkage disequilibrium (LD) estimates (shown as r^2^). Color of the dots indicated the extent of LD. Recombination rates were estimated using EUR samples in 1000 Genomes. Physical positions were based Genomic build hg19.

**
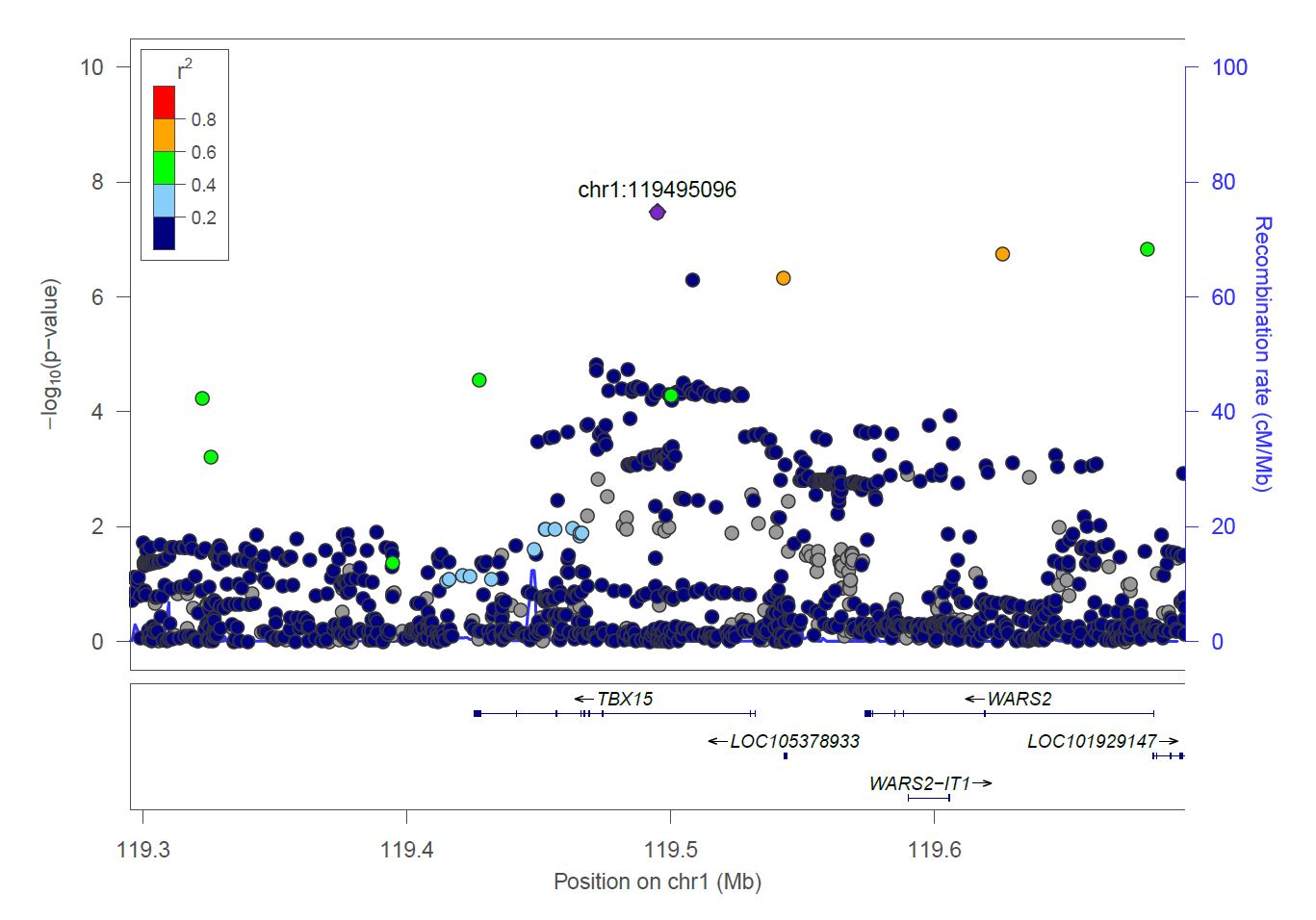
**

**1p12**

**
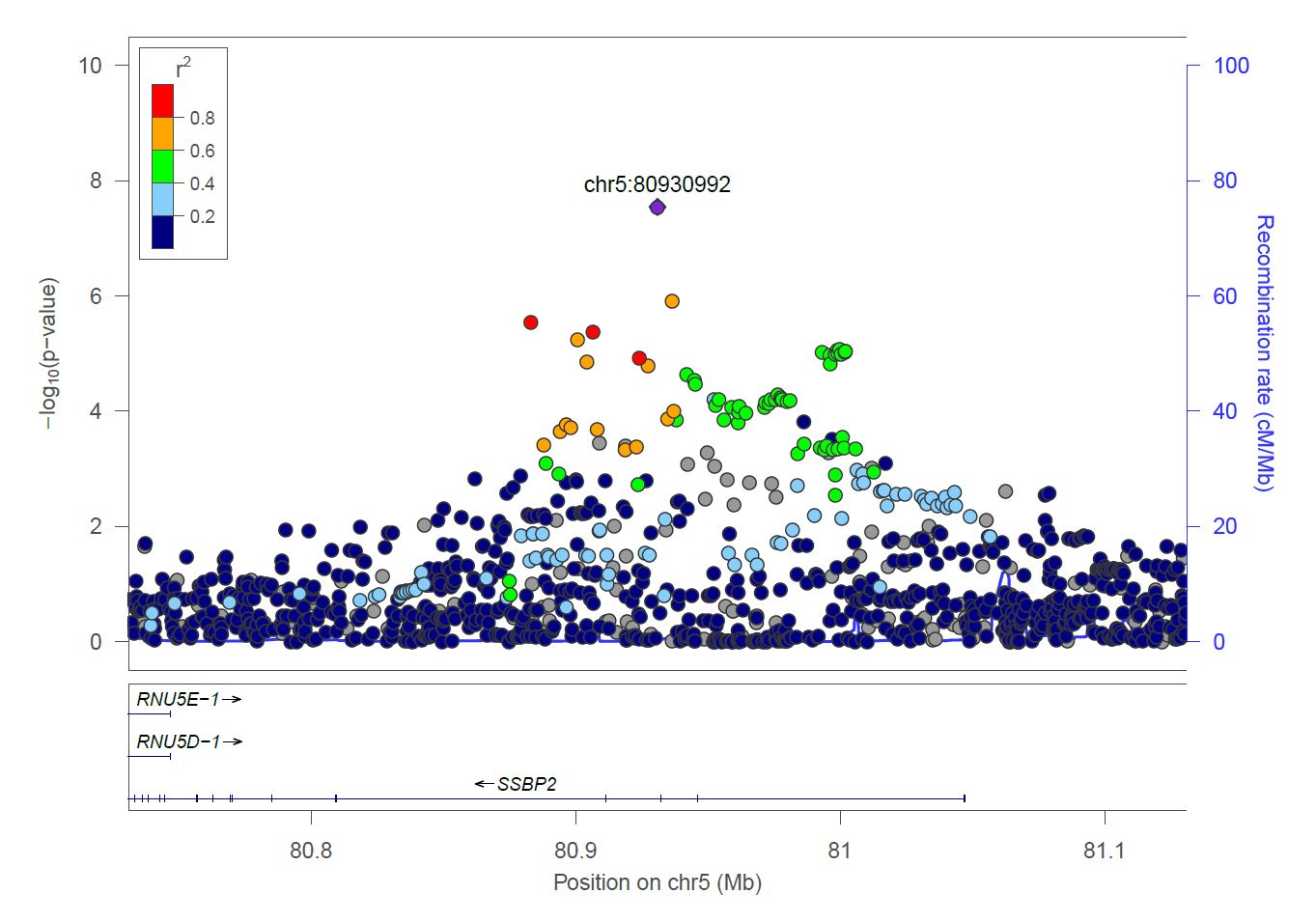
**

**5q14.1**

**
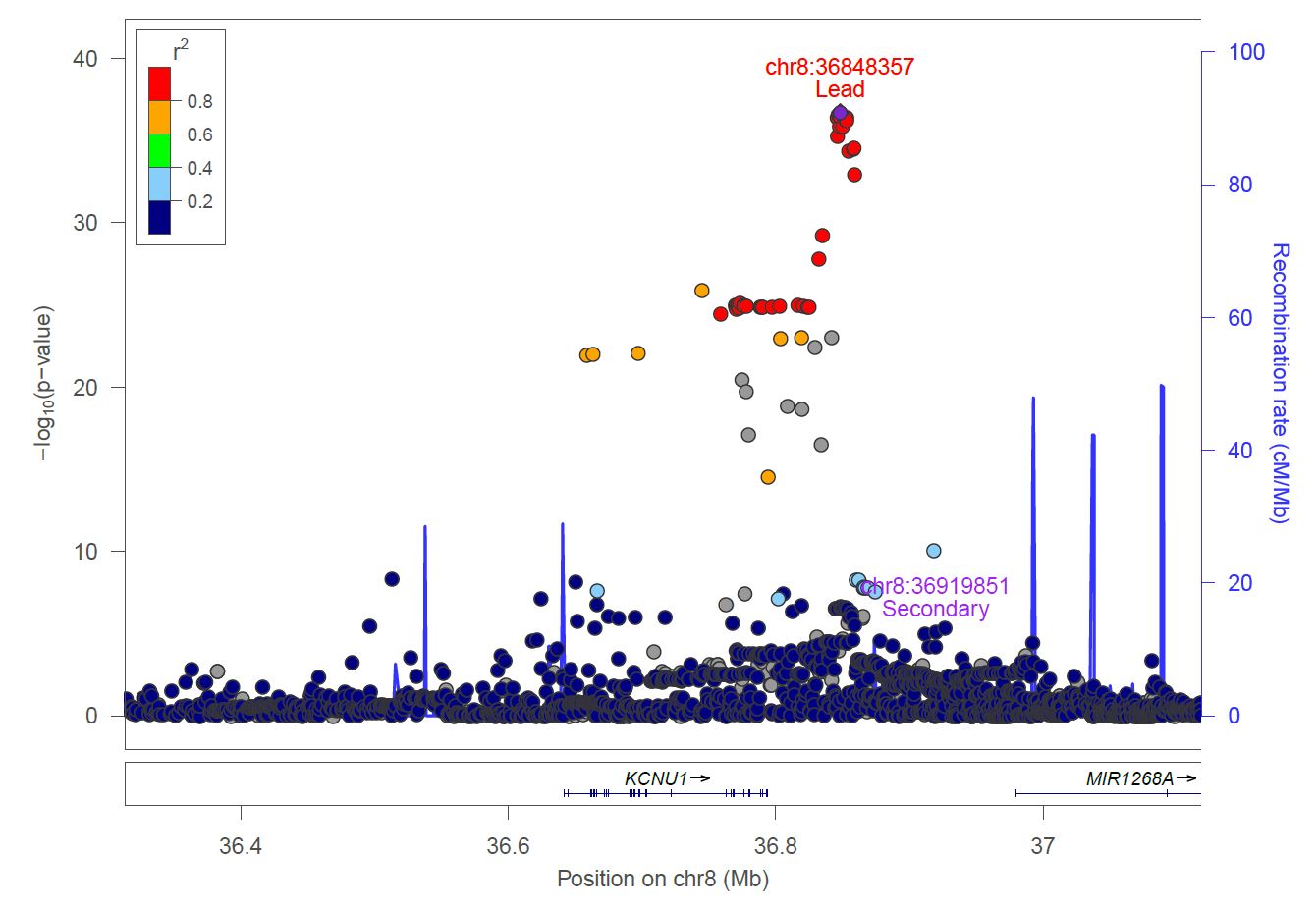
**

**8p11.23**

**
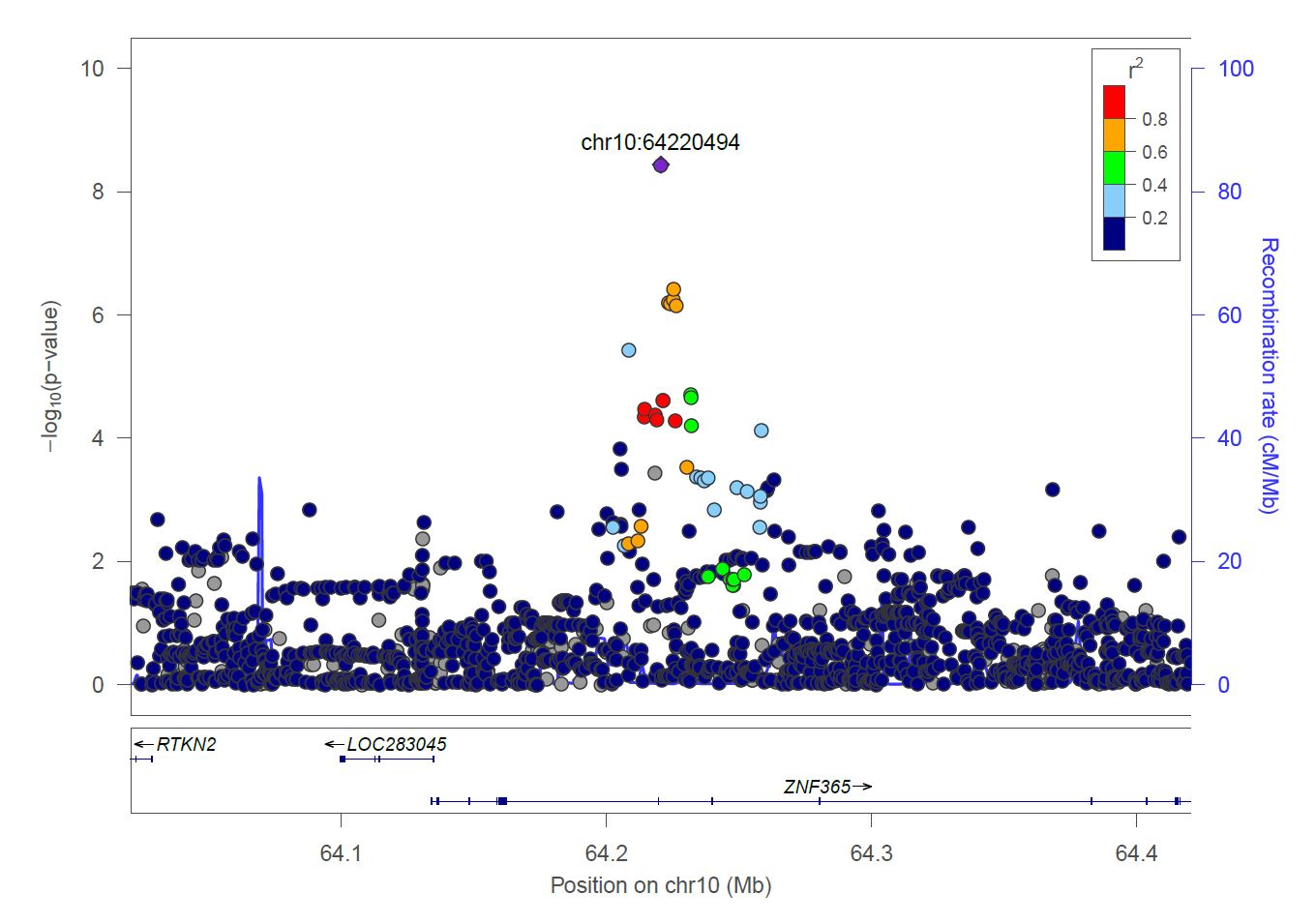
**

**10q21.2**

**
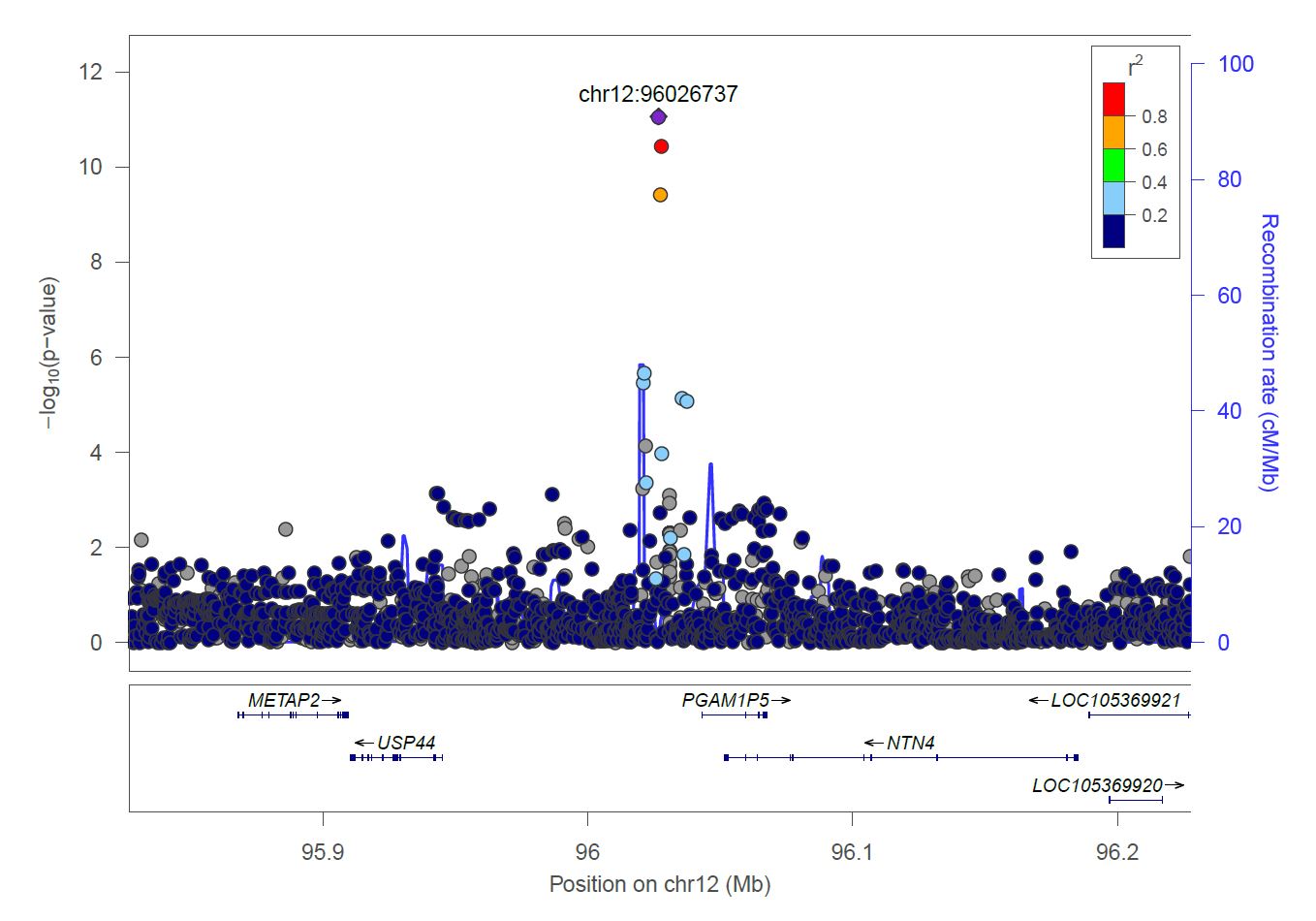
**

**12q22**

**
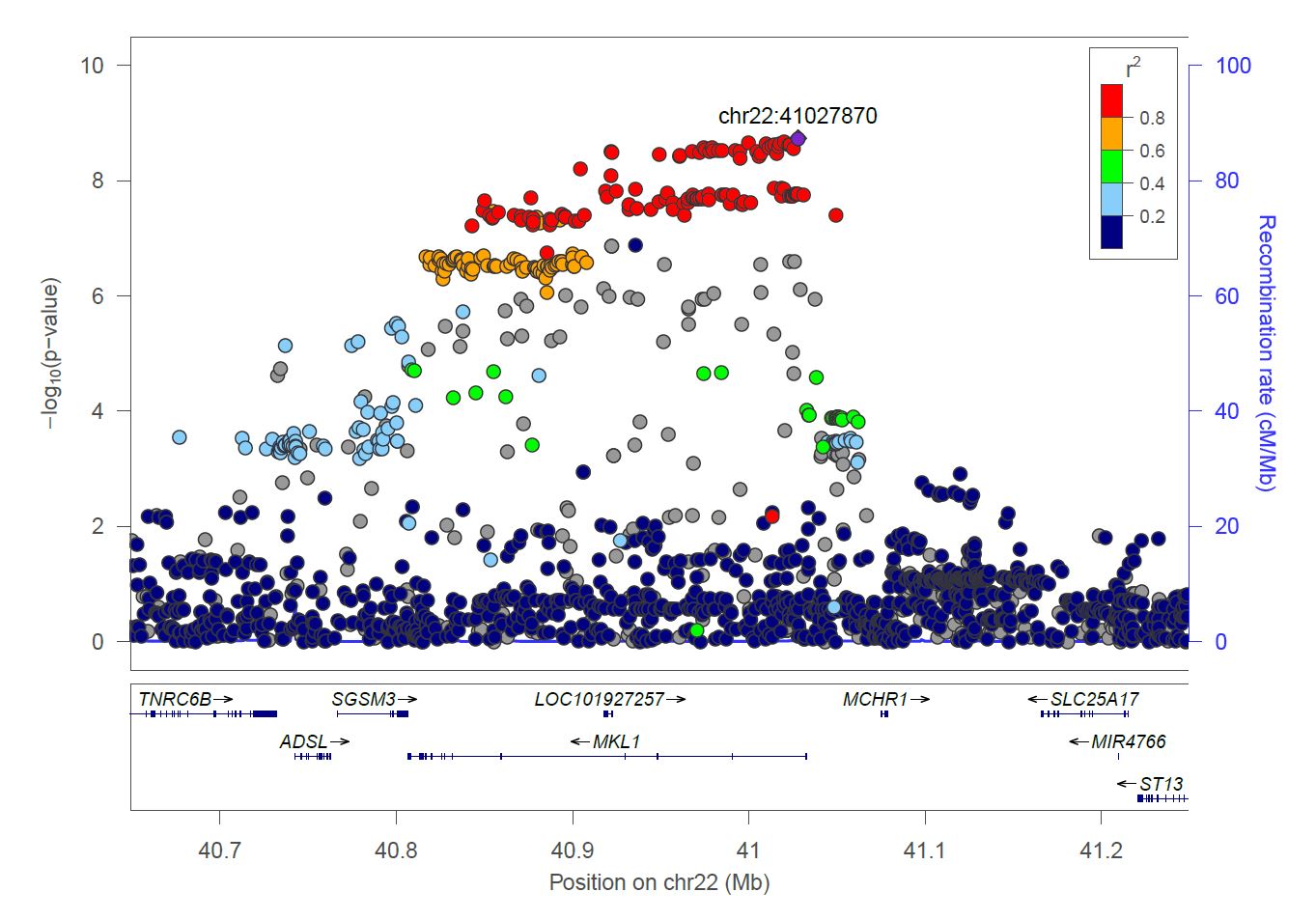
**

**22q13.2**

**Supplementary Figure 5.** Regional association plots for 15 genome-wide significant loci of PMD. The European ancestry population (EUR) in the 1000 Genomes project was used as the reference panel for linkage disequilibrium (LD) estimates (shown as r^2^). Color of the dots indicated the extent of LD. Recombination rates were estimated using EUR samples in 1000 Genomes. Physical positions were based Genomic build hg19.

**
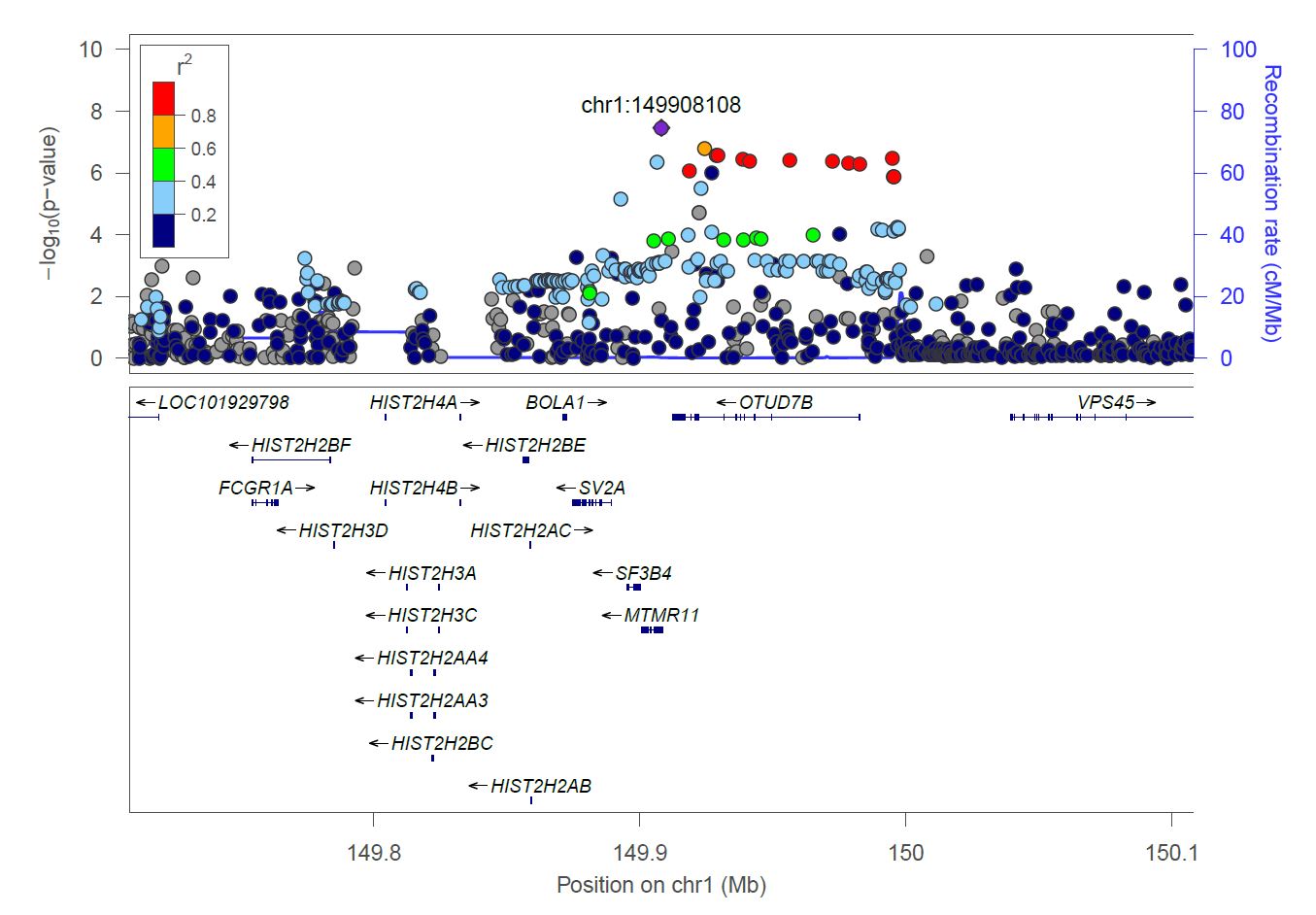
**

**1q21.2**

**
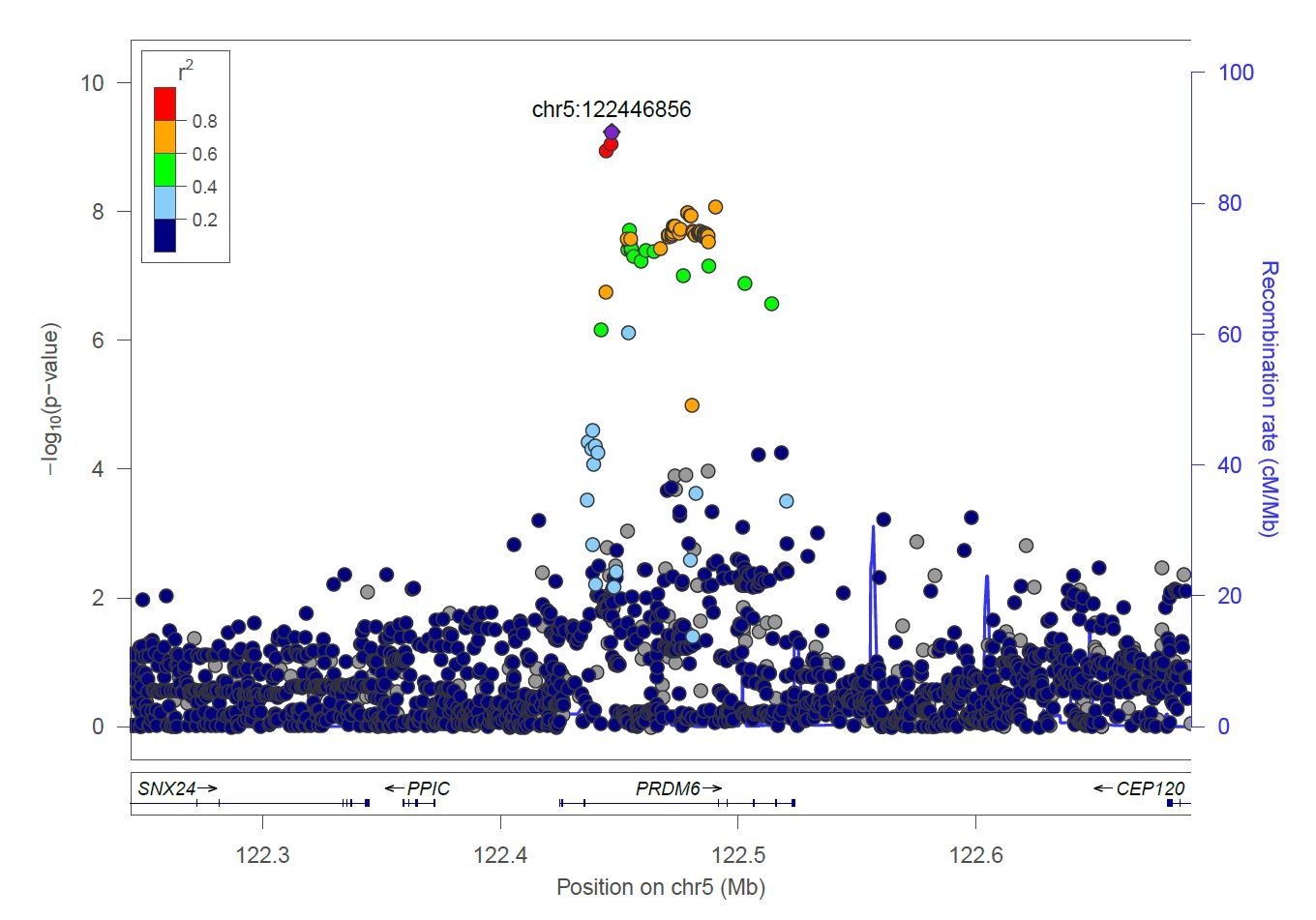
**

**5q23.2**

**
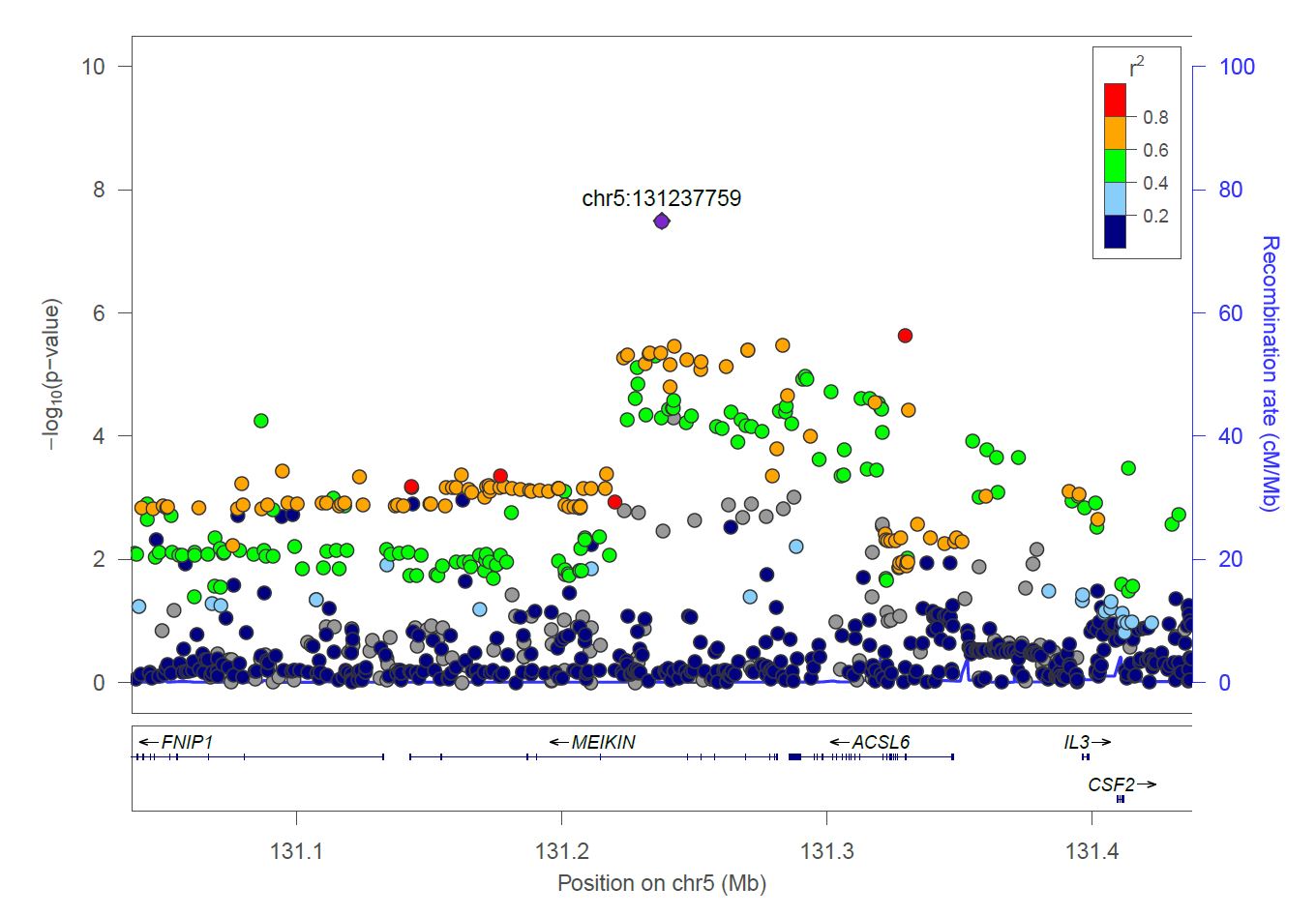
**

**5q31.1**

**
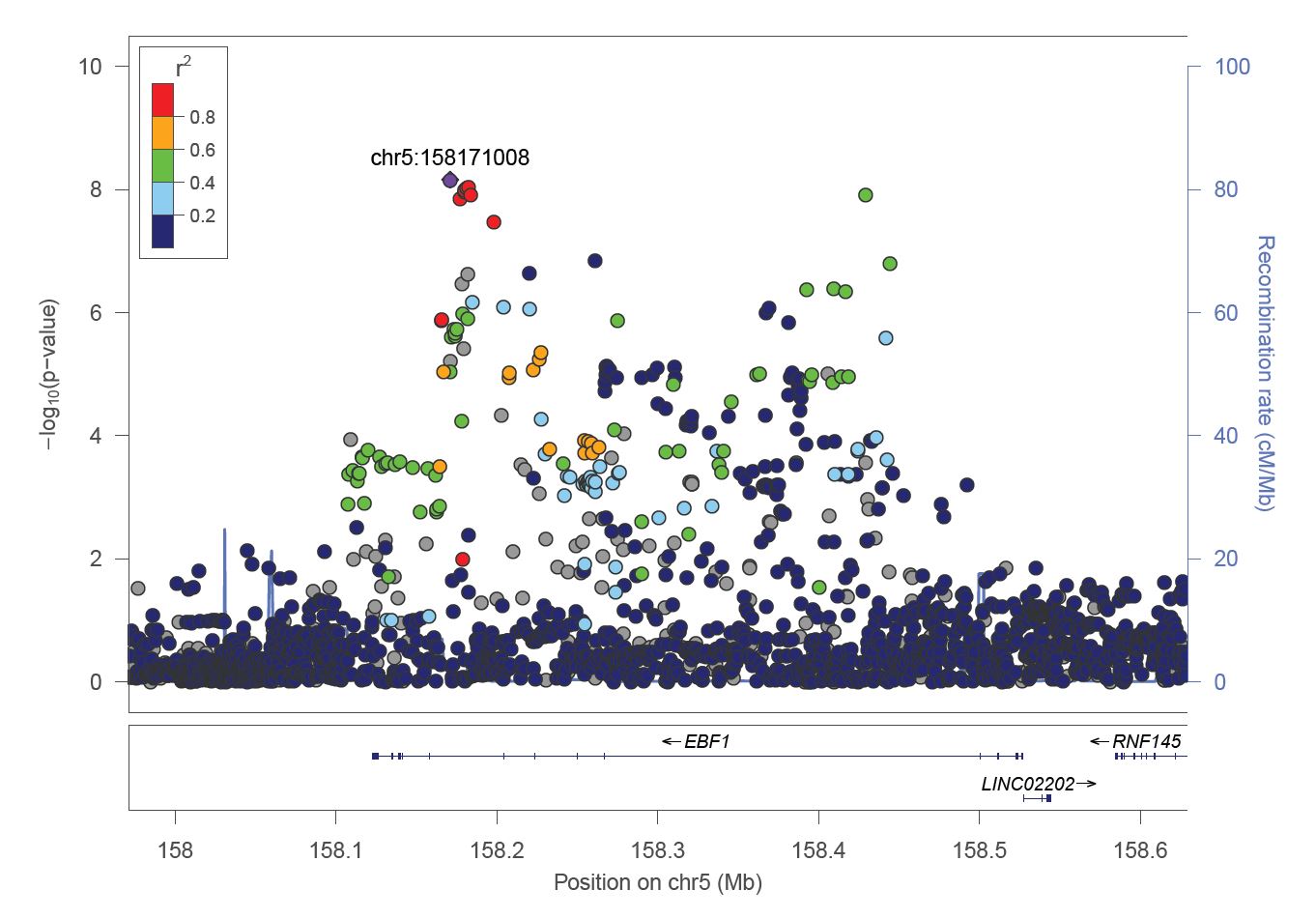
**

**5q33.3**

**
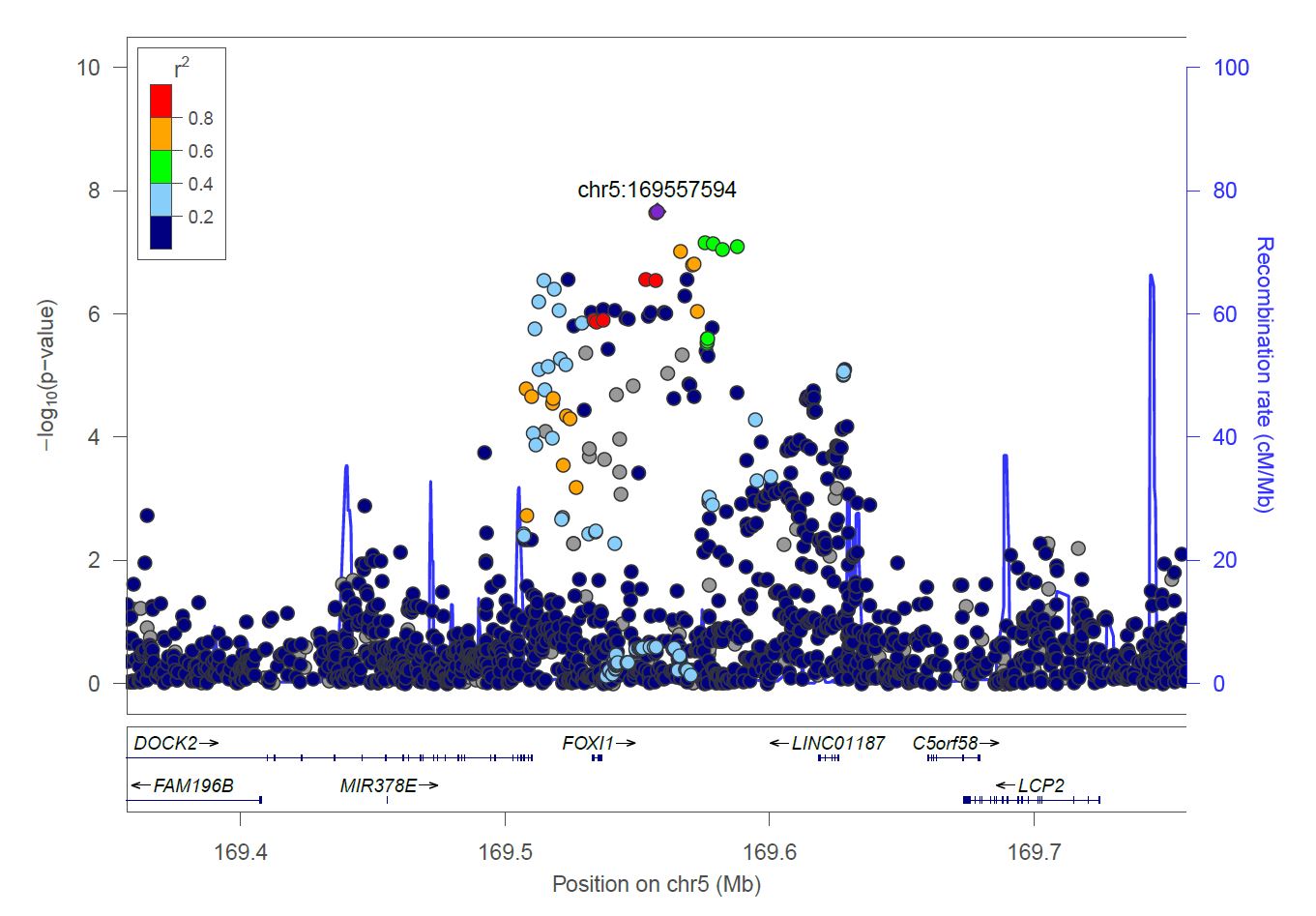
**

**5q35.1**

**
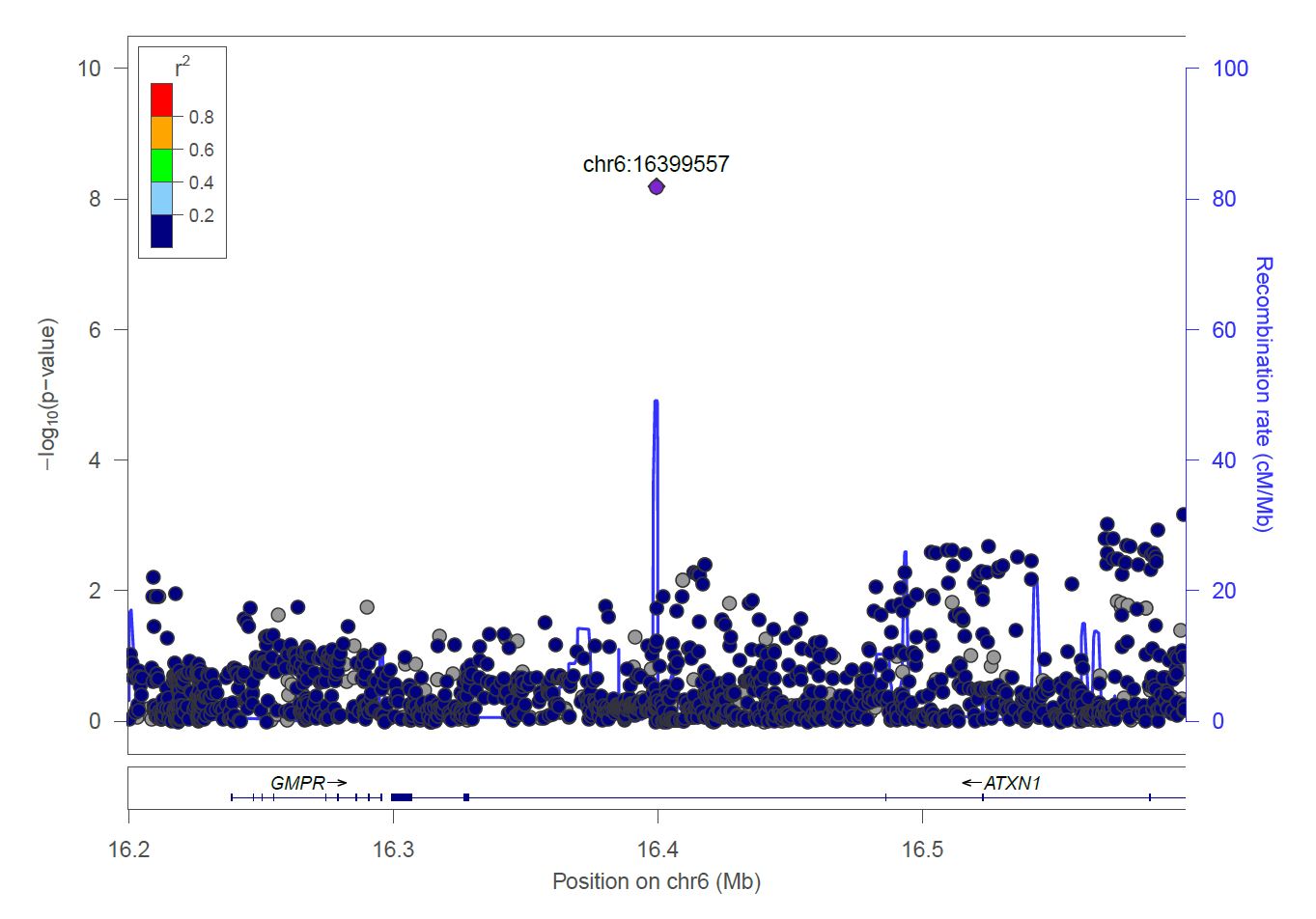
**

**6p22.3**

**
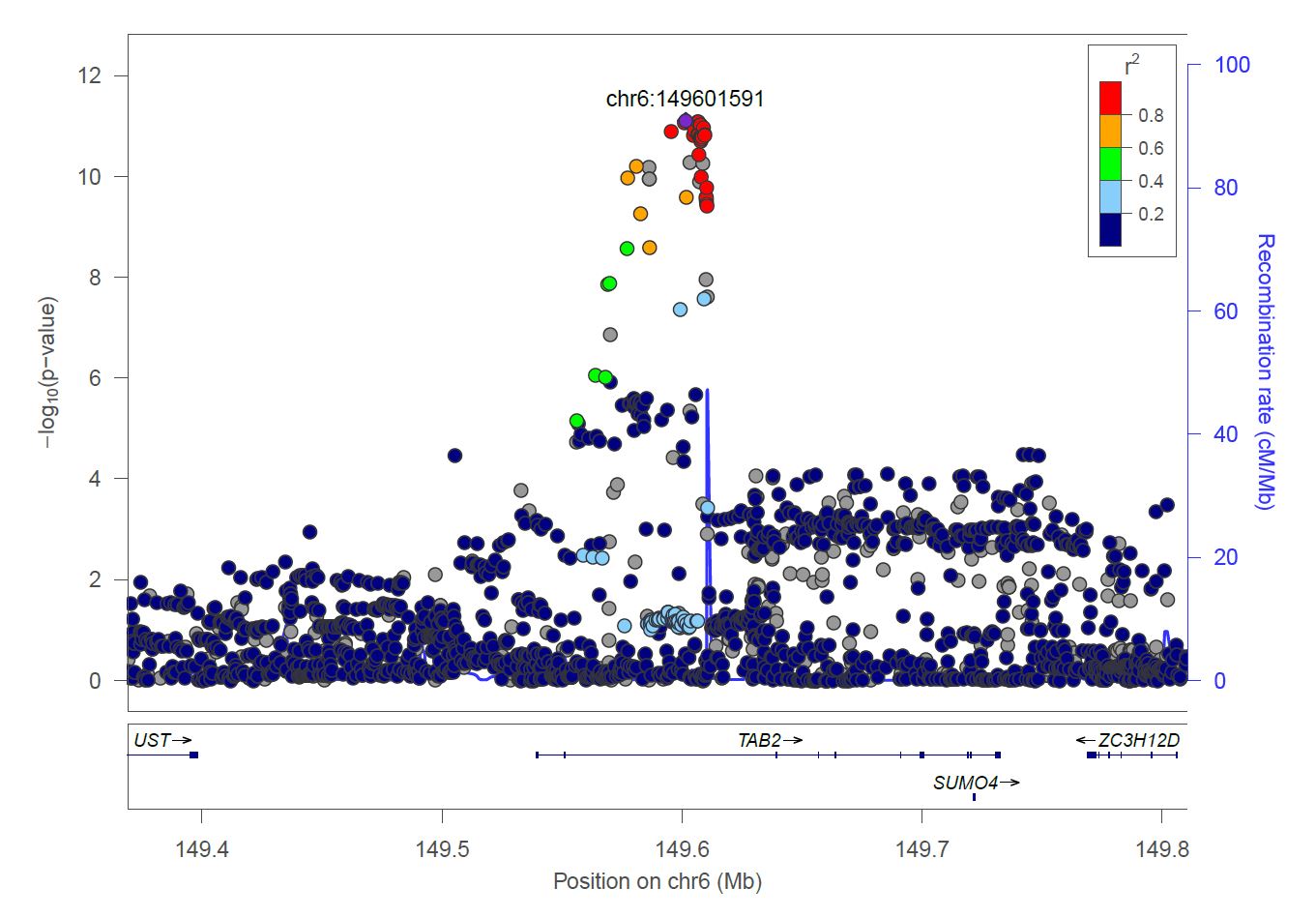
**

**6q25.1 (1)**

**
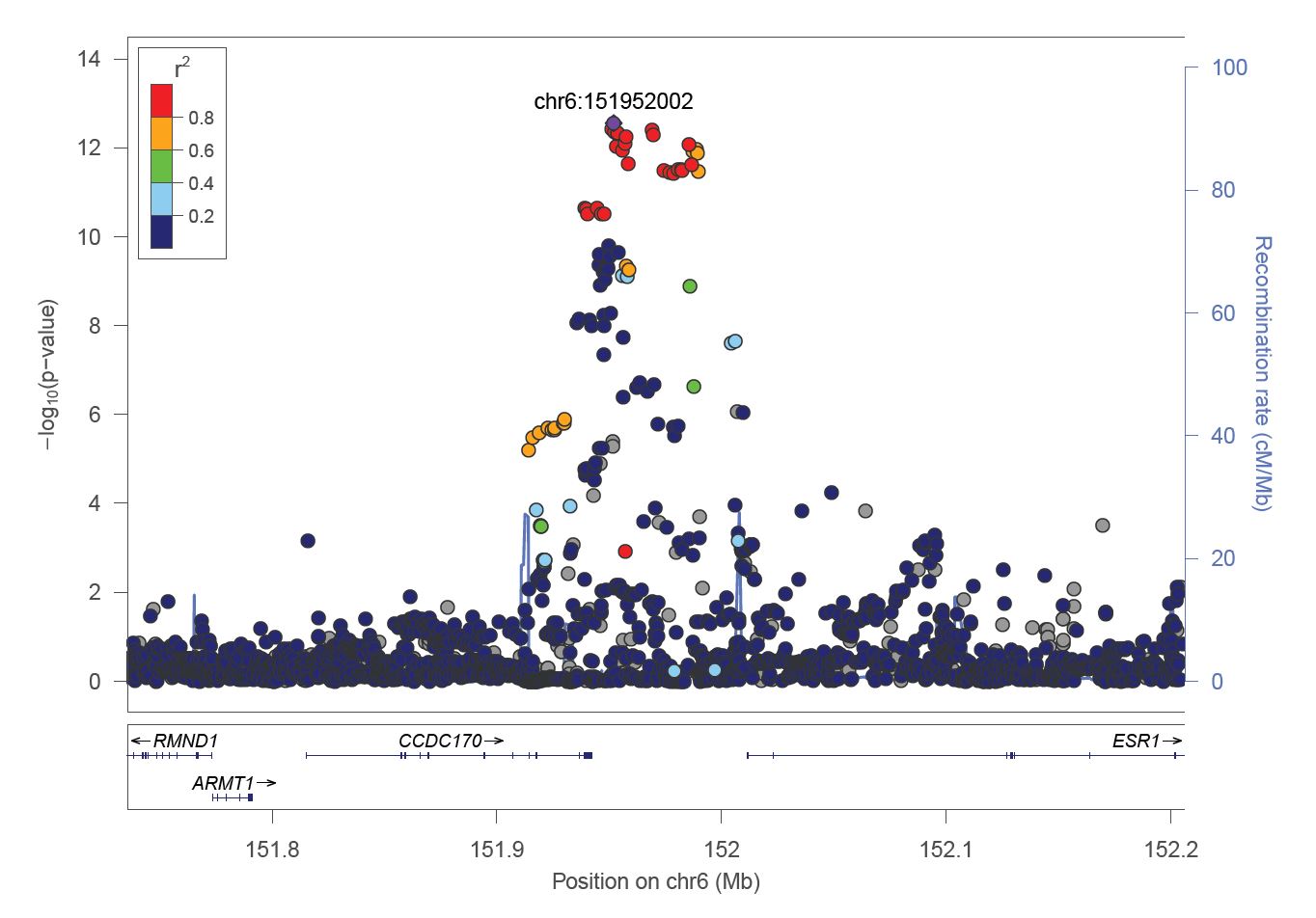
**

**6q25.1 (2)**

**
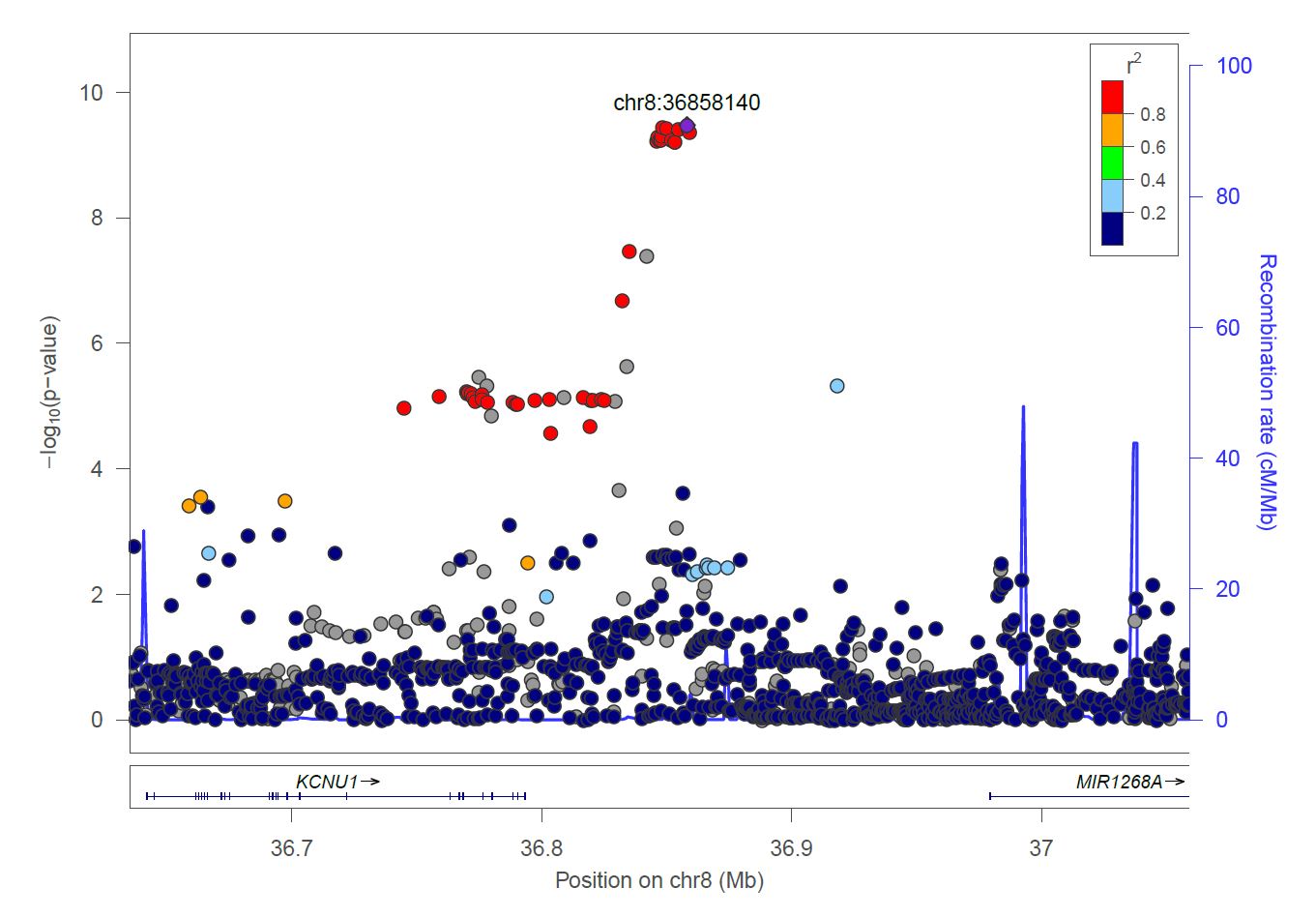
**

**8p11.23**

**
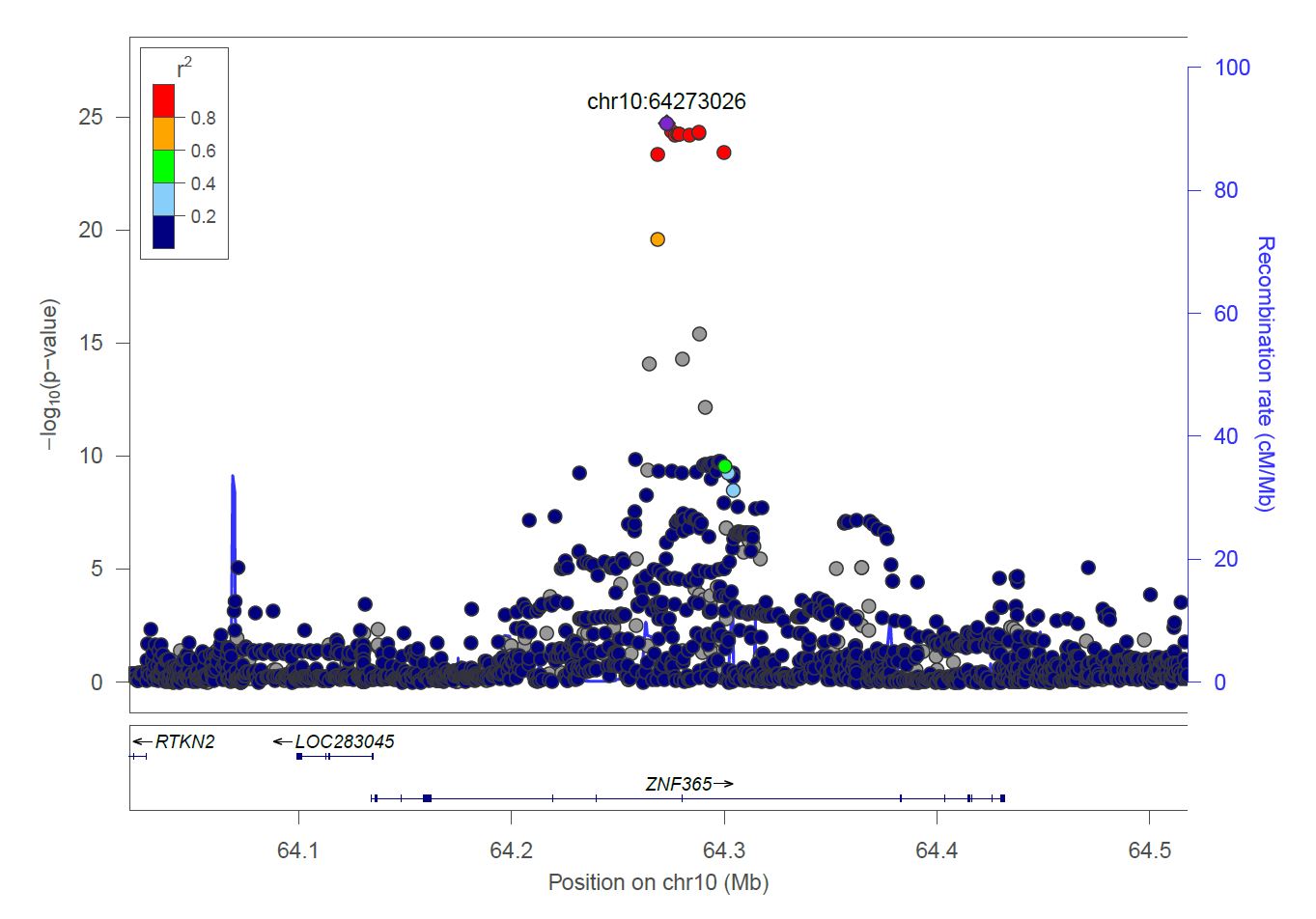
**

**10q21.2**

**
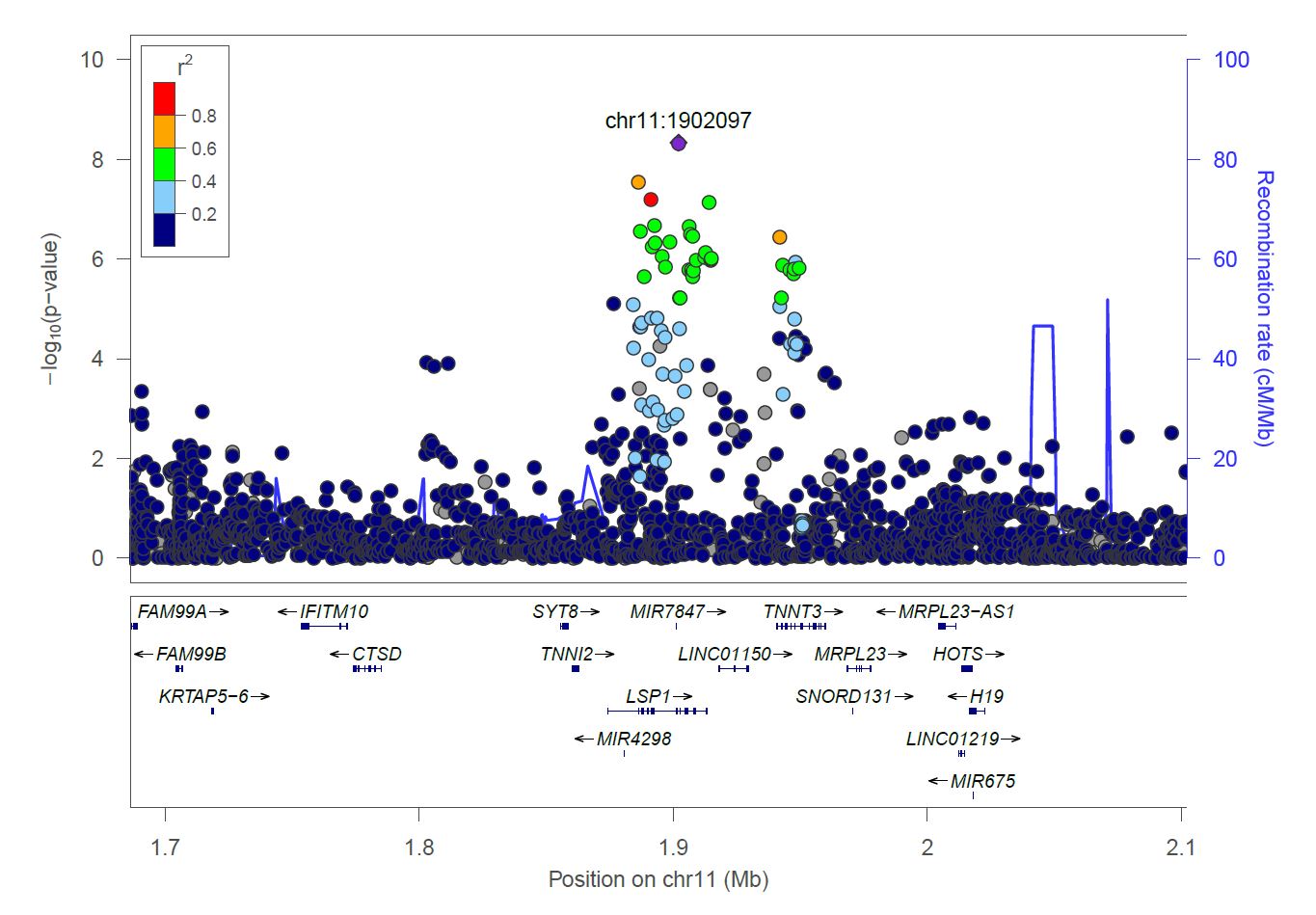
**

**11p15.5**

**
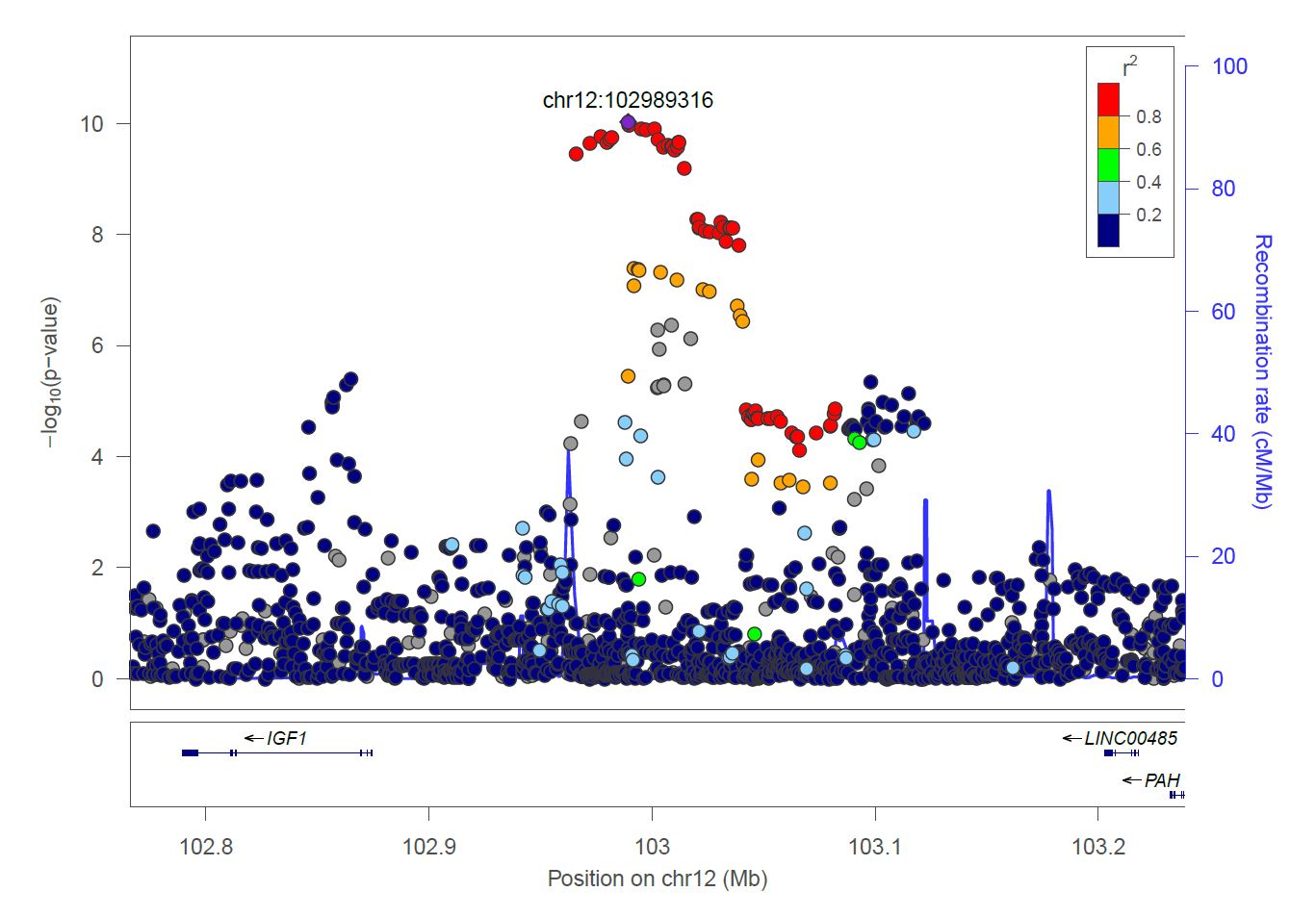
**

**12q23.2**

**
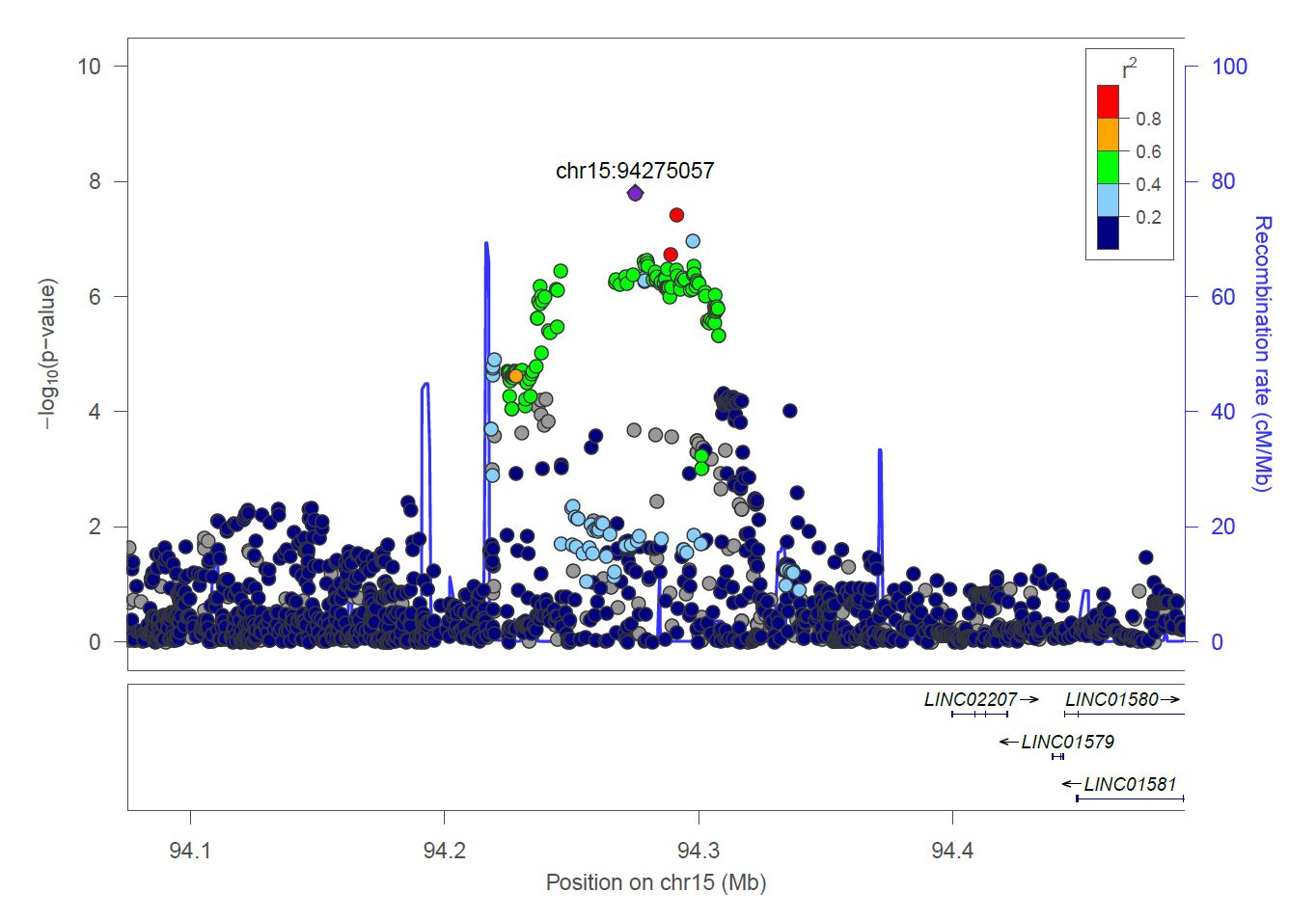
**

**15q26.1**

**
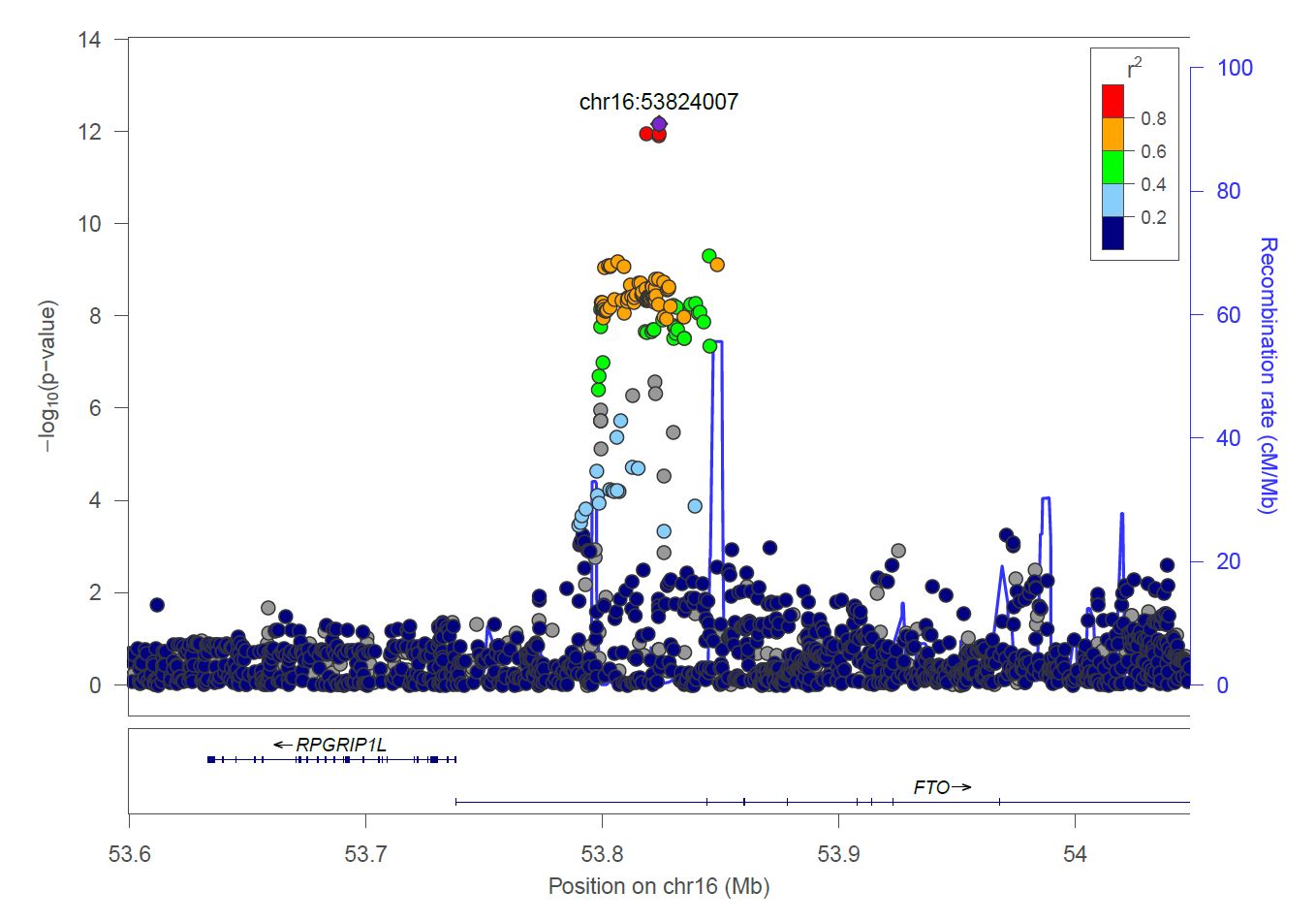
**

**16q12.2**

**
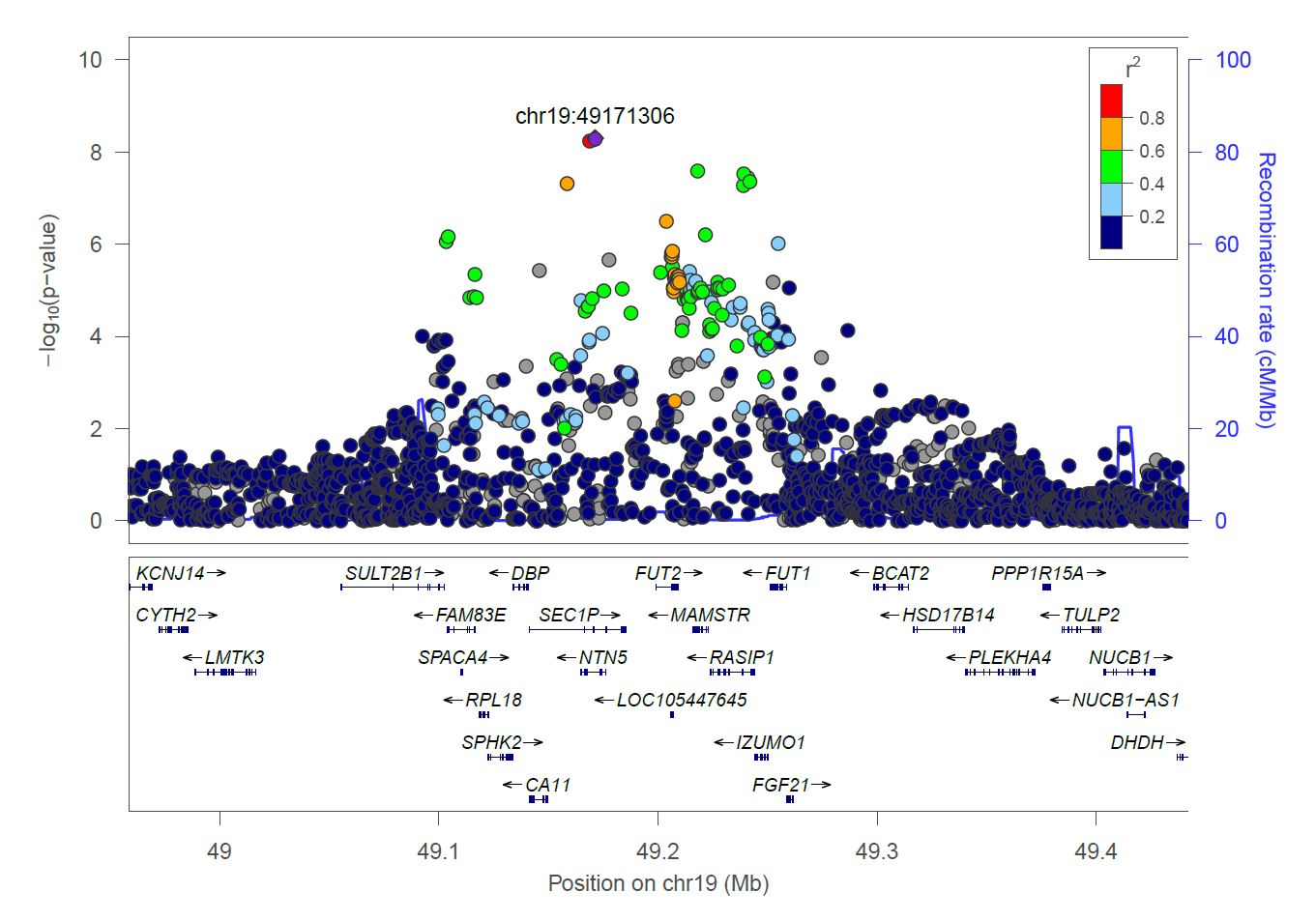
**

**19q13.33**

**Supplementary Figure 6.** Forest plots presenting the GWAS meta-analysis results of the lead SNP of eighteen genome-wide significant loci of DA. In each plot, the study name, sample size, beta estimate and 95% confidence interval for each participating study is shown. The associations shown were adjusted for age and BMI at mammogram as well as the first ten ancestry informative principal components. Studies with less than 100 individuals are not shown on the forest plots, although they were still included in the meta-analysis.


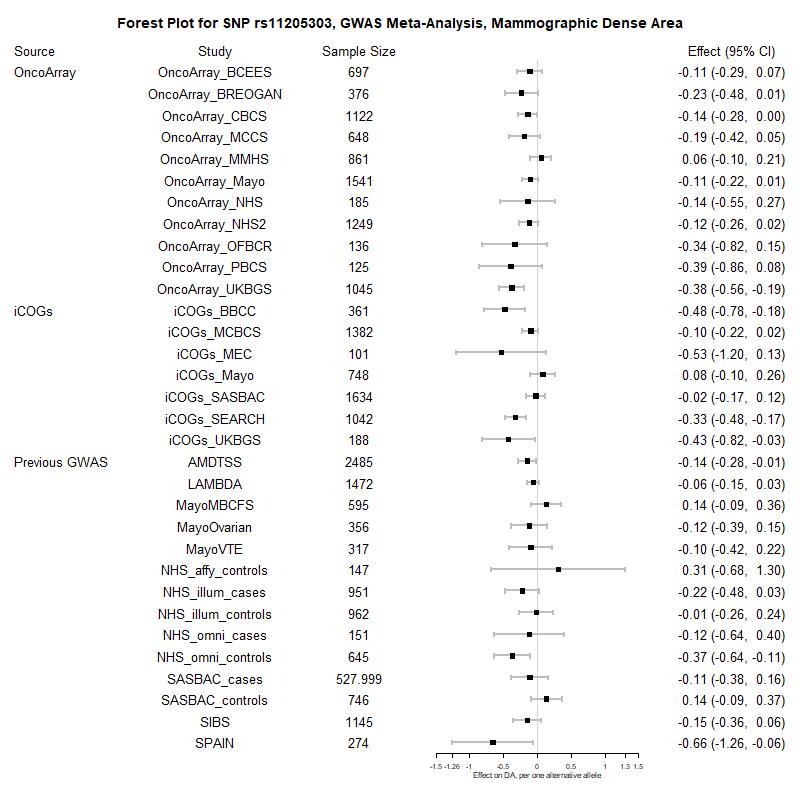


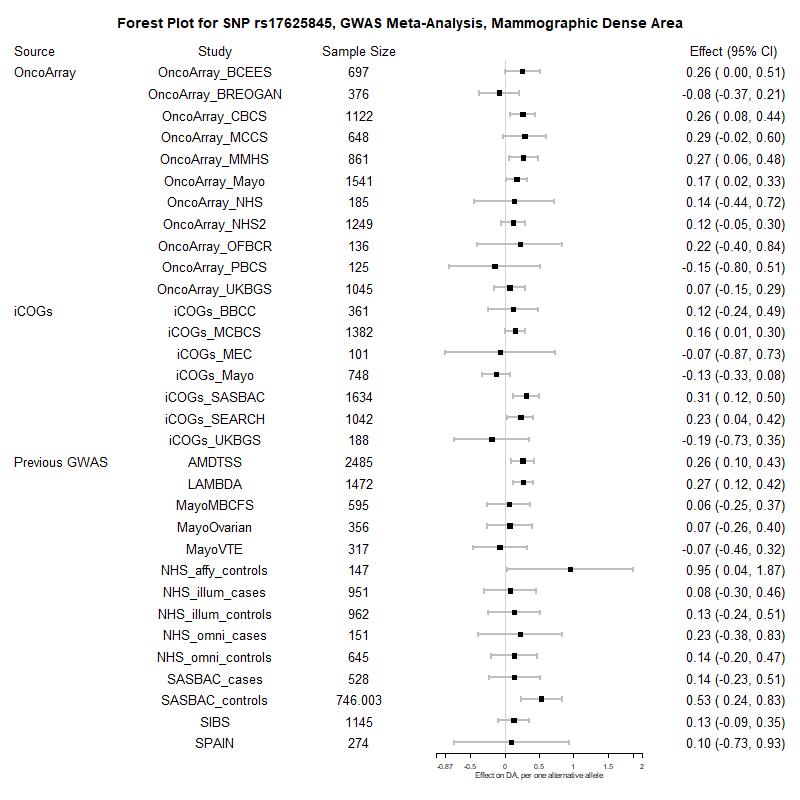

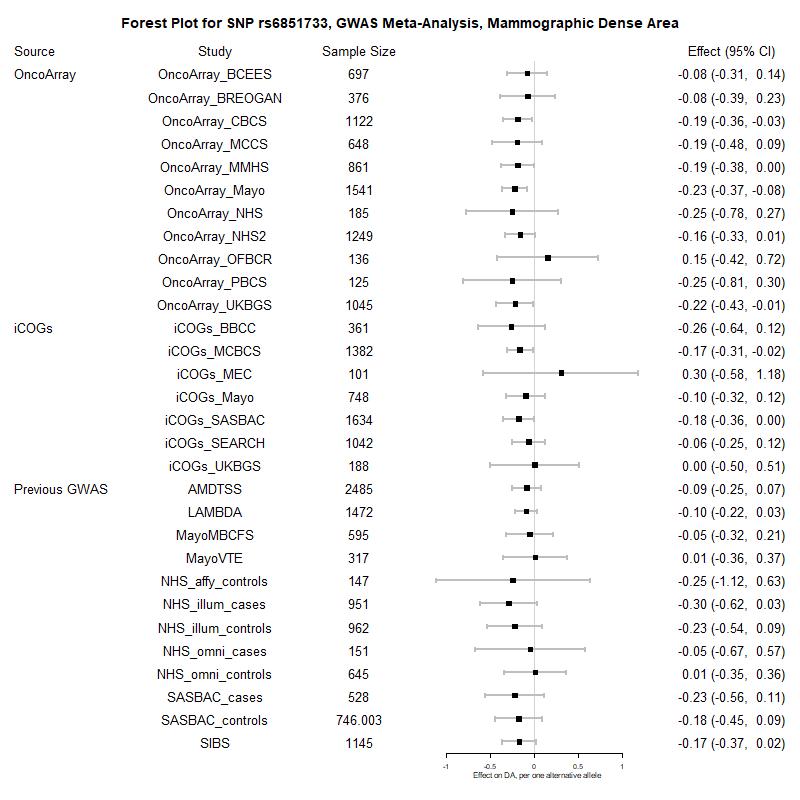

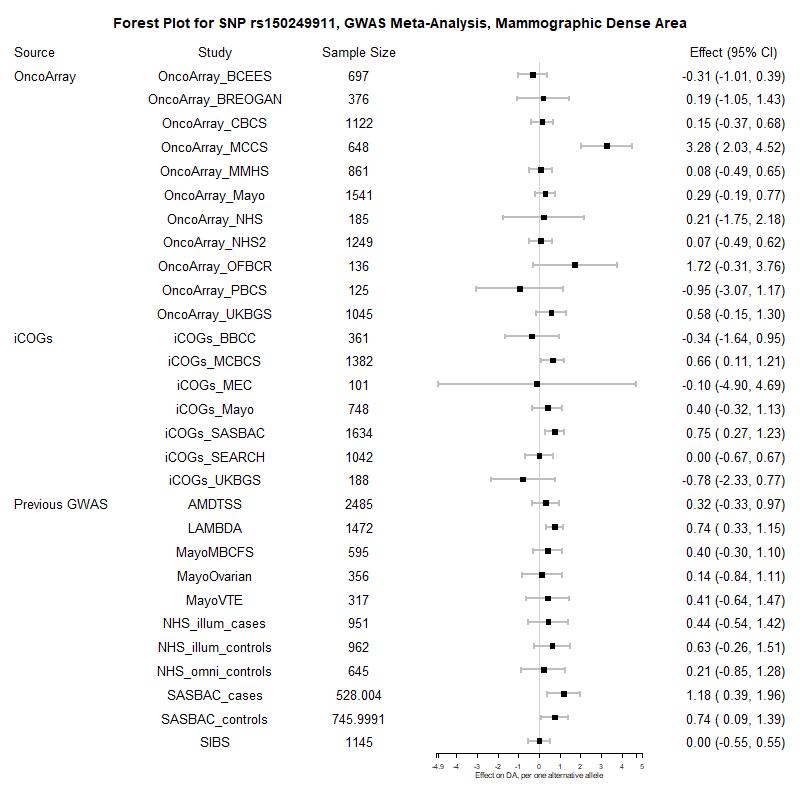

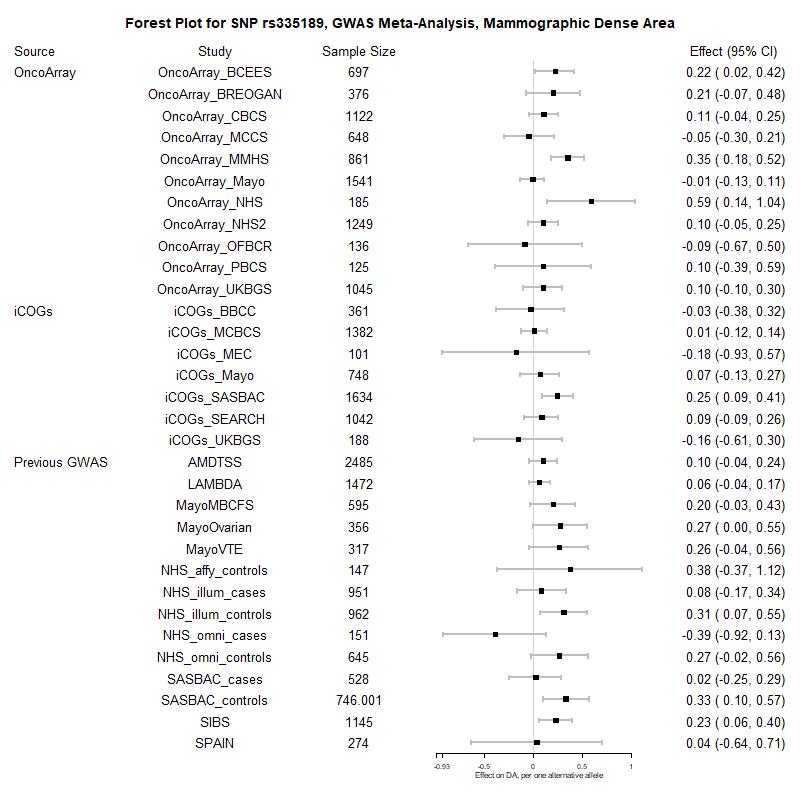

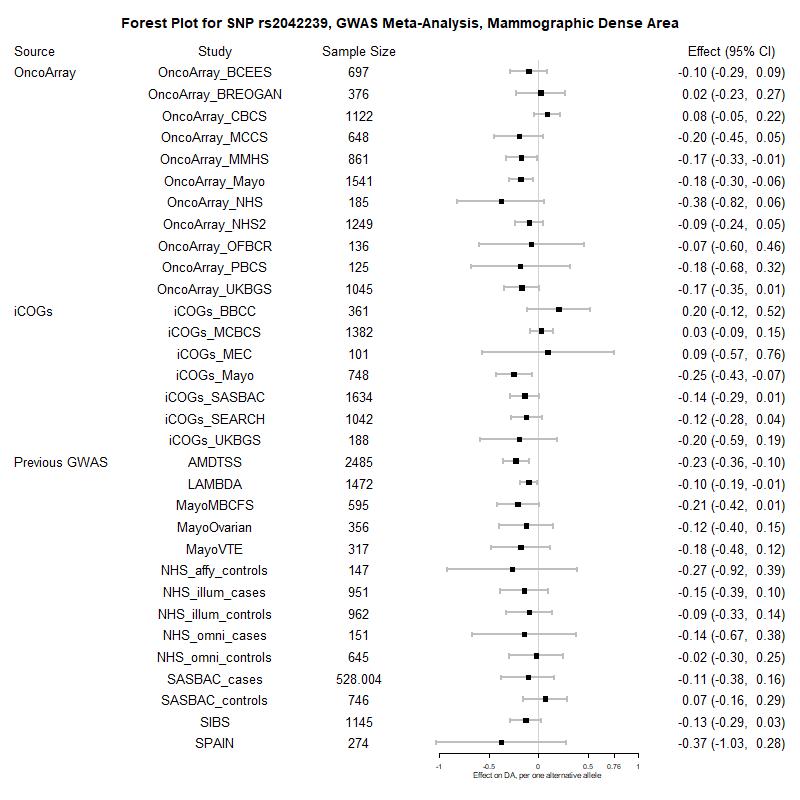

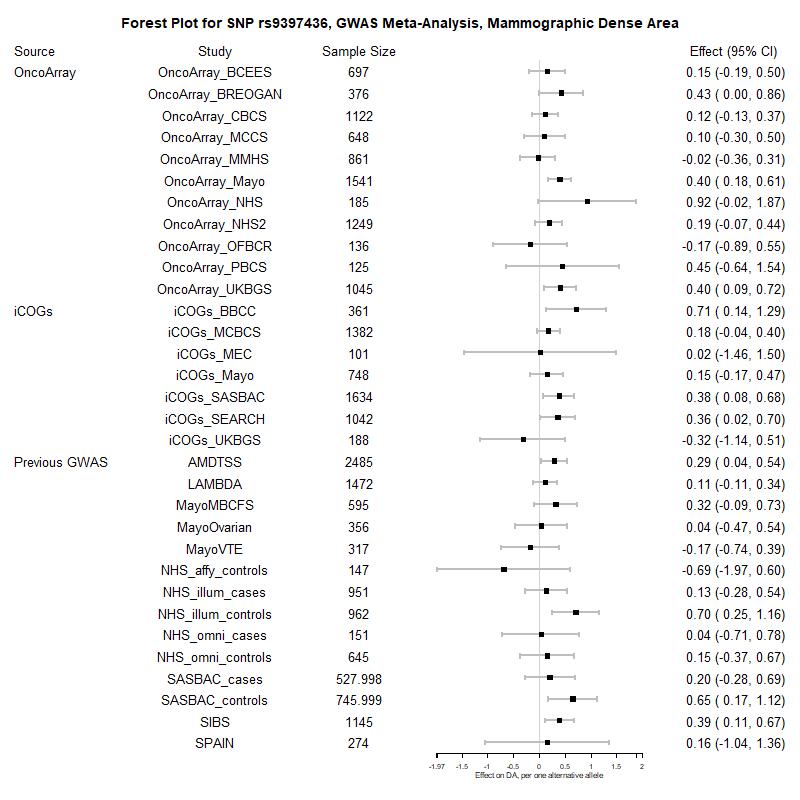

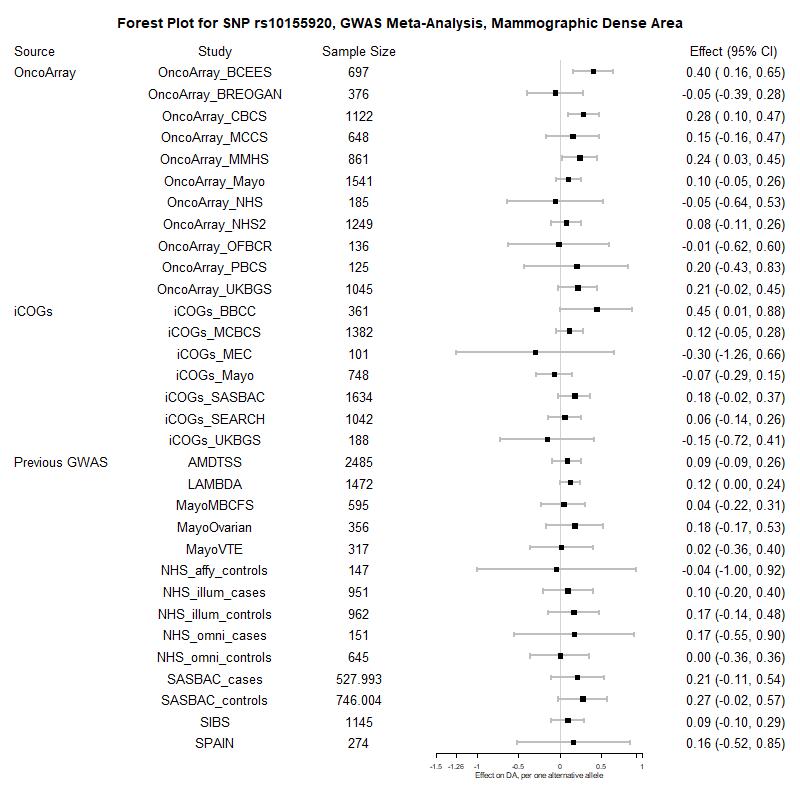

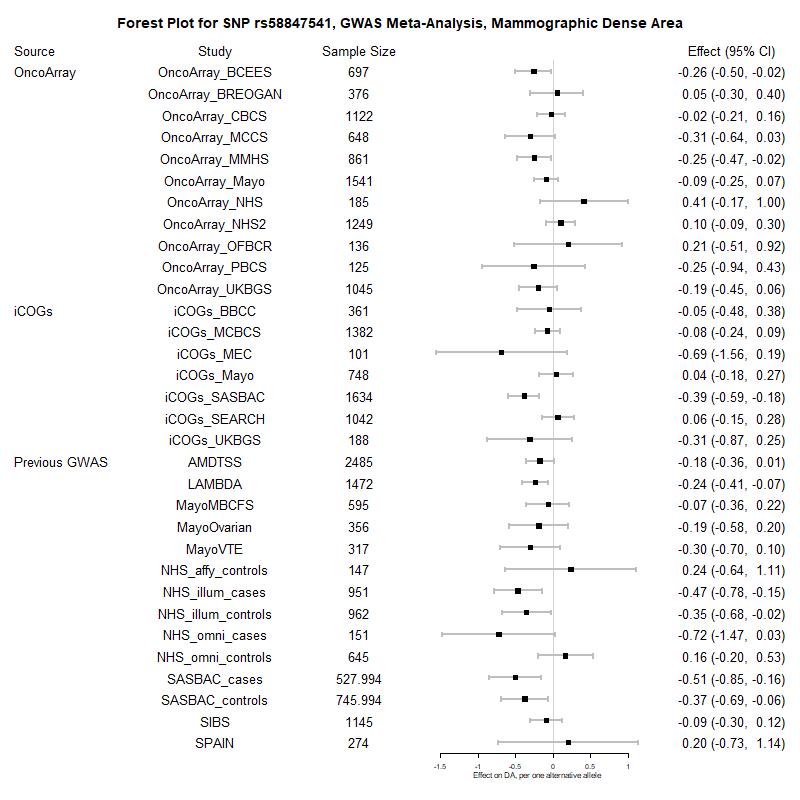

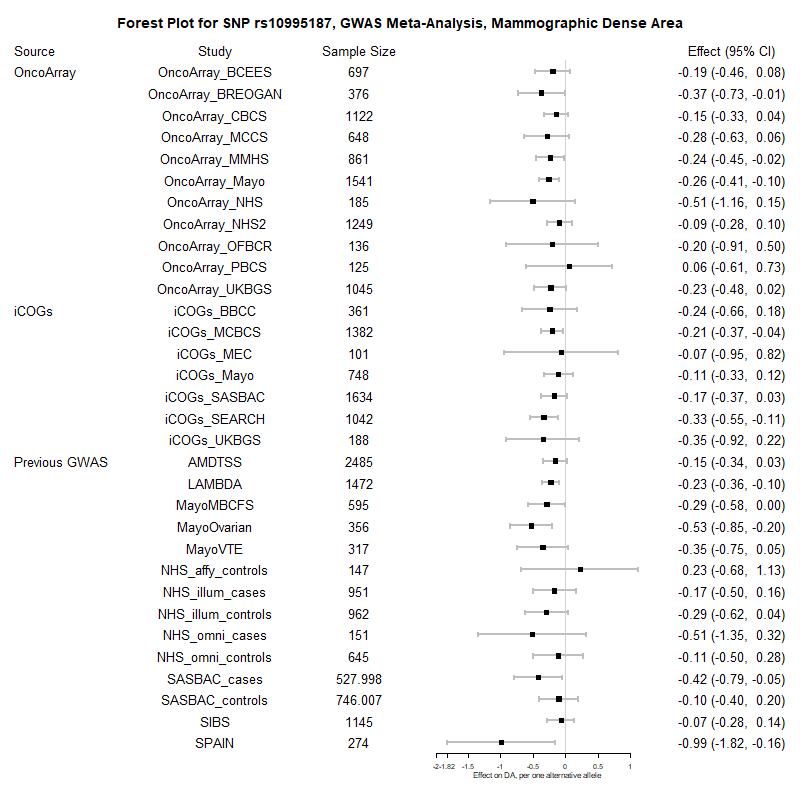

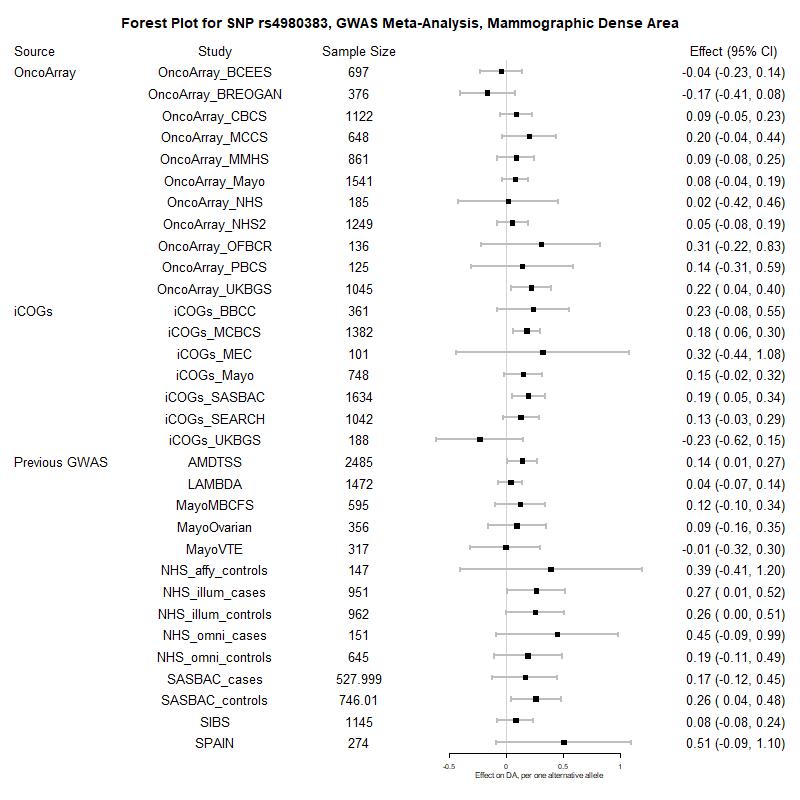

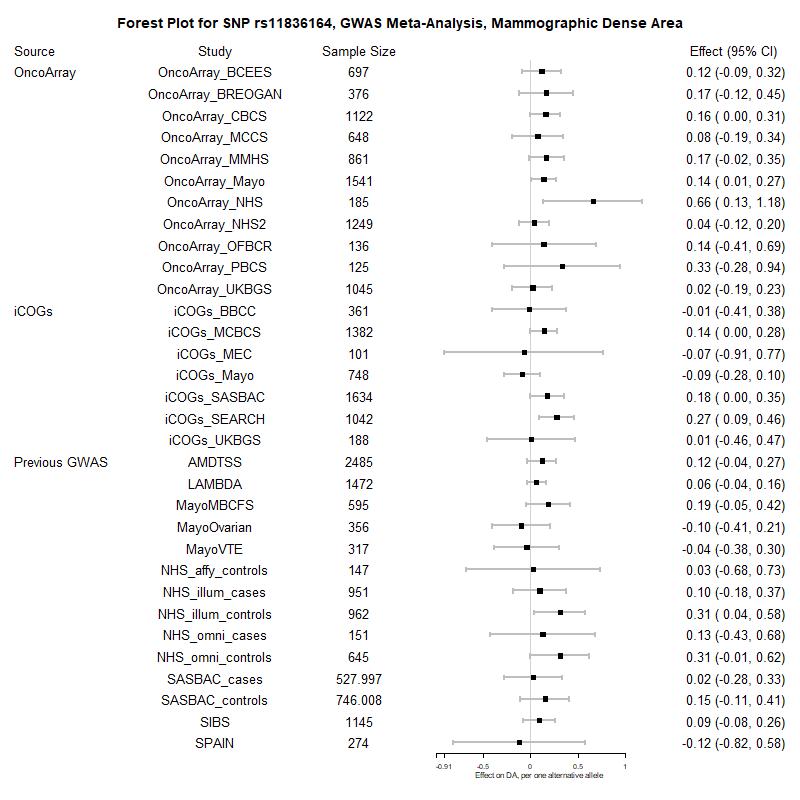

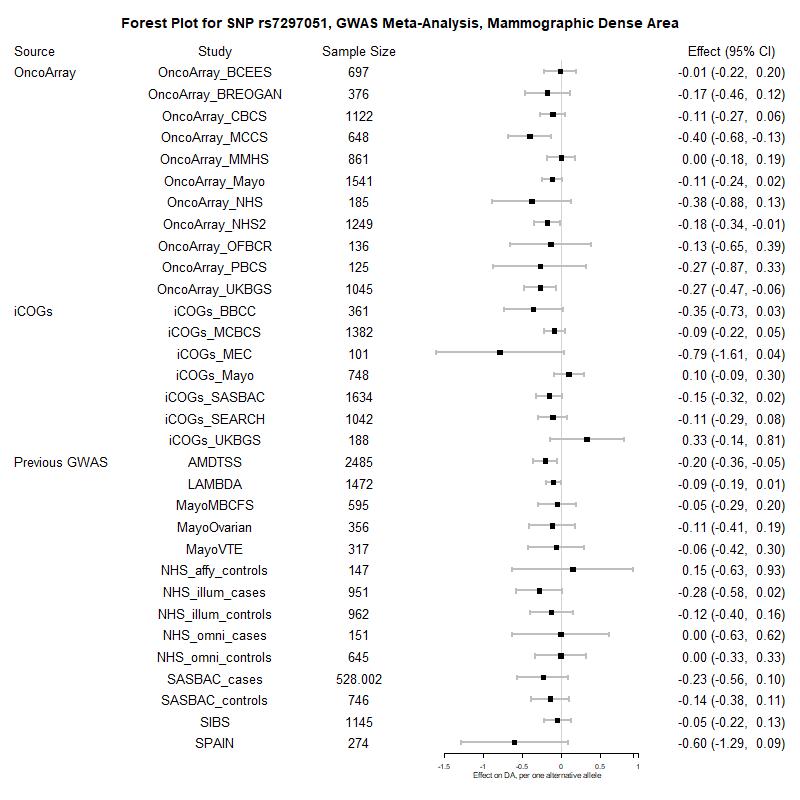

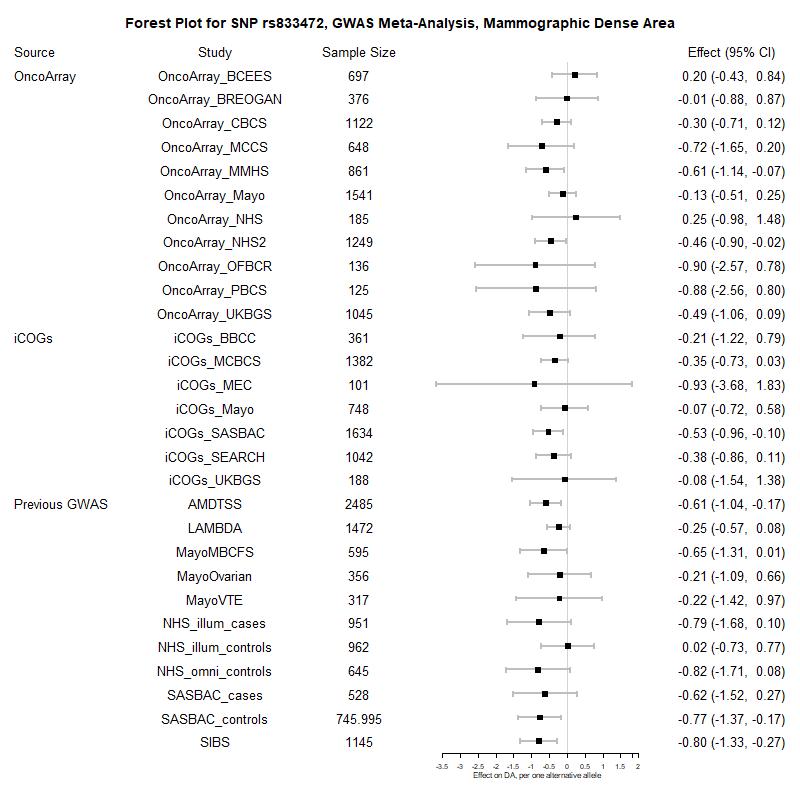

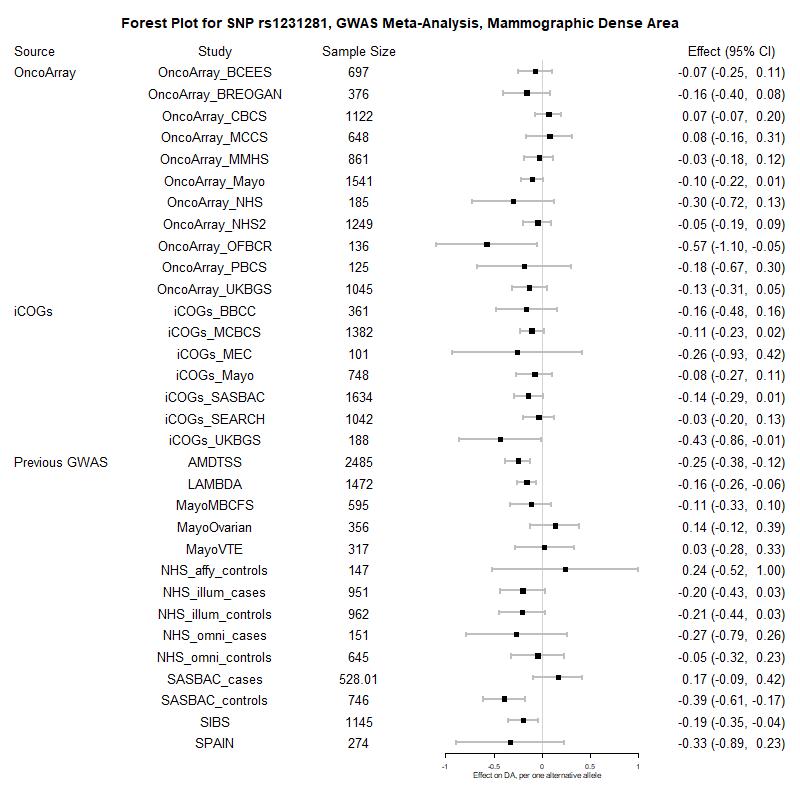

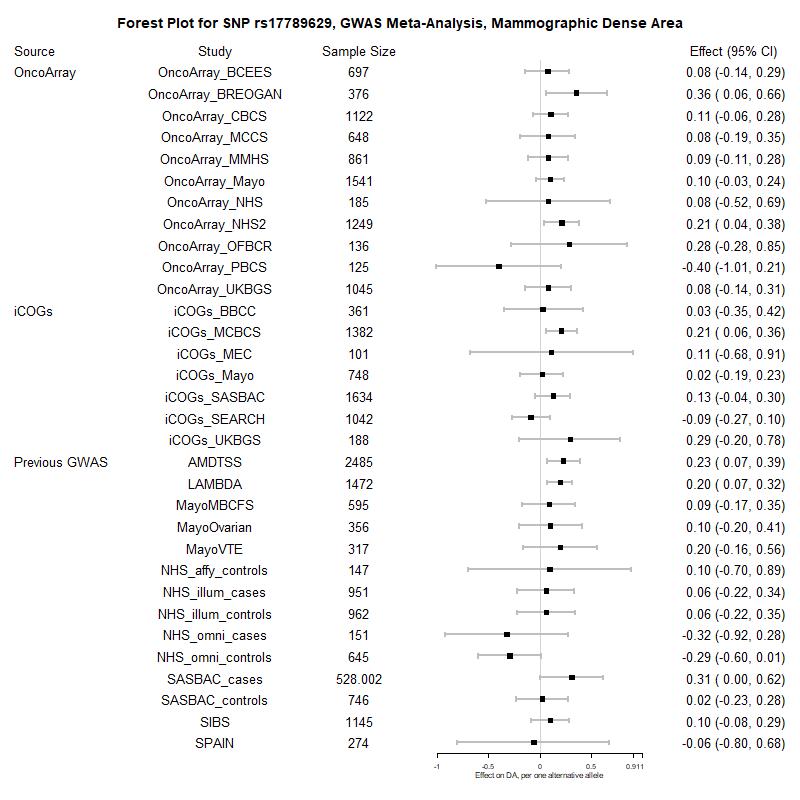

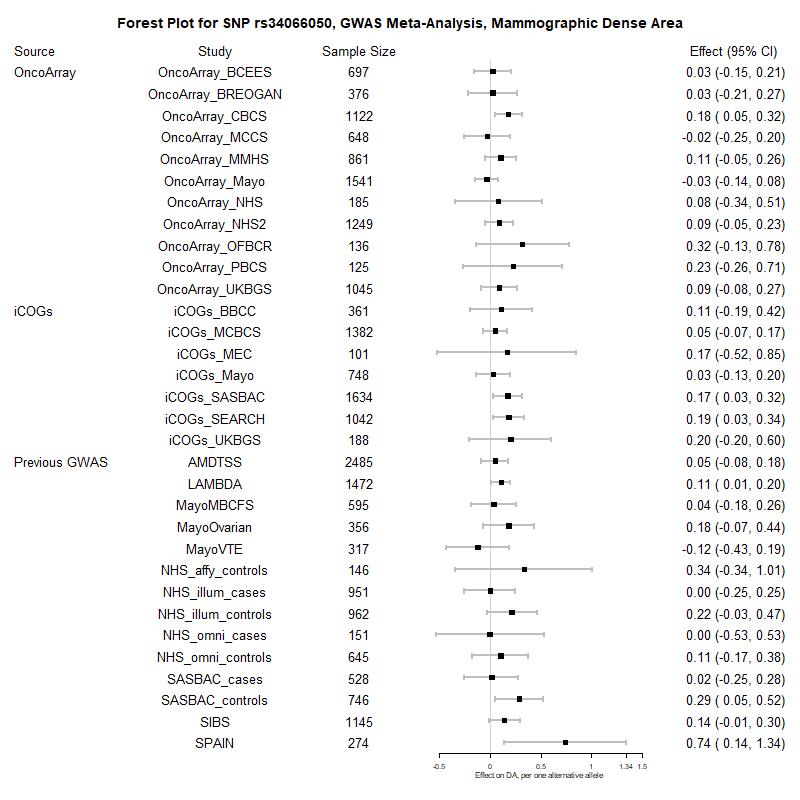

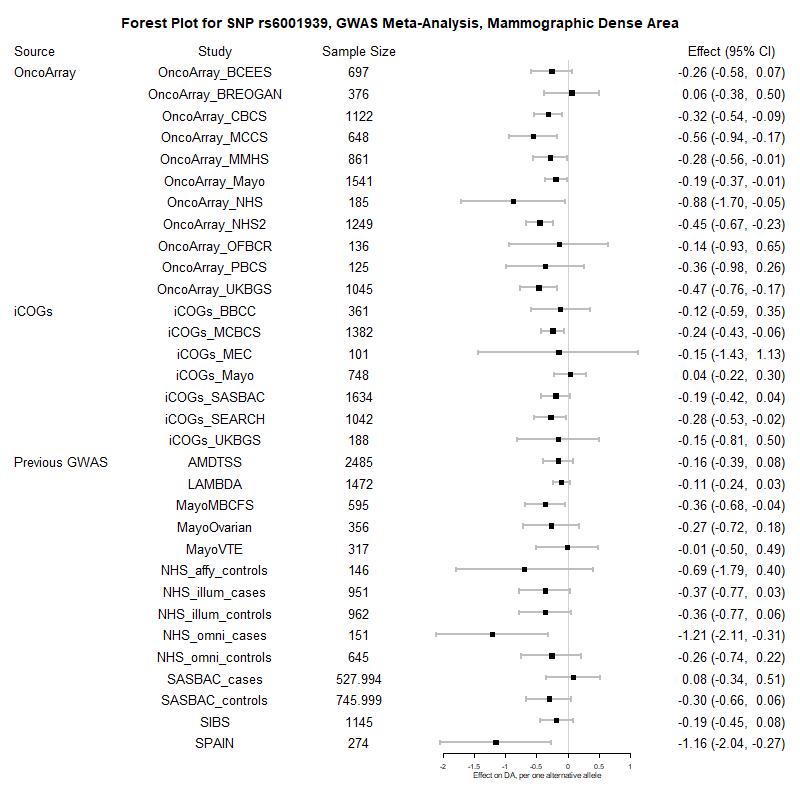


**Supplementary Figure 7.** Forest plots presenting the GWAS meta-analysis results of the lead SNP of six genome-wide significant loci of NDA. In each plot, the study name, sample size, beta estimate and 95% confidence interval for each participating study is shown. The associations shown were adjusted for age and BMI at mammogram as well as the first ten ancestry informative principal components. Studies with less than 100 individuals are not shown on the forest plots, although they were still included in the meta-analysis.


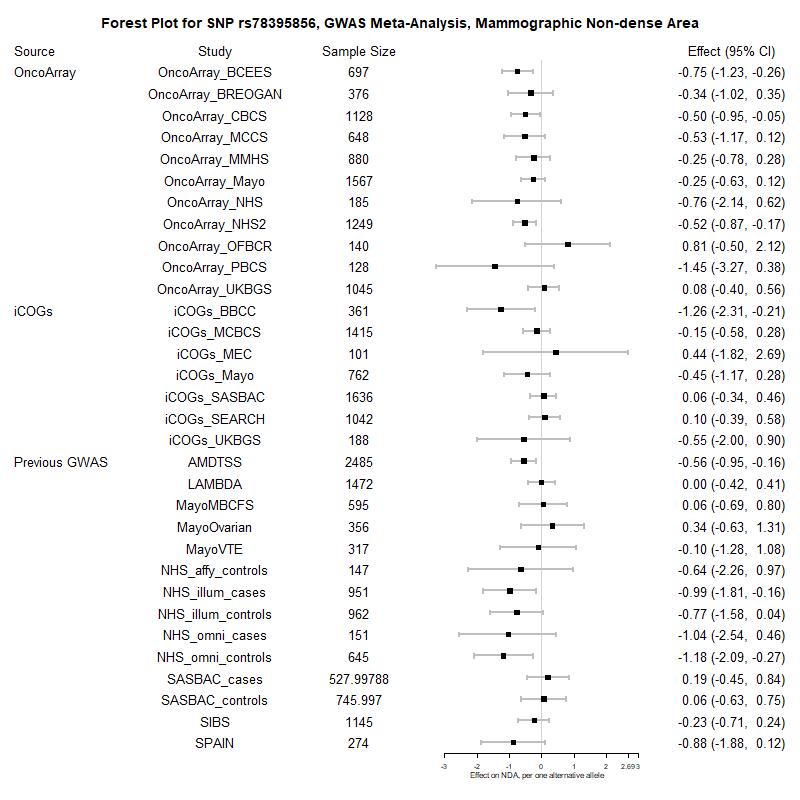


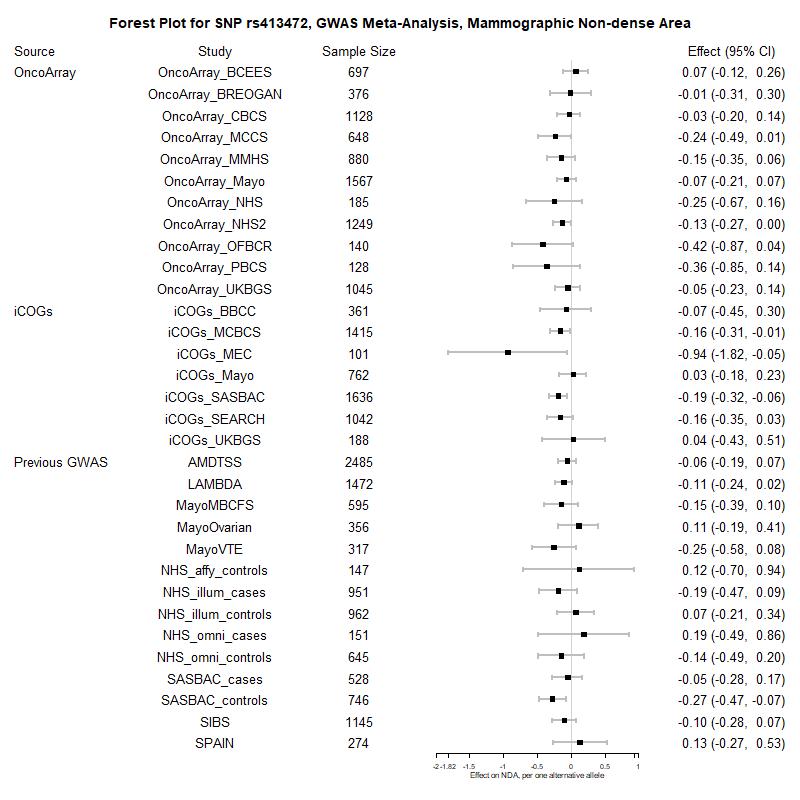

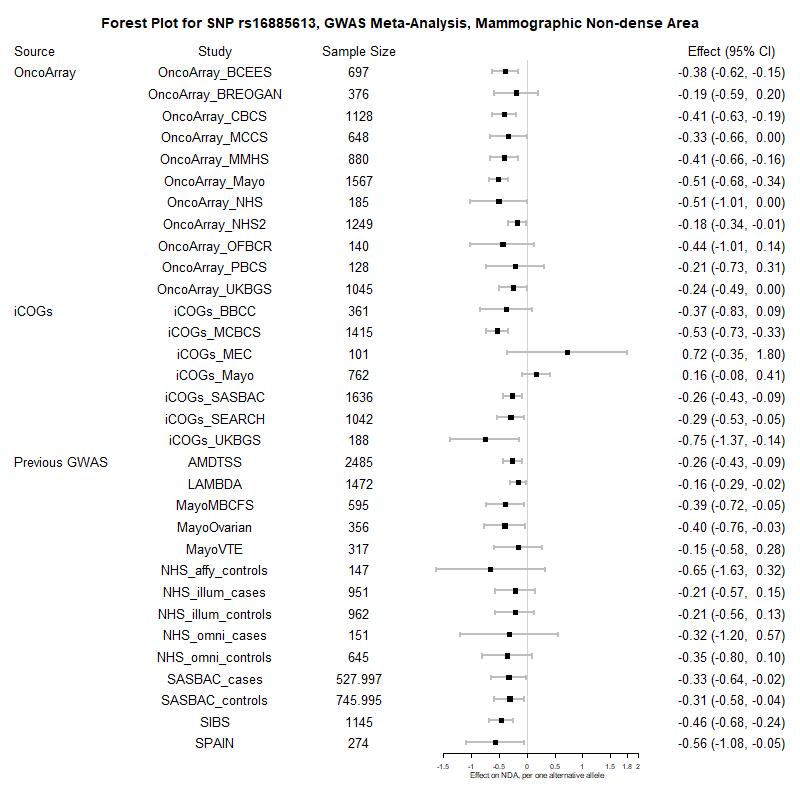

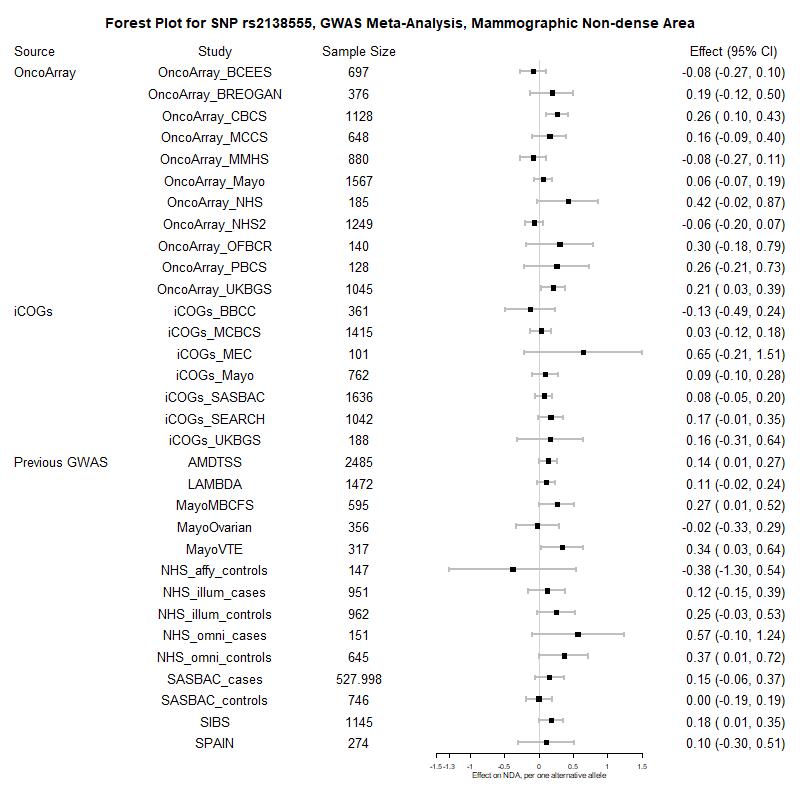

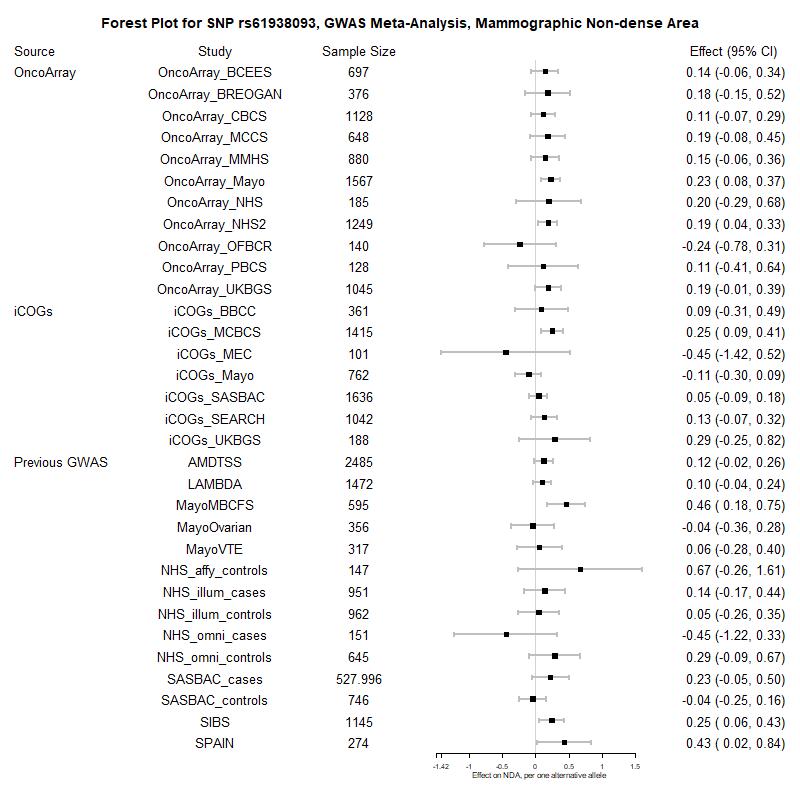

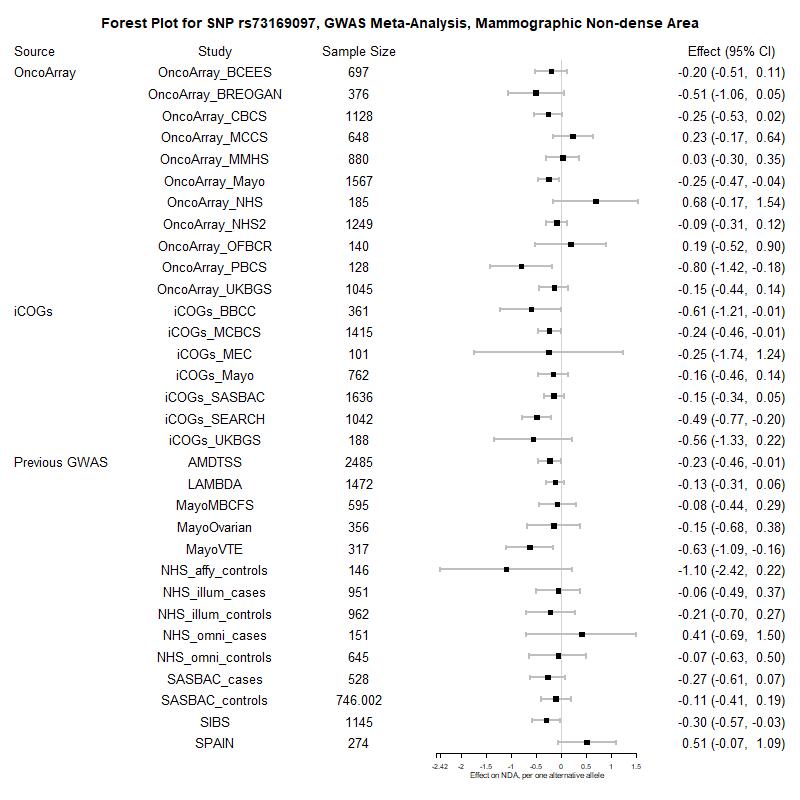


**Supplementary Figure 8.** Forest plots presenting the GWAS meta-analysis results of the lead SNP of fifteen genome-wide significant loci of PMD. In each plot, the study name, sample size, beta estimate and 95% confidence interval for each participating study is shown. The associations shown were adjusted for age and BMI at mammogram as well as the first ten ancestry informative principal components. Studies with less than 100 individuals are not shown on the forest plots, although they were still included in the meta-analysis.


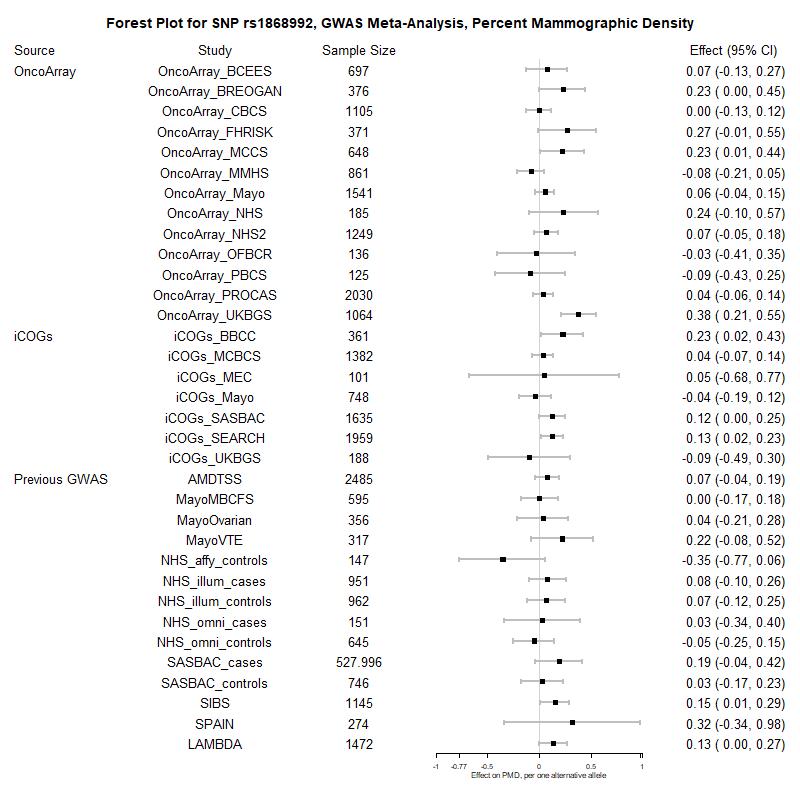


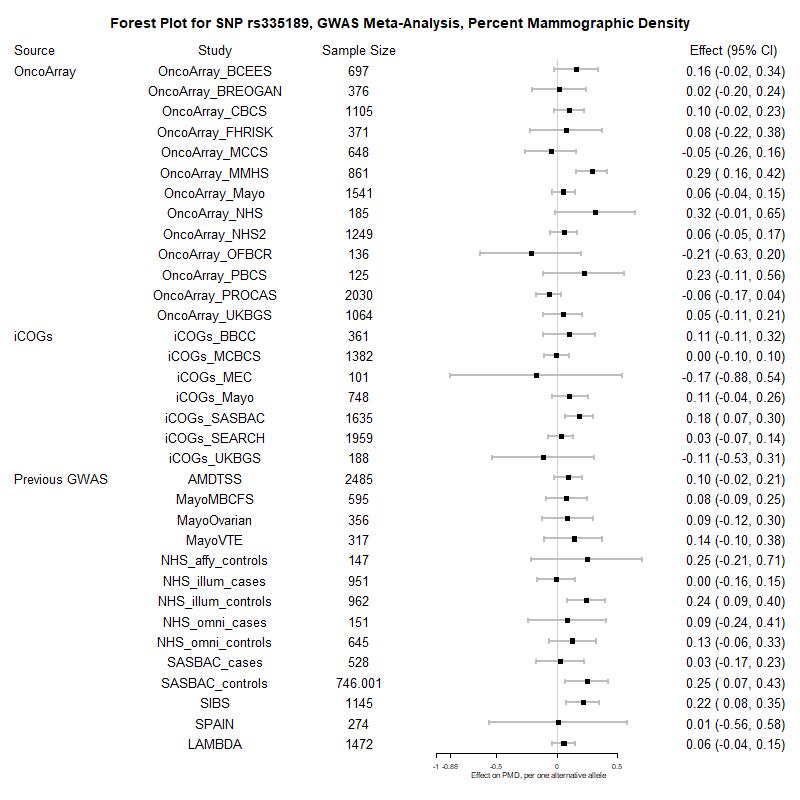


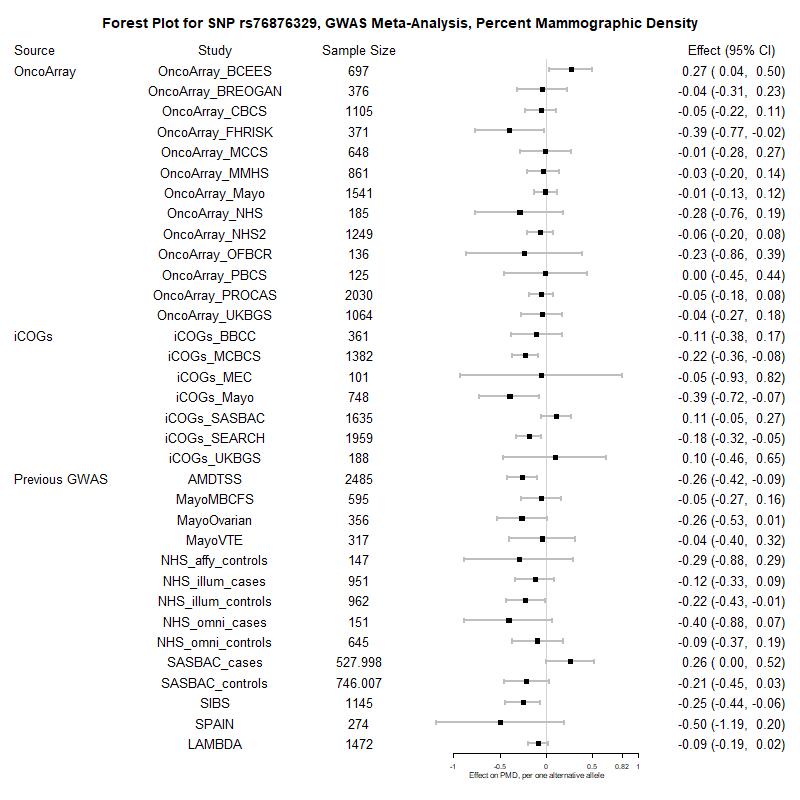


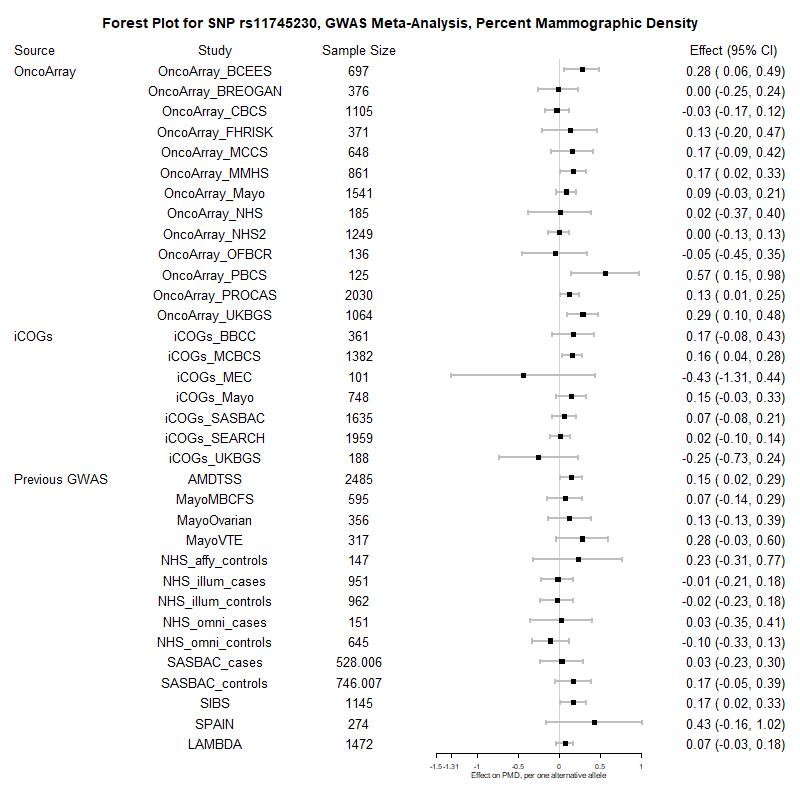


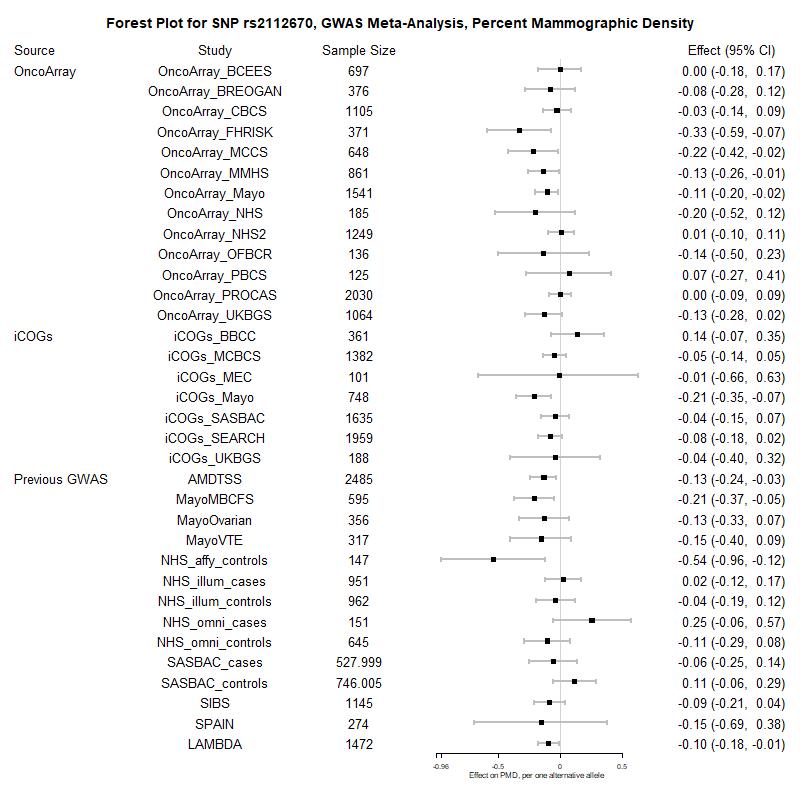


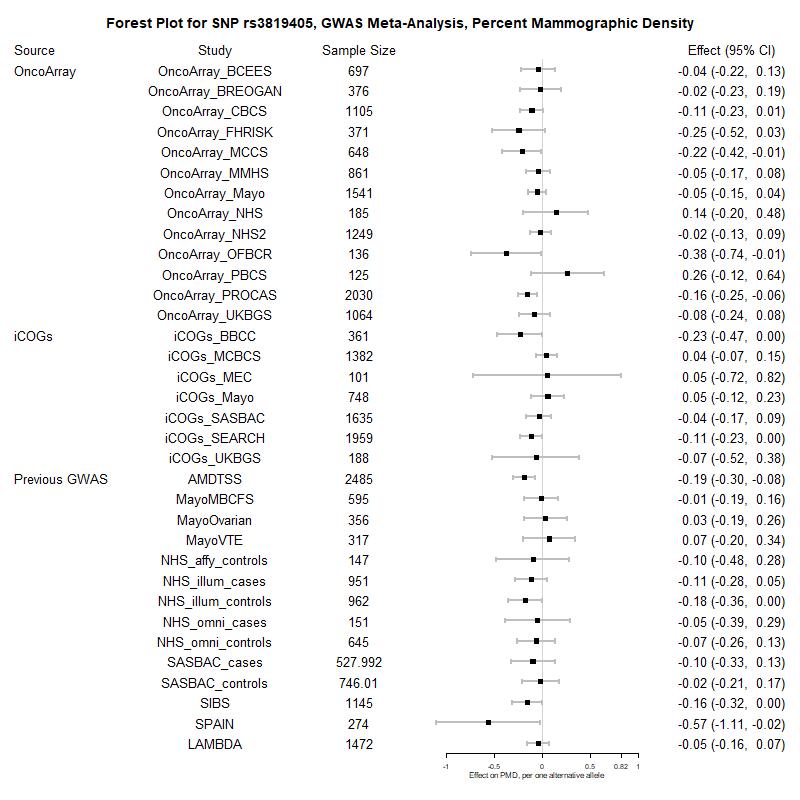


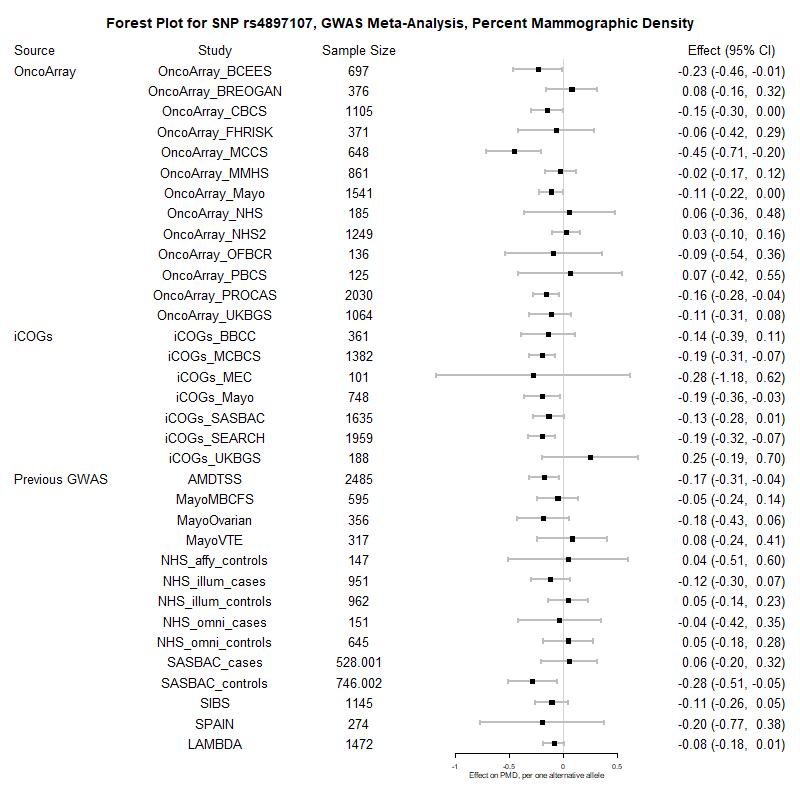


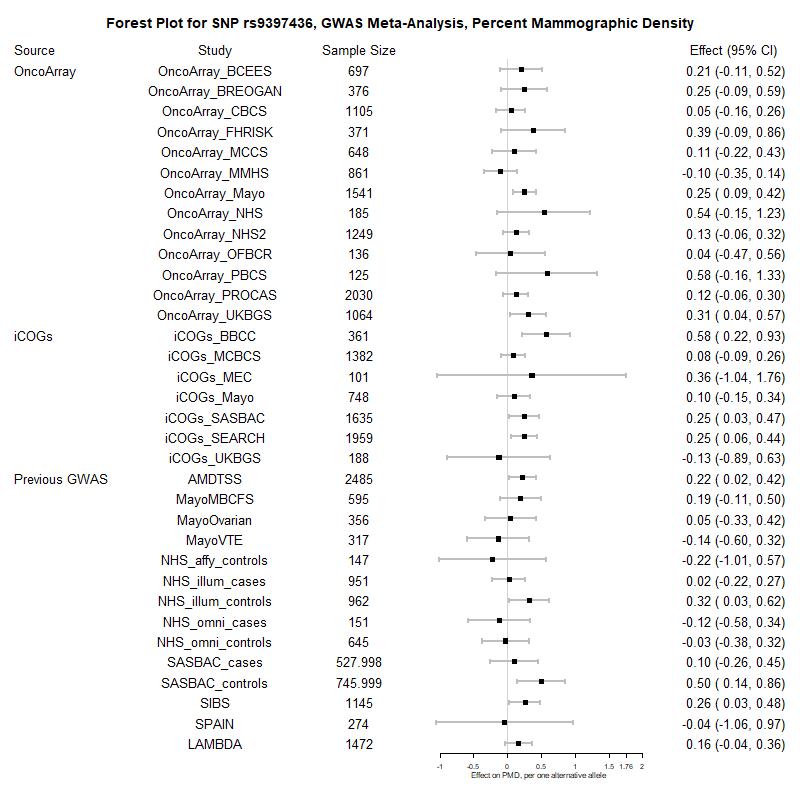


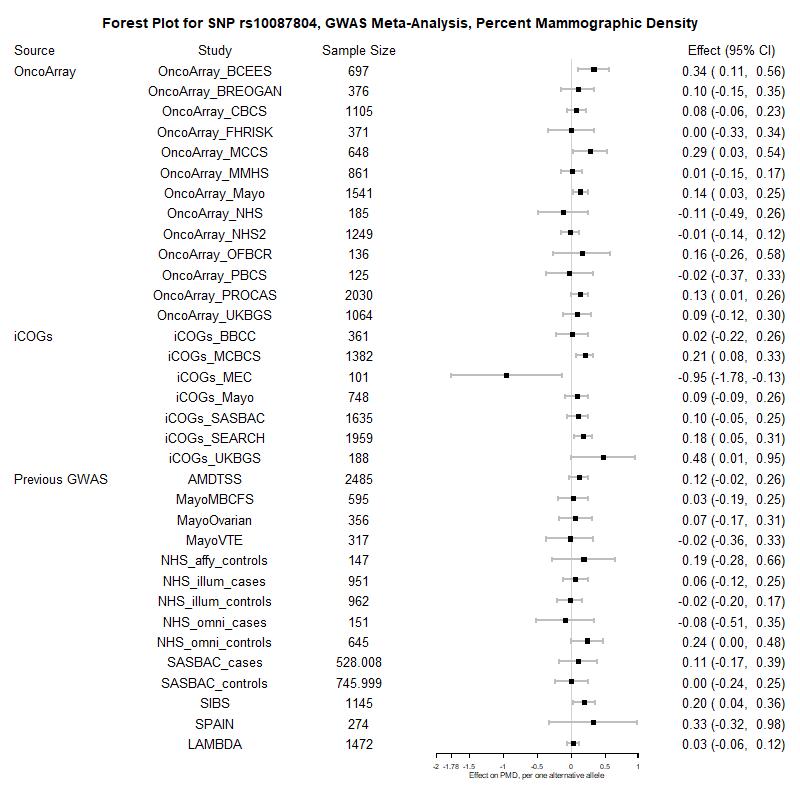


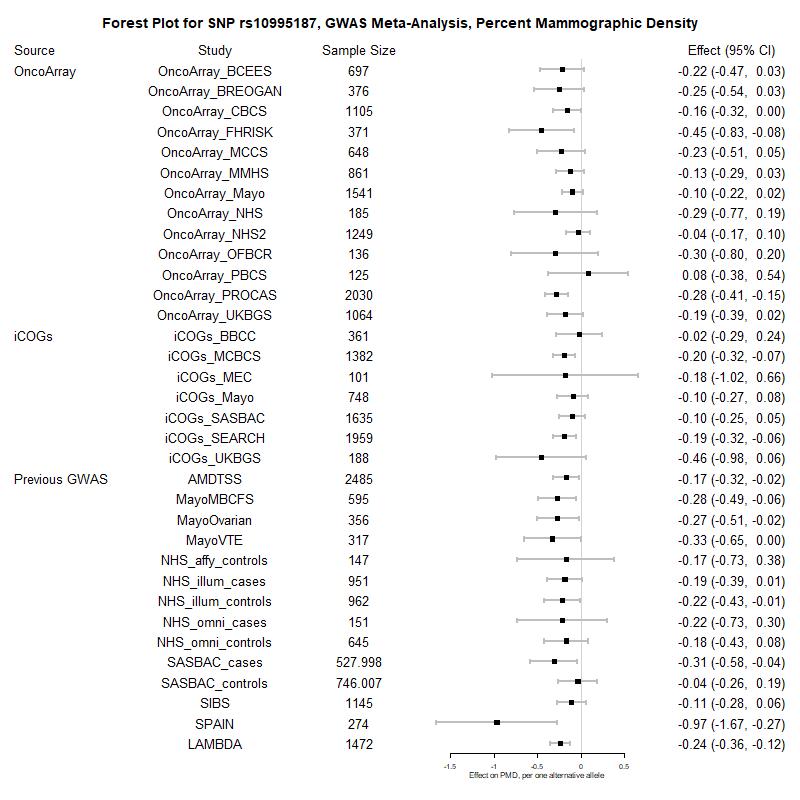


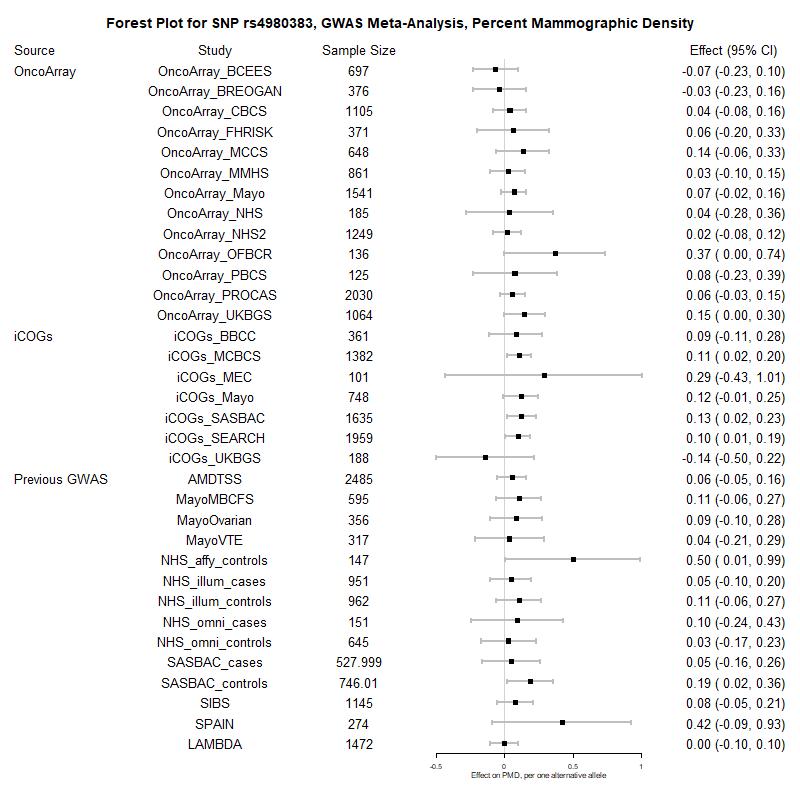


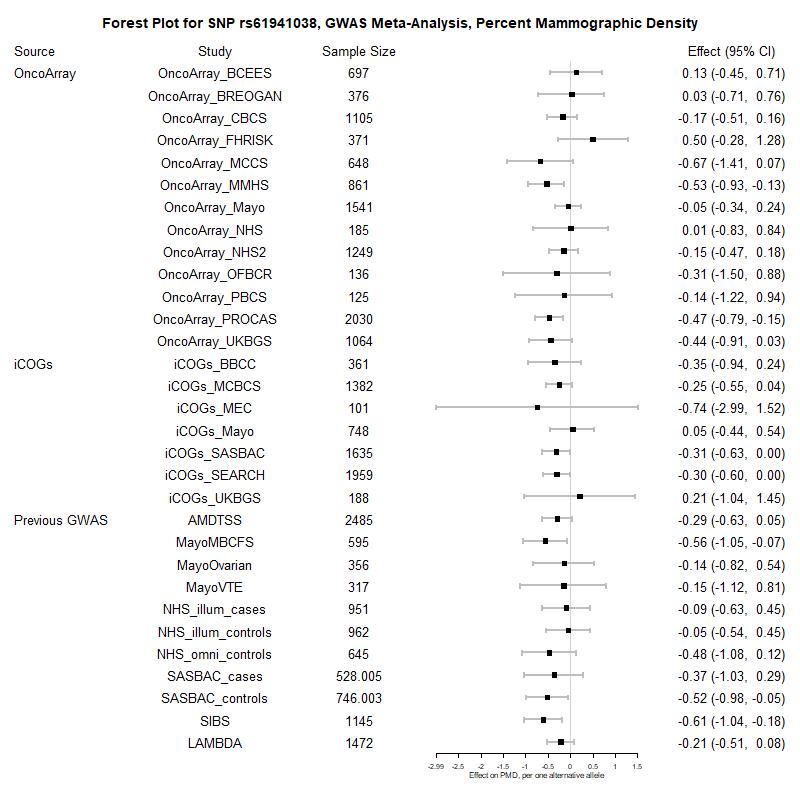


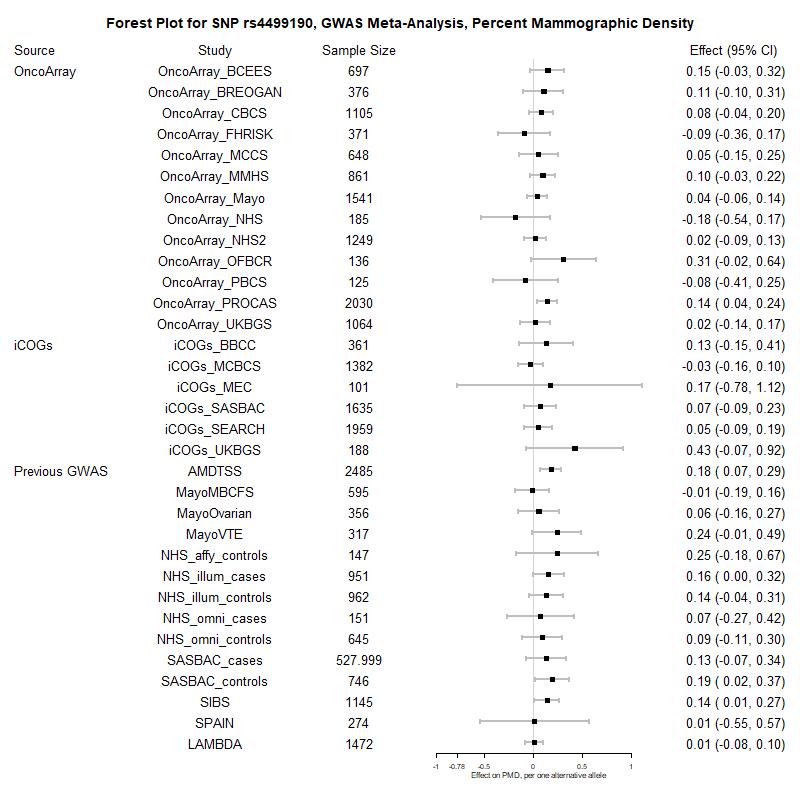


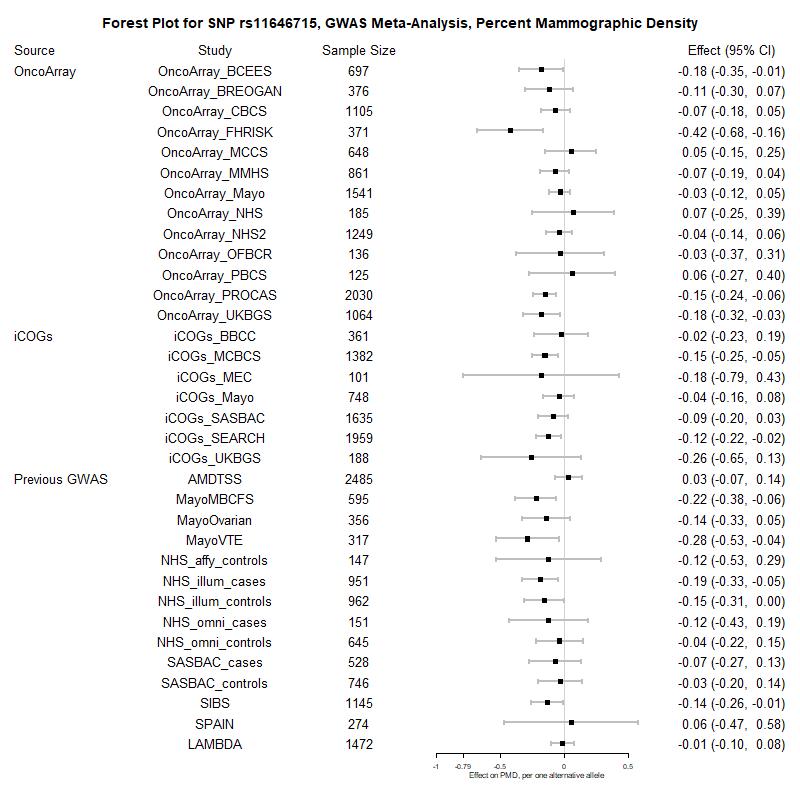


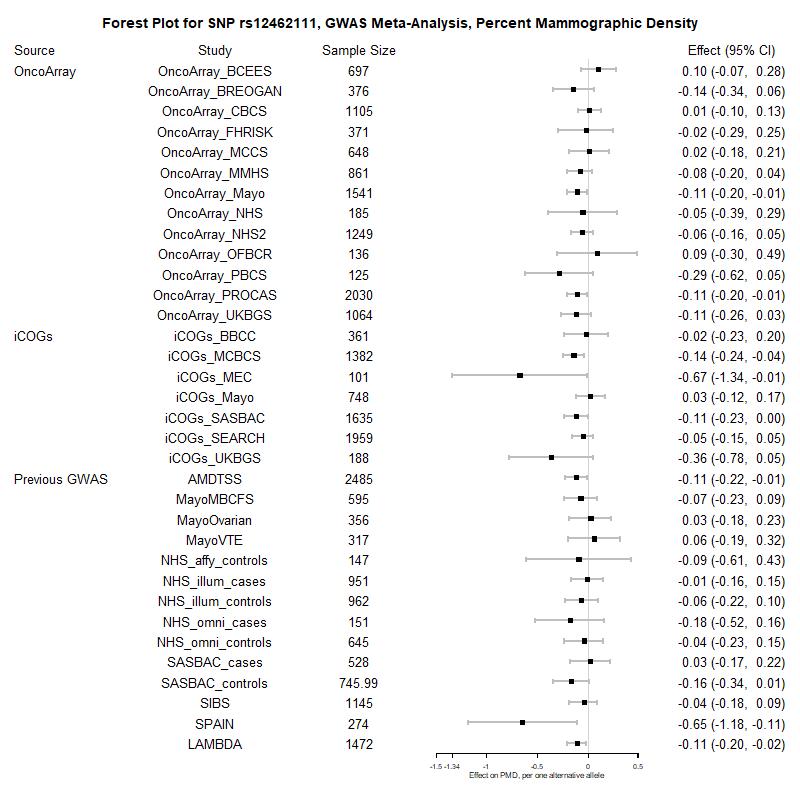


**Supplementary Figure 9.** Local genetic correlation estimates between the three MD phenotypes and overall breast cancer. For the analysis of each traits pair, the whole genome was partitioned into 1,703 regions. The GWAS summary statistics of MD phenotypes were generated from the model adjusting for age and BMI at mammogram as well as the first ten ancestry informative principal components. SNP-heritability shared by the two traits was estimated, and then used to estimate the local genetic covariance and correlation. Regions with statistically significant local genetic correlation (after adjusting for multiple comparison) is highlighted in blue or red.

**Local genetic correlation between DA and Overall breast cancer**


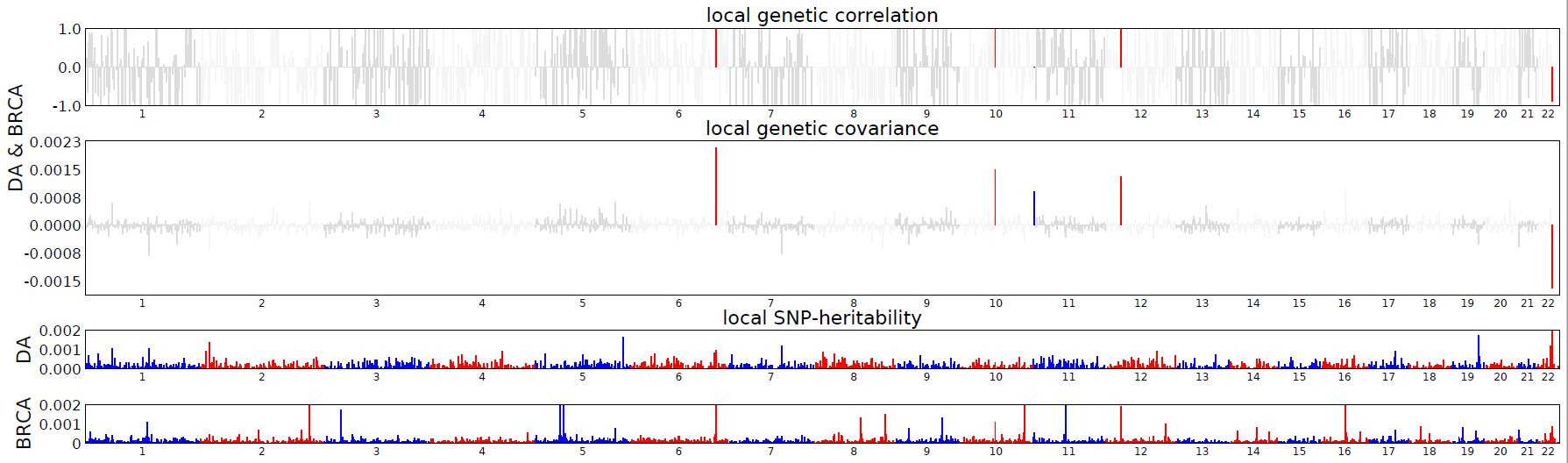


**Local genetic correlation between NDA and Overall breast cancer**


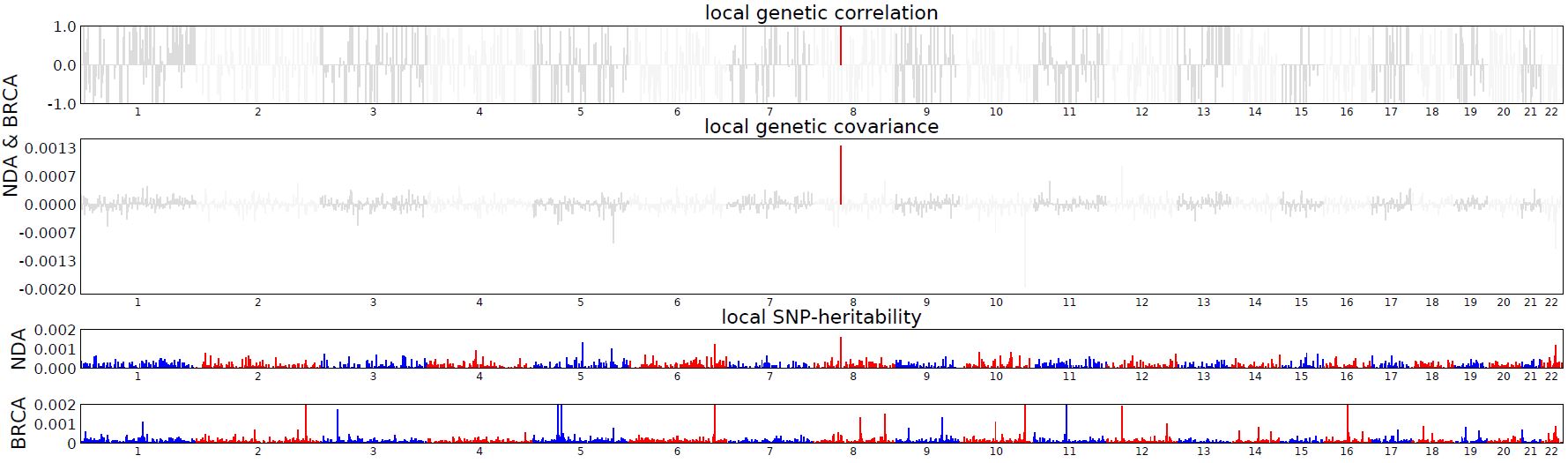


**Local genetic correlation between PMD and Overall breast cancer**


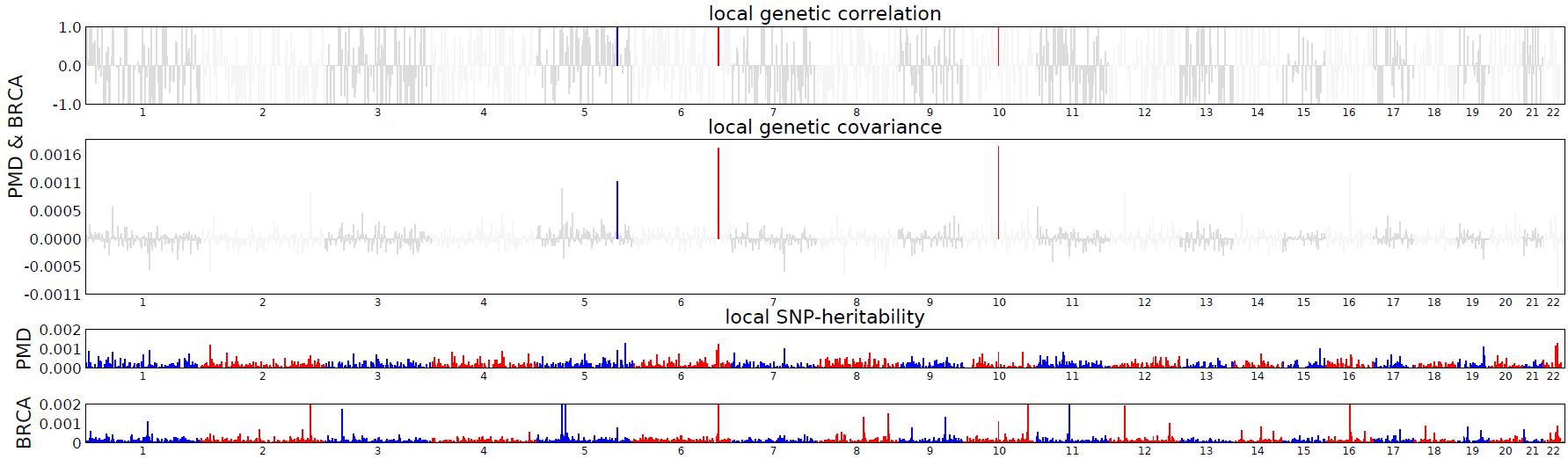


**Supplementary Figure 10.** Manhattan-like plots showing the TWAS Z-scores of association between genetically determined expression level of 7,882 genes and three MD phenotypes (DA, NDA and PMD). The GWAS summary statistics of MD phenotypes were generated from the model adjusting for age and BMI at mammogram as well as the first ten ancestry informative principal components. Z-score threshold (Z-score = +/- 4.743) corresponding to the statistical significance after adjusting for multiple comparison using Bonferroni correction (p = 0.05/(7,882*3) = 2.11x10^-6^) is shown by the horizontal red line. Significant genes are annotated.


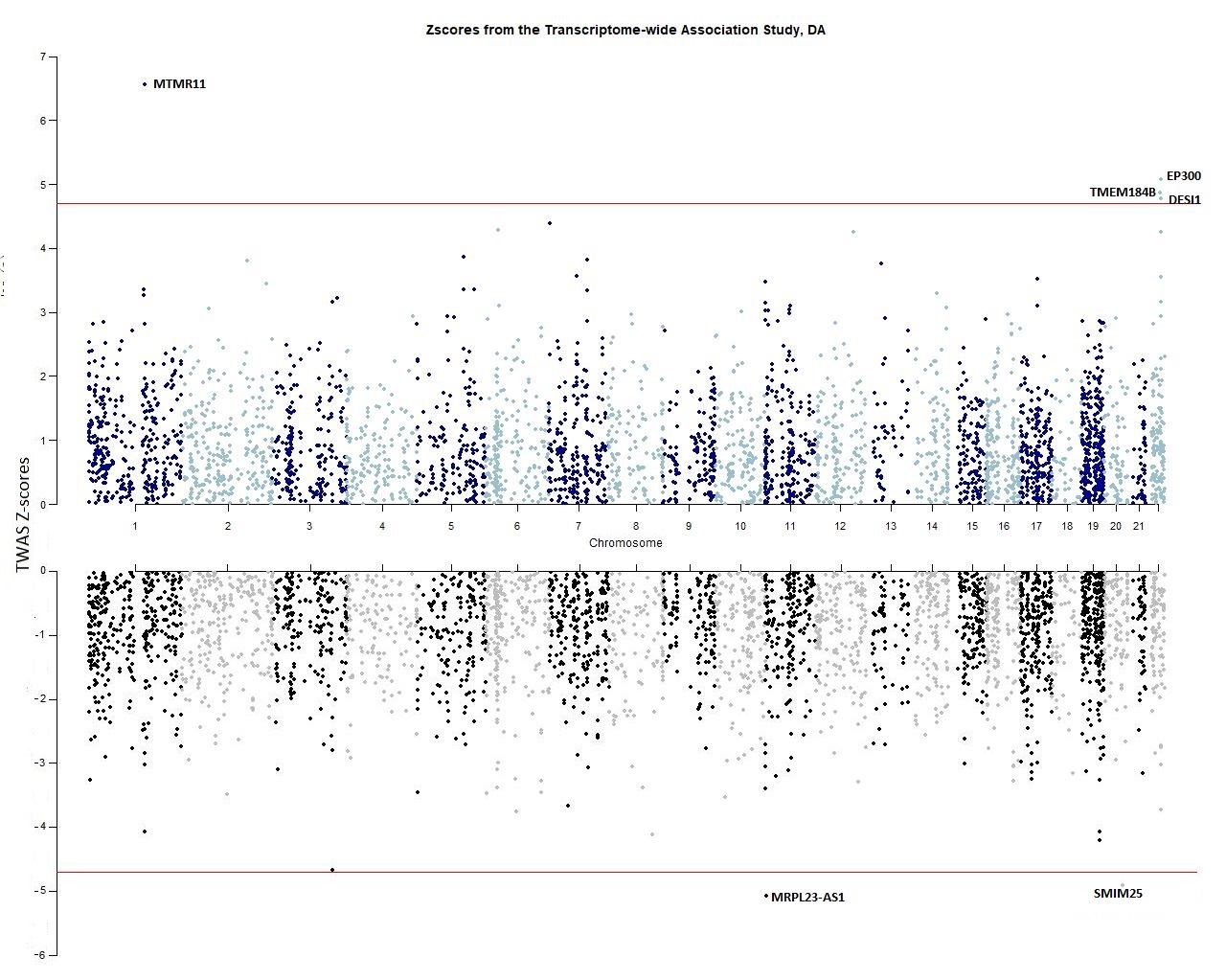


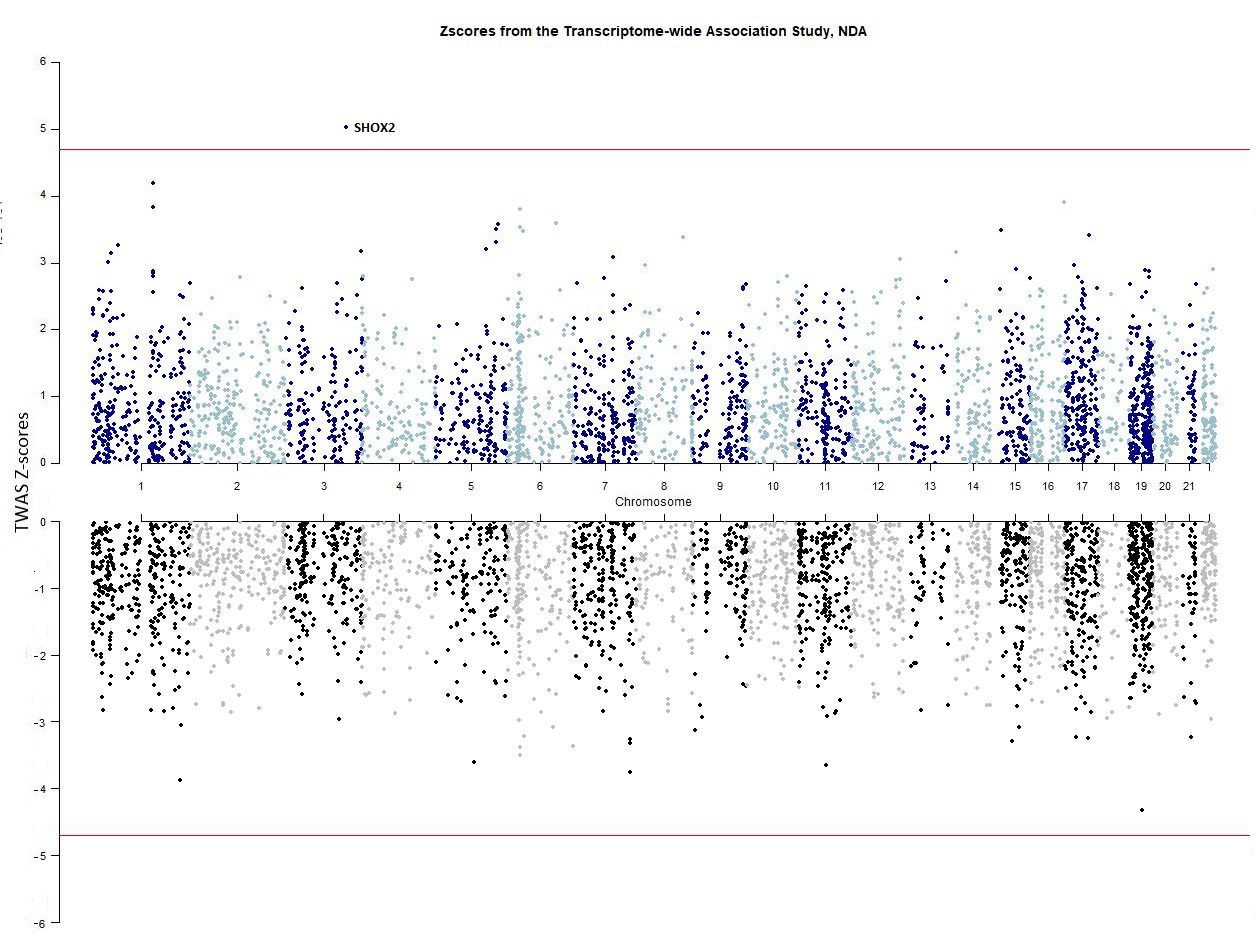

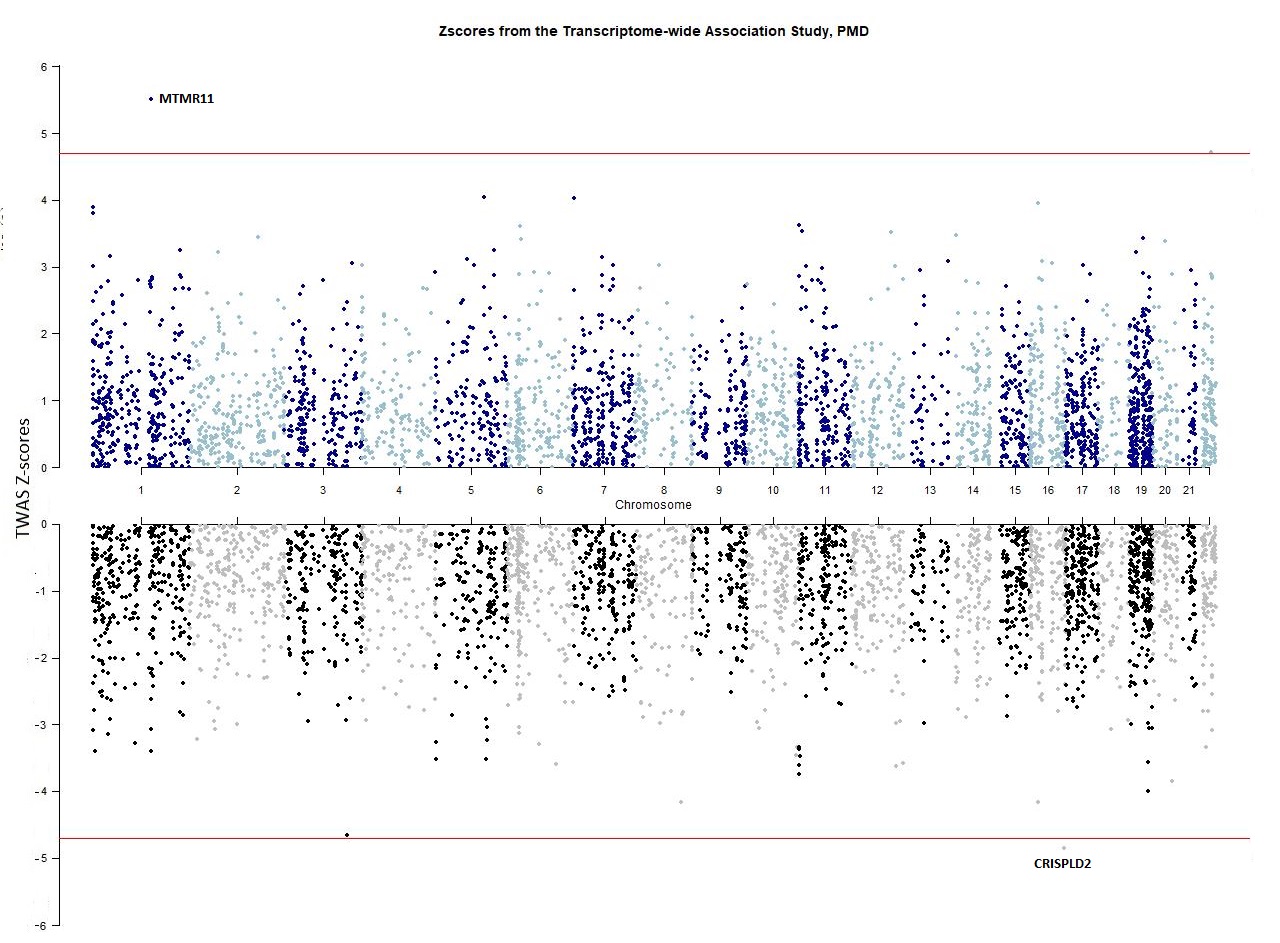

Supplement: Supplementary file 1 — Additional file 1. Supplementary Information, Funding, Acknowledgement, and Supplementary Figures. [file 13058_2022_1524_MOESM1_ESM.docx]
